# Between CERN and Cold Spring Harbor: Shaping the European Molecular Biology Laboratory (1967–1978)

**DOI:** 10.1007/s10739-026-09865-8

**Published:** 2026-08-12

**Authors:** Francesco Cassata

**Affiliations:** Department of Antiquities, Philosophy, History (DAFIST), University of Genoa, Genoa, Italy

**Keywords:** EMBO, EMBL, Molecular biology, Europeanization, Scientific cooperation, Science diplomacy

## Abstract

This article reconstructs the political, institutional, and scientific processes that led to the founding of the European Molecular Biology Laboratory (EMBL) in Heidelberg, tracing the trajectory of the EMBO Laboratory project from its substantial redesign in 1967 through the intergovernmental negotiations that culminated in the EMBL treaty of 1974. Against existing historiography centered on the early 1960s, the article proposes a revised chronology: the EMBO Laboratory was not designed in 1963–64 but in 1967–71, and its history cannot be understood without foregrounding the role of the European Molecular Biology Conference (EMBC) as the institutional and diplomatic arena through which the project was debated, reshaped, and ultimately realized. The first two sections examine two successive proposals—the “Nice proposal” (1967–68) and the “Konstanz proposal” (1969–70)—showing how they articulated the tension between centralization and decentralization in the Europeanization of molecular biology, and how EMBO functioned as an epistemic community that negotiated disciplinary boundaries, coordinated national interests, and mobilized political support among European governments. The third section analyzes the siting process, demonstrating how the choice of Heidelberg emerged from a conflictual interplay of intergovernmental competition, national research agendas, and local conditions. More broadly, the article situates the origins of EMBL within the specific dynamics of European integration in the late 1960s and early 1970s, arguing that the establishment of EMBC and EMBL was not only a product of this integration process but also an instrument of it.

## Introduction

The European Molecular Biology Organization (EMBO) was legally established in Geneva in 1964 as a private non-profit association. It originated as a network of self-appointed elite scientists from Western Europe and Israel, driven by Cambridge crystallographer John C. Kendrew, Nobel laureate in Chemistry in 1962, and led by a Council of fourteen other founding members. The EMBO Council was itself a reflection of the institutionalization of European molecular biology between the mid-1950s and the early 1960s.^1^ Its composition embodied the diversity of scientific backgrounds and experimental approaches,^2^ as well as the diffused distribution of research centers—many of them established outside the university system and with the support of centralized State agencies—that characterized the emerging “new biology.”^3^

In its institutional identity and foundational rhetoric, EMBO drew on the Cold War framework that positioned the budding discipline of molecular biology within the science policy discourses of the late 1950s and early 1960s, aligning it with prevailing visions of scientific modernity. From this perspective, molecular biology was portrayed as a fundamental new research enterprise of the Atomic Age, closely associated with physics and therefore requiring comparable levels of state support (Strasser 2002, pp. 530–531). It was also described as an increasingly “American science” that needed to be actively promoted in Europe in order to reverse national scientific decline, bridge the “gap” with the United States, and counter the transatlantic scientific “brain drain.”^4^ Finally, molecular biology was presented as an interdisciplinary hybrid that transcended traditional disciplinary boundaries, and thus demanded both national and international funding to overcome resistance from the more conservative university system (Abir-Am 1992, p. 169).

While EMBO was shaped by what John Krige (2006) has defined as the Cold War co-production of American hegemony in Western European science, the rationale for its establishment also reflected a specifically European context, characterized by the postwar economic boom and by “a complex intra-European political web in which both cooperation and competition were essential” (Baneke 2015, p. 169). To meet the “American challenge” and close the “gap” with the United States, EMBO proposed not to resist the “American model” but rather to translate it into an intra-European dynamic.

More specifically, EMBO envisaged two different tools: first, a decentralized network of existing laboratories and research centers, the European Federal Biological Organization (EFBO, re–labelled as “Foundation” in the EMBO statutes), designed to facilitate exchanges of scientists through short-term and long-term (post-doctoral) fellowships, visiting professorships, advanced courses, and workshops; second, a centralized structure, the European Laboratory of Molecular Biology (initially referred to as CERB, by analogy with CERN, or more simply as the “EMBO Laboratory”).

Two distinct analogies served as political and symbolic resources through which tensions between centralization and decentralization were articulated. On the one hand, Cold Spring Harbor—whose courses and symposia in the 1940s and 1950s had been crucial to the development of the emerging field of molecular biology—was invoked as a model for international scientific exchange and advanced training rather than as a permanent institutional structure; on the other hand, CERN was consistently presented as a model of technoscientific Europeanism centered on a major intergovernmental infrastructure.^5^

Elaborated in 1963–64 by John Kendrew—for the centralized laboratory—and Conrad H. Waddington—for the decentralized “Foundation”—following informal meetings held in Geneva and Ravello (Krige 2002; Strasser 2003; Cassata 2015), this dual program was formally incorporated in the EMBO Statutes in 1964 and into an official booklet—the “EMBO Policy Document”—in 1965.^6^

From the outset, EMBO’s scientific and diplomatic strategy was conceived as a stepwise process. The decentralized network and the central laboratory were not intended as radical alternatives, but as complementary stages in the Europeanization of molecular biology:^7^ the decentralized network was to precede and prepare the establishment of the central laboratory (Cassata 2015). When EMBO decided to seek funding directly from European governments—through the mediation of the Swiss Department of Foreign Affairs (the “Swiss initiative,” as it was called)—in 1965–66, priority was given to the “Foundation” project, while the laboratory project was temporarily postponed. In 1969, after years of complex negotiations,^8^ the “Swiss initiative” succeeded and a new intergovernmental organization—the European Molecular Biology Conference (EMBC)—was established, providing EMBO with financial stability and political legitimacy: the EMBC supported EMBO scientific program, including fellowships, visiting professorships, courses and workshops, and also ensured funding for the preliminary studies of the EMBO Laboratory project as an EMBC “special project” (Cassata 2026).

Historical accounts have explored in detail the origins of EMBO and EMBC, reconstructing the institutional, political, and social context of European molecular biology in the early 1960s (de Chadarevian and Strasser 2002; Strasser 2002, 2003) and explaining why the laboratory project, although already conceived at that time, did not initially take off (Krige 2002; Cassata 2015).

However, within the complex triad that structured (and still defines) the institutional architecture of molecular biology in Europe—EMBO, EMBC, and EMBL—the latter has remained comparatively unexplored (EMBL 1994; Morange 1997; Krige 2013). By focusing primarily on the initial version of the laboratory project elaborated between 1963 and 1965, existing reconstructions largely overlook the significant transformations the project underwent in the second half of the 1960s and do not fully explain why it finally materialized in 1974, when ten EMBC member states signed the international treaty that established the European Molecular Biology Laboratory (EMBL) in Heidelberg.^9^ Chronologically and conceptually restricting the analysis of the EMBO Laboratory project to the first half of the 1960s entails two major shortcomings.^10^ First, it produces a predominantly British–oriented narrative, paradoxically obscuring the collective, *Western European* dimension of the EMBO Laboratory project. The role of German scientists and policy makers, for instance, is almost entirely absent from these accounts, making the final landing of the laboratory in Heidelberg difficult to explain. Second, it underestimates the institutional innovation represented by the EMBC and its interaction with EMBO. Far from serving merely as a funding mechanism for EMBO fellowships and courses, the EMBC provided an institutional and diplomatic channel for negotiating the laboratory project at a European intergovernmental level. It was also a precedent and a legal model for the international treaty that established EMBL in 1974.

To provide a more comprehensive analysis of the origins of the EMBL, this article proposes a revised chronology: the EMBO Laboratory was not designed in 1963–65 but in 1967–71. Moreover, the project was never monolithic or static. The first version—referred to here as the “Nice proposal”—was substantially revised following the formal establishment of the EMBC in 1969, giving rise to a second and considerably different version, referred to here as the “Konstanz proposal.”

Resetting the chronology is not merely a matter of philological precision. On the contrary, it carries deeper conceptual implications. Focusing on the late 1960s and early 1970s means, above all, situating the origins of the EMBO Laboratory within the specific dynamics of European integration during that period: the waning of US influence in Western Europe after the first two postwar decades; the gradual enlargement of the European Communities (EC) following the resignation of French President Charles de Gaulle, who had blocked this process throughout the 1960s;^11^ Britain’s accession to the EC alongside Denmark and Ireland in 1973; the growing economic strength and political self-confidence of the Federal Republic of Germany (hereafter, West Germany). The establishment of two intergovernmental organizations in the field of molecular biology—in 1970 (EMBC) and 1974 (EMBL)—was not only a product of this integration process but also an instrument of it. A comparative look at the founding members of the EMBC and EMBL is revealing in this regard (Table 1).

**Table 1:** EMBC and EMBL membership in comparative perspective: European communities, Council of Europe, and OECD

Council of Europe (1949)	European communities (1951/1957)	OECD (1961)	EMBC (1969-70)	EMBL (1973-74)
Belgium, Denmark, France, Ireland, Italy, Luxembourg, Netherlands, Norway, Sweden, United Kingdom	Belgium, Federal Republic of Germany, France, Italy, Luxembourg, Netherlands	Austria, Belgium, Canada, Denmark, Federal Republic of Germany, France, Greece, Iceland, Ireland, Italy, Luxembourg, Netherlands, Norway, Portugal, Spain, Sweden, Switzerland, Turkey, United Kingdom, United States	Austria, Belgium (through the *Fonds National pour la recherche scientifique*, FNRS), Denmark, Federal Republic of Germany, France, Greece, Israel, Italy, Netherlands, Norway, Spain, Sweden, Switzerland, United Kingdom	Austria, Denmark, Federal Republic of Germany, France, Israel, Italy, Netherlands, Sweden, Switzerland, United Kingdom

On the one hand, it shows that the “Europe” of molecular biology did not overlap with that of the EC and other fora such as the Organization for Economic Co-operation and Development (OECD) and the Council of Europe: it included neutral states (Austria, Switzerland, Sweden), Israel, and numerous non-EC members. On the other, it demonstrates how scientific cooperation in a de-militarized and relatively inexpensive field such as molecular biology could itself become a “Europeanizing factor” (Forgiarini 2024, p. 217), a catalyst for political integration and eventually a compensation for setbacks in other areas (Misa and Schot 2005, p. 13).

Secondly, recontextualizing the origins of the EMBO Laboratory in the late 1960s and early 1970s contributes further to a history of Europeanization in science and research that challenges “the EC-centric path toward European unity” (Mitzner 2020, p. 4). Research became a full-fledged EC policy only in the 1980s. As the case of molecular biology clearly illustrates, European cooperation and competition in science and research policy developed outside the EC framework, involved a wide range of actors and initiatives, and was not inevitably directed toward the EC/EU as a uniform and teleological outcome. When the EMBO Laboratory was being shaped and negotiated, numerous competing models of European scientific cooperation were in play: bilateral agreements such as the European Science Exchange Programme (ESEP), established by the Royal Society in 1967 (Collins 2016, pp. 205–214); early proposals for a European Research Council, launched in 1968 within the OECD context (Collins 2016, pp. 217–218); non-governmental organizations such as the European Science Foundation (ESF), founded in 1974 (Wolff 2018; Unger 2020). Within this varied landscape, the case of molecular biology is particularly significant as it produced the distinctive outcome of two intergovernmental bodies—EMBC and EMBL—that channeled public funding into the activities of a private scientific association (EMBO) focused on a single research field.

Finally, this article shows how Europeanization shaped the molecularization of life sciences in the late 1960s and early 1970s. The process leading to the establishment of the EMBO Laboratory coincided with the period that historian Michel Morange defined as the “crossing of the desert” in the history of molecular biology (Morange 2003, pp. 216–237): a phase of Kuhnian normal science, in his view, but also one of epistemic impasse, rooted in the difficulties of moving from the study of viruses and bacteria to that of multicellular organisms. Yet, as Morange himself has acknowledged (Morange 2021, p. 181), this phase was not simply a desert or a vacuum, it was also a significant moment of expansion, as molecular approaches and methods extended into cell biology, embryology, immunology, and neurobiology (Morange 2020, p. 175).

From this perspective, the long road to the EMBO Laboratory may be understood as a creative and flexible, if selective, response to this moment of epistemic crisis and institutional expansion—and as an analytical prism through which to observe the broadening of the molecular vista in 20th-century biology (Grote 2019; Grote et al. 2021). Far from being merely a European reproduction of the Cambridge MRC Laboratory under Kendrew’s leadership, the EMBO Laboratory emerged from the intermingling of distinct, locally entrenched research traditions (Rheinberger 2012, p. 742), which crystallized along both scientific and political lines. In 1967–68, the EMBO Laboratory proposal—resulting primarily from collaboration between the Cambridge and Pasteur groups together with the Max Planck Institute (MPI) for Biophysical Chemistry in Göttingen—envisaged an integrated combination of structural biology (X-ray diffraction, electron microscopy, nuclear magnetic resonance), molecular genetics, and biochemistry, extended to membrane chemistry, immunology, and neurophysiology. By 1969–70, the proposal had been reframed around a new British–German focus on advanced biophysical and biochemical instrumentation and computation, oriented toward cell genetics and subcellular structures (membranes and macromolecular assemblies).

Diplomatic negotiations and epistemic reconfigurations were closely intertwined through the institutional interaction between EMBO and EMBC, and through the mediation of a network of knowledge–based experts (the EMBO Council and committees). These actors defined the boundaries of molecular biology, monitored discipline-building at the national level, and drew together diverse research traditions into a hybrid European project.

The following three sections trace this process in detail. The first two examine the two different EMBO Laboratory projects developed between 1967 and 1970, while the third analyzes the complex siting process that culminated in the founding of the EMBL in Heidelberg.

## On the French Riviera: The “Nice Proposal” (1967–1968)

In its initial version, the EMBO Laboratory project—written by Kendrew in 1963 for the Ravello meeting and slightly revised in 1965 for inclusion in the “EMBO Policy Document”—was no more than a five–page draft. In it, Kendrew outlined two main arguments justifying “the case for a large European Laboratory for molecular biology” (EMBO 1966, p. 15).

First, as an inherently multidisciplinary field, molecular biology required large-scale infrastructure capable of fostering cross-fertilization and day-to-day exchange among specialists from different areas and technical backgrounds. Resorting to analogies with CERN, Kendrew argued that, in molecular biology, the equivalent of CERN’s expensive machinery was “an interacting group of differing techniques and talents, each only moderately expensive by itself, but in sum costing so much as to be beyond most national resources, financial or – especially – human” (EMBO 1966, pp. 15–16). Second, the proposed European Laboratory for molecular biology would provide post-doctoral training facilities and opportunities “hardly available in Europe,” thereby improving intra-European mobility among young researchers and preventing the “brain drain” to the United States. Here again, Kendrew invoked the CERN analogy: rather than draining national universities, the European Laboratory—like CERN—would stimulate “the universities of Europe to establish posts and departments” of molecular biology (EMBO 1966, p. 16).

The technical details of the project were rather sketchy. The European laboratory should include fourteen research groups (just listed, without description),^12^ for a total staff of 150 scientists minimum, 10–15% of them on a permanent or long-term appointment. The project was also expensive: the start-up cost of the laboratory and its equipment was estimated to be about $5 million and its annual running costs to be about $2 million (about $50 and $20 million today).

Submitted for consideration to the EMBC delegations at the meeting of April 1967, the EMBO Laboratory project received strong support from France and Italy. François Jacob, Nobel Prize in Physiology or Medicine in 1965 (together with his colleagues at the Pasteur Institute, Jacques Monod and André Lwoff), and Adriano Buzzati-Traverso, head of the LIGB in Naples, leveraged their dual roles as EMBO Council members and EMBC delegates to promote the EMBO Laboratory project as a means of reinforcing their research agendas at the national level. In particular, Jacob reported the position of the French interministerial working group on molecular biology, which was prepared to channel part of the governmental funds from the *Action concertée “Biologie moléculaire”* into a European laboratory to be established on French soil, in the region of Nice.^13^ The French–Italian position encountered strong opposition from Great Britain, Sweden, and West Germany, whose representatives considered the project insufficiently detailed and, overall, premature in terms of timing and financing. The most vocal criticism came from the UK. Referring to advice received from the Royal Society and the Medical Research Council, the British delegation argued that EMBO programs would fragment the European scientific community along disciplinary lines while draining resources from national university systems. As a result of these tensions, the EMBC invited EMBO to prepare a more detailed project, without referring to the support provided by France and other European governments.^14^

The revision of the EMBO Laboratory project began immediately after the April conference and involved, in addition to Kendrew, a selected group of EMBO leading figures: the EMBO Secretary General Jeffries Wyman, a Harvard–trained biochemist and former US science attaché in Paris, then associated with the Regina Elena Institute for Cancer Research in Rome and the Biochemistry Institute of La Sapienza University, but collaborating with Jacques Monod and Jean-Pierre Changeux on protein allostery; the EMBO Executive Secretary Raymond K. Appleyard, a British biophysicist and head of EURATOM Biology Division;^15^ the EMBO Council member François Jacob; the biochemist Alfred Tissières, from the University of Geneva; the biochemist Hubert Chantrenne, from Brachet’s research group in Brussels; Manfred Eigen, Nobel laureate in Chemistry in 1967, who was laying the foundation of the MPI for Biophysical Chemistry in Göttingen; and Oxford biochemist and immunologist Rodney R. Porter. Chantrenne, Jacob and Tissières were also members of the EMBO Laboratory Committee, established in 1963 but almost inactive until 1967.^16^

The new proposal for the EMBO Laboratory was framed in April–May 1967 through an informal exchange of letters. This epistolary brainstorming, often emerging from discussions and surveys conducted within the respective national contexts, was instrumental in bringing together different research schools and traditions into a shared project. It also revealed an inherent tension between those who envisioned the future EMBO Laboratory primarily as a networking structure focused on postdoctoral training and those who regarded it as essentially a research institution centered on advanced instrumentation.

It was Jeffries Wyman who first initiated the discussion by sharing with Kendrew his thoughts on a renewed program for the laboratory proposal. In his letter, Wyman emphasized the need to conceive of the laboratory not merely as “a more complete assemblage of instruments,” but as a “qualitatively different” research endeavor, “operating on a larger scale with full command of the resources of modern engineering and industry,” and including protein synthesis, large-scale preparation of enzymes and cultures of micro-organisms, studies of structure and organization above the macromolecular level, and the analysis of “the storage, assortment and recombination of information in the central nervous system” through access to “large computer resources.”^17^

In responding to Wyman, on April 19, Kendrew brought Appleyard, Eigen, Jacob, Porter, and Tissières into the conversation. According to Kendrew, it was time “to begin getting straight our ideas on an international laboratory.” For this purpose, it was “politically important to think of research projects which could only, or could much more efficiently, be done in a central laboratory.”^18^ Many ideas were already on the table: Kendrew specifically mentioned Francis Crick’s project of a world-wide intensive and exhaustive study of a single-celled organism, the bacterium *E. coli*—the so-called “Project K” (Cobb 2025, p. 256)—and referred to Wyman’s recent suggestions. He concluded by requesting comments and “any general ideas” on the “whole question of the laboratory,” to be collected and used for “as the basis for a wider enquiry.”^19^

François Jacob’s reaction summarized the main points of a meeting he had had with Monod and the “young people” at the Pasteur Institute regarding the proposed “European laboratory.” While endorsing the projects already suggested by Wyman and Kendrew, Jacob and fellow Pasteurians highlighted the need for a “Center of common formation” to promote intra-European exchange among researchers with diverse areas of expertise. According to Jacob, such a laboratory would not only reduce the emigration of young European researchers to the US but might also encourage a kind of “reverse brain drain,” making US–based European biologists more willing to come back to Europe. For this purpose, Jacob suggested they “obtain from people such as Delbrück, Luria, Dulbecco, etc., a letter stating that they would consider spending some time in a European lab.”^20^

Like Jacob, Tissières also believed that the European laboratory could be justified as a center for “increased exchange of information and cooperation,” helping to bridge “the gap between the rates of growth of modern biology in the US and in Europe” and provide appropriate training for PhD candidates and postdoctoral students. He found it difficult to legitimize the laboratory “on the basis of large scale preparation of microorganisms, enzymes, etc.,”^21^ while he considered “a vast program on the molecular biology of animal cells” more appropriate: such a program “would, either from the start, or later, include the nervous system project.”^22^

Manfred Eigen’s response was based on a broad survey conducted among twenty German scientists working “in the field of molecular biology.” They were asked to respond to two questions:

- Do the scientists think that there is a need for a European Institute of Molecular Biology or do they rather think that the same work could be done just as well in national institutions with some international cooperation;
- Furthermore: Who would be willing to work full or part time in this institute.^23^

According to Eigen, all the interviewees agreed that “the institute would fulfill a requirement which could not be met by any of the existing national institutions.” However, as they were all in well–established positions, they were not ready to commit to full–time appointments at the proposed “European institute.” Nevertheless, “some did not exclude such a possibility, and all declared that in principle they would be willing to spend some time of the year there.” Regarding the scientific program of the EMBO Laboratory, Eigen and his colleagues drew analogies with CERN and particularly emphasized the importance of “big machines”—for instance, electron microscopes with higher resolution, or automated computer systems for chemical sequence analysis of proteins and nucleic acids. Although the “exchange of ideas among scientists” was considered crucial, Eigen argued that it would have been difficult “to persuade governments to spend money solely for this purpose.”^24^ Like Eigen, Oxford biochemist Rodney R. Porter also deemed that expensive, large-scale instrumentation, “not justifiable on a national base,” was the only “overwhelming argument for a European laboratory.”^25^

In his response to Kendrew’s survey, on June 20, Raymond K. Appleyard began elaborating the first sketch of the scientific program of the new EMBO Laboratory proposal. According to Appleyard, the laboratory needed “a target something big enough, broad enough and imaginative enough to captivate Governments, to be an umbrella for anything one might really want to do, and to ease recruitment.” The project had to be such as “to allow the German and like-minded Governments to say officially and openly, this is something which cannot be done nationally: only supra nationally.” There was just “one obvious candidate” to fit this bill: a twin research target combining the “complete detailed understanding of a single bacterium” and the “general molecular comprehension of the special life processes of higher cells.” These two “complementary facets of the next phase of advance of molecular biology” had to be pursued “in mutual fertilization” and “at central leading point under one roof.” The range of requirements involved—from computers to pathogen-free animals—and the “need for an immediate cross-fertilization of ideas across this range” implied a level of “intellectual promiscuity only conceivable through close geographical intimacy.” “No institute in Europe” could satisfy these requirements and lead such a “twin assault.”^26^

In addition to a number of “particular programmes” dedicated to this “dual general objective,” Appleyard mentioned other specific activities through which the EMBO Laboratory could contribute “to strengthening other purely national Institutes:” as a provider of expensive and large-scale facilities (“syntheses, analyses, cultures and instruments”); as a training center for “young research workers,” forming “a common pool of ideas and understanding;” and as a scientific attractor, capable of “restraining and even reversing the ‘brain drain’ to America.”^27^

The initial cluster of ideas that had emerged in response to Kendrew’s letter of April 19 coalesced into a more concrete proposal in May–June 1967. To help coordinate this process, Appleyard seconded Marc Delauche—a French administrator working at the EURATOM offices in Brussels—to EMBO.^28^ Delauche was also asked by Kendrew to collect information about comparative advantages of possible sites for the laboratory, identified at this stage in “Nice, Geneva across the French frontier, the Vaud site near Geneva, Cambridge, Vienna.”^29^ Following an intense tour that included visits to Kendrew, Wyman, Tissières, Chantrenne, and Jacob, Delauche summarized the suggestions received and outlined a roadmap for developing a new proposal of the EMBO Laboratory.^30^ Two preliminary draft documents were needed: the laboratory scientific program, and a letter to the European molecular biologists who had “emigrated” to the United States. These documents were intended to form the agenda for a meeting of the EMBO Laboratory Committee, scheduled to take place in September 1967.^31^

**Fig. 1:**
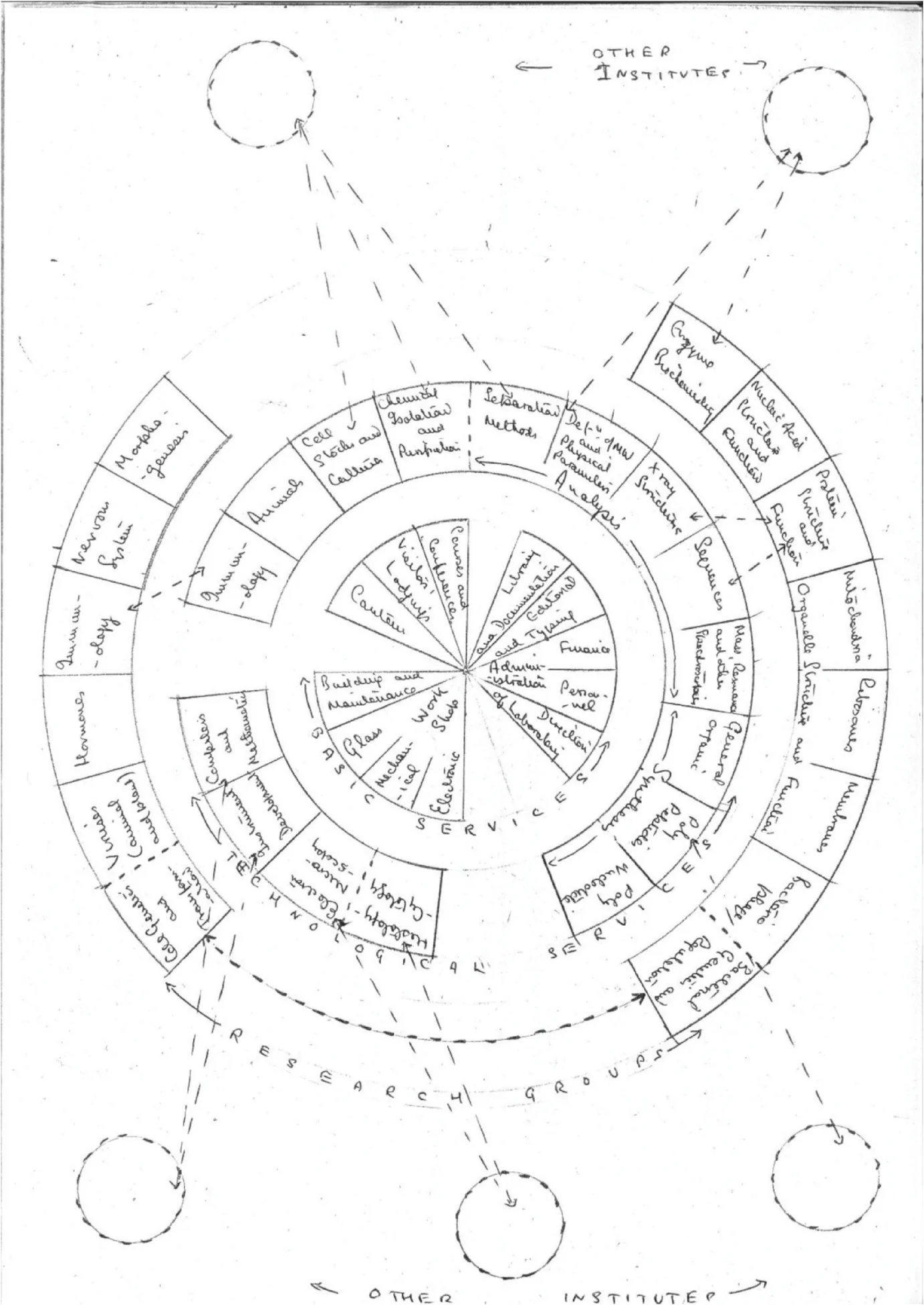
Raymond K. Appleyard, “Proposal for a European Laboratory of Molecular Biology, July 12, 1967,” first draft. Appleyard’s diagram. EMBO Archive, EMBO Laboratory Committee Materials, EMBO EMBOLAB-B-2-4, https://archive.embo.org/index.php/proposal-for-a-european-laboratory-of-molecular-biology. CC-BY

The task of preparing the first draft of the EMBO Laboratory proposal was assigned to Appleyard.^32^ As an administrative figure with significant expertise in European science policy—and, in Jacob’s words, neither a “moleculist” nor a “cellulist” by training^33^—Appleyard was well positioned to facilitate mediation between the different research approaches represented on the EMBO Laboratory Committee. Conversely, since 1963 Appleyard had been one of the most vocal opponents of Kendrew’s project for a single central laboratory, instead advocating a federative “polycephalic support of research,”^34^ in keeping with his experience as head of the EURATOM Biology Division (Cassata 2015, pp. 566–569). Not surprisingly, then, Appleyard ended up inflecting the EMBO Laboratory project with decentralizing elements, presenting the proposed single “European centre” as a node within a broader network (Fig. 1).^35^

In Appleyard’s view, the role of the central European Laboratory was defined by four main features. First, it could serve as a “programmatic focus,” concentrating “attention, means and men” on the “next major targets of European molecular biology.”^36^ Second, it could offer “technological support,” making a “complete technological base fully accessible to European molecular biology as a whole,” while maintaining “purpose, dynamism and direction” through the connection between the facility and its own research programs. Third, it could function as a “training ground” for a “new generation of young men.” Finally, it could act as an “integrator” of related research lines, promoting the “cross-fertilization between individual areas” that was essential for progress but difficult to achieve by other means.^37^ Such a laboratory was beyond the means of any single European nation. Outside Europe, partial realizations of this model, “with already many advantages and considerable success,” could only be found in the United States, for example, “the very large groupings at MIT Harvard or at Stanford; the easy ‘horizontal’ links between problems at Cal.Tech.; the massive technological support at Oak Ridge.”^38^

With regard to the scientific program, the “natural target” for the European Laboratory was the “combined pursuit under one roof” of the complete understanding of one suitable cell (the bacterium *E. coli*) and of the specialized functions and features of other differentiated cells.^39^ From this perspective, the design of the EMBO Laboratory was articulated into three sections: first, “research groups,” offering “new knowledge and/or new methods” and staffed by experimental scientists;^40^ second, “technological services,” providing the “best possible facilities” and staffed by engineers;^41^ finally, some “basic services,” common to all, including space for courses and conferences.

Between late August and early September 1967,^42^ Kendrew and Wyman produced a second “draft document for Governments about the laboratory,” conceived as “an alternative to the Appleyard draft.”^43^ Drawing heavily on quotations from the 1965 “EMBO Policy Document,” with the aim of demonstrating consistency with the past,^44^ the Kendrew–Wyman document differed markedly from Appleyard’s. It was not only a scientific program but also a science policy tool, emphasizing both the centralizing role of the EMBO Laboratory project—drawing on the CERN analogy—and its federative function, expressed through the “services” it was to provide to national university systems in research and postdoctoral training.

The first section of the Kendrew–Wyman document was entitled “Biology at the molecular level and its prospects.”^45^ Biology “at the molecular level,” rather than “molecular biology,” was the phrase that Kendrew had adopted in Britain for the survey and report of the Council for Scientific Policy (CSP) Working Party on Molecular Biology, of which he was chairman (de Chadarevian 2002, p. 316). Defining the subject in terms of “biology at the molecular level” aimed at instilling the molecular approach into more traditional and already institutionalized research fields within university systems, above all biochemistry (Abir-Am 1992, p. 171; de Chadarevian 2002, p. 320). By extending this label from the British to the European arena, the Kendrew–Wyman document presented all biology as, *tout court*, molecular biology, while envisaging the future EMBO Laboratory as an innovative and transdisciplinary institution for the molecularization of the life sciences open to “biochemists, physiologists, pharmacologists, cell biologists.”^46^

The subsequent section addressed the question: “Why an international laboratory?”^47^ Alongside lengthy quotations from the 1965 “EMBO Policy Document,” concerning the “basic philosophy lying behind the proposal for an international laboratory,”^48^ the Kendrew–Wyman proposal added “more specific arguments” designed to forestall potential opposition. The document emphasized the “valuable functions” the EMBO Laboratory could perform in terms of recruitment, training and eventual employment of scientists and technicians. Based on a small group of permanent full-time scientists and a high turn-over of visiting researchers, the EMBO Laboratory aimed to create “a real European community of scientists.”^49^ Referring to the experience of CERN—described as “the analogous enterprise in physics”—Kendrew and Wyman argued that the EMBO Laboratory, rather than “starving the universities of trained biologists,” would have “exactly the reverse effect.”^50^ With its “complete set of instrumental facilities,” the laboratory could stimulate research requiring expensive instrumentation or a “co-operative attack,” while also providing both basic and advanced training to PhD candidates and postdoctoral researchers in molecular biology.^51^

The third section focused on the scientific program, drawing on Appleyard’s idea of the dual approach. While essentially relying on Crick’s “Project K” for the total understanding of a single, unicellular organism, Kendrew and Wyman specified the second target, referring to “the study of the recording, storage, transfer and assortment of biological information,” particularly in areas such as the control mechanisms, the antibody responses, and the mechanisms of the nervous system in higher animals.^52^ At this stage, the description of the research groups was deliberately left vague and open-ended. However, as in Appleyard’s draft, the Kendrew–Wyman document listed four “technological services” that the central laboratory could offer to benefit the “biological community at large:” large-scale growth of pure strains of micro-organisms; facilities for extraction and purification of large amount of cell constituents; instrumentation research; and large-scale computer automation.^53^

A “number of appendices,” written by “scientific authorities” to support the case for the EMBO Laboratory,^54^ was a distinctive feature of the Kendrew–Wyman document. A tentative list of contributors and themes was provided in September 1967:

Crick – Project K (already written)

? – electron microscope

Longuet-Higgins – information in biology

Weisskopf and/or Bernardini – experience of CERN

Brenner – studies of complex organisms

Watson –US views of the proposal

Delbrück – European émigrés and the laboratory

Jacob or Monod – control mechanisms

Levinthal – possibilities of application of NMR to biological systems

? – large scale preparation of micro-organisms and biological materials

? Chantrenne - ? Maaløe - ? E. Katchalski - ? Cohen - ? Liquori - ? Eigen - the attitudes of biologists in selected countries.

The rationale behind this list of appendices was evident: on the one hand, the Kendrew–Wyman document was primarily aimed at government officials, thus setting aside the more technical aspects of the laboratory’s research program (Brenner, Levinthal, Longuet-Higgins, Jacob/Monod); on the other, it was conceived as a federative project, designed to gather scientific and political consensus. This was to be achieved through reference to the CERN model (Weisskopf/Bernardini), comparisons with the US context (Watson and Delbrück), and potential endorsements coming from several European countries (West Germany, Italy, Belgium, the Netherlands, Denmark) and Israel.

On this basis, the meeting of the extended EMBO Laboratory Committee was held at the Faculty of Sciences, University of Nice, on September 23 and 24, 1967.

The choice of Nice emerged in late July from a conversation between Kendrew and Tissières in Geneva, reflecting the increasing seriousness of the French bid to host the EMBO Laboratory. Since May 1966, as members of the French interministerial working group on molecular biology, Monod and Jacob had strongly advocated the establishment of the EMBO Laboratory in Nice as a means of fostering research in molecular biology and biochemistry at the new Faculty of Sciences of the local university. In their view, locating the European Laboratory on French soil would have been extremely important for the prestige (*rayonnement*) of their country and the development of French science. During the summer of 1967, Monod discussed the project with the mathematician Jean Dieudonné, then head of the Faculty of Sciences in Nice.^55^ The personal friendship between Jacob and Maurice Schumann, the newly appointed French Minister of Scientific Research, also seemed to facilitate the prospects for establishing the EMBO Laboratory in France.^56^

In response to the French initiative, Great Britain—specifically the CSP Standing Committee on International Scientific Relations—prepared a diplomatic countermeasure. This involved two moves. The first was postponing a decision on the EMBO Laboratory until negotiations over the cost and site of the proposed new CERN accelerator (the 300 GeV Super Proton Synchrotron, SPS) had been resolved—an issue that had been on the agenda since 1964 (Krige 2003, p. 905) and ran in parallel with the EMBO Laboratory negotiations. The second was proposing an alternative to the centralized European laboratory in the form of a federation—the “split lab”—linking the MRC Laboratory of Molecular Biology in Cambridge, selected MPIs in West Germany, and the Pasteur Institute in France.^57^ This “scheme” was considered the best option to enable the UK to exploit its “scientific pre-eminence in the field,” and was seen as more in keeping with the CSP “general philosophy of building up centres by means of increased mobility of research.”^58^ Rather than creating a new intergovernmental organization dedicated to molecular biology, the CSP committee proposed pursuing limited bilateral agreements with EMBO, funding its fellowships through the European Science Exchange Programme (ESEP), established in 1967 by the Royal Society,^59^ and eventually “providing facilities for EMBO at Cambridge”^60^ as part of the “split lab” scheme.

Against the backdrop of this Franco–British competition, the Nice meeting played a key role in shaping the revised proposal for the EMBO Laboratory.^61^ At Nice, ten research groups and four services were defined, reflecting the vision of the EMBO Laboratory as a vast endeavor for the molecularization of life sciences: this included not only X-ray analysis and genetics, but also various biochemical approaches (protein chemistry, nucleic acid chemistry, physical chemistry, physical enzymology) as well as differentiation, membranes biology, immunology and neurophysiology. The Laboratory services were Computation, Production, Instrumentation, and Animal/plant houses.^62^ A preliminary list of the individuals to be asked to contribute appendices was also drafted. Additionally, the letter to émigré scientists was revised, and Wyman was tasked with delivering the final version.^63^

After the Nice meeting, time pressed: the EMBO Laboratory proposal had to be finalized by mid–December 1967 for pre-circulation among EMBC delegations before the end of the year. The final drafting resulted from an intense, collective effort, largely coordinated by Kendrew and Appleyard.^64^ It was essentially a Cambridge (Brenner, Crick, Huxley, Holmes, Klug)–Göttingen (the Eigen network)–Paris (Monod, Jacob) collaboration, resulting in an integrated combination of structural biology, molecular genetics, and biochemistry. Manfred Eigen and Günter Maass outlined the structure of a research group in biophysical chemistry, covering “all applied fields such as dynamical biochemistry, physical enzymology, physical chemistry of biopolymers (especially of nucleic acids) and also of lipid membranes.”^65^ Eigen’s collaborator, Leo De Maeyer, prepared the section on the service group for instrumentation and computation.^66^ Porter drafted the outline for a hypothetical immunology group.^67^ Brenner contributed details on the neurophysiology group,^68^ while Brian Hartley worked on the protein and nucleic acid chemistry research group, as well as the service group for sequence analysis and synthesis.^69^ The electron microscopy group was primarily designed by Cambridge biophysicist Hugh E. Huxley,^70^ while Kenneth Holmes—en route to the MPI for Medical Research in Heidelberg—provided input on X-ray analysis for protein structure.^71^ An outline for the membrane chemistry group emerged through collaboration with Aaron Klug.^72^

The appendices to the EMBO Laboratory proposal were assigned and received between October 1967 and January 1968. They varied in nature and scope. Some offered detailed descriptions of specific research groups proposed for the EMBO Laboratory. Monod’s appendix focused on the creation of a “central technical laboratory,” dedicated to the “production, fractionation and ultimate isolation of specific biological macromolecules.”^73^ Huxley proposed the establishment of a “Biological Applications Centre” at the EMBO Laboratory, where “new technical developments in electron microscope design could be tried out and adapted for use in the biological field.”^74^ A more detailed account of EMBO Laboratory’s training activities was initially prepared by Chantrenne and Tissières,^75^ and later revised by Buzzati-Traverso, drawing on his experience organizing advanced courses at the LIGB since 1963.^76^

Along the lines of Crick’s “Project K,” other appendices addressed conceptual and experimental problems which could be more effectively attacked in the context of a transnational and interdisciplinary laboratory. Brenner wrote on “differentiation, morphogenesis and the nervous system;”^77^ Israeli biophysicist Aharon Katchalsky proposed a research program on the structure and function of cellular covers and intracellular membranes;^78^ Christopher Longuet-Higgins, at the time professor of theoretical chemistry at Cambridge, explored “biological information processing” as a broad interdisciplinary subject.^79^ Similarly, Jacob and Jerne emphasized the need for concerted multidisciplinary efforts to address, respectively, the regulatory mechanisms in microorganisms and multicellular organisms,”^80^ or the “complete solution of the antibody problem.”^81^

Two other appendices adopted a more political tone, presenting the EMBO Laboratory as a promising remedy for the European “brain drain” to the United States. Their authors—Max Delbrück and Victor Weisskopf—epitomized the transatlantic networks of exchange that EMBO aimed to reorient. A German-born physicist who had emigrated to the US in 1937 to become a major figure in phage genetics at the California Institute of Technology, between 1961 and 1963 Delbrück had taken a leave from Caltech to promote the establishment of the publicly funded Institute of Genetics at the University of Cologne, the first institute of molecular biology in the West German academic system (Wenkel and Deichmann 2007). Not surprisingly then, Delbrück’s appendix offered the perspective of an “ex-European Molecular Biologist situated for twenty years at one of the Meccas of European and American postdoctoral pilgrims.” In his view, the EMBO Laboratory proposal was “eminently reasonable as a relatively cheap and highly effective device to stem the tide of the brain drain from Europe to the United States.” By attracting young, promising researchers in the early years after their PhDs, the international laboratory would increase intra-European mobility and help prevent the “alienation from Europe” of this cohort.^82^

Born in Vienna to Jewish parents, Victor Weisskopf had also emigrated to the US in 1937. During the Second World War, he had participated in the Manhattan Project and, in 1946, had joined the physics faculty at MIT. As CERN Director-General from 1961 to 1965, he had played a key role in the birth of EMBO in 1964 (Krige 2002; Cassata 2015). In his appendix to the EMBO Laboratory project, Weisskopf drew extensively on analogies with CERN. Far from draining resources from national academic systems, CERN was—he argued—a “productive intellectual center” that served as “the best breeding spot for professors for National institutions.” In the spirit of the “cultural European unity” he advocated, Weisskopf called for the creation of a “Biological European Institute” as part of a broader “European Graduate School of Science,” ideally located near CERN. Such an initiative—he concluded—could “only have an invigorating effect on each national university by raising the level of research, by providing a pool of potential candidates for jobs, and by reversing the brain drain.”^83^

At the beginning of November 1967, Kendrew, Wyman, Appleyard and Delauche spent three days in Cambridge to draft the new EMBO Laboratory proposal, which was sent for comments to all the members of the EMBO Council and the EMBO Laboratory Committee.^84^ Some of them offered their final editorial suggestions,^85^ others took the opportunity to share their perspectives on how the proposal was being received politically within their respective national contexts. The Max Planck scientists, namely Adolf Butenandt and Manfred Eigen, played a particularly effective role in gaining consensus for EMBO projects. As president of the Max Planck Society (MPG), Butenandt reported on a recent conversation with the German Minister for Scientific Research, Gerhard Stoltenberg, adding that, in his view, “the project won’t be rejected by German officials.”^86^ This position was confirmed in confidential talks between Appleyard and Hans Hilger Haunschild, head of the German EMBC delegation, in December 1967. According to Haunschild, the German Research Foundation (*Deutsche Forschungsgemeinschaft*, DFG) still opposed the EMBO Laboratory, fearing “a loss of national scientists.” Nevertheless, scientific opinion was increasingly “gathering around Eigen and Butenandt in its favor.”^87^ The debate in West Germany remained open and fluid, with another possibility even beginning to emerge: “He [Haunschild] is also advancing the question, why not for once in Germany rather than France?”^88^ At the same time, Eigen informed Wyman of British efforts to gain support not only in West Germany, but also among the French and Swedish circles, for the alternative “split lab” project.^89^ As of December 1967, in fact, the CSP Standing Committee on International Scientific Relations still supported the MRC proposal of “providing laboratory facilities for EMBO at Cambridge as part of a scheme involving, say, three laboratories which would be developed as EMBO centres as an alternative to a single large laboratory.”^90^

The EMBO Laboratory project was sent to governments in December 1967, just one month before the second meeting of the “preliminary” EMBC. At this stage, the aim of the EMBO Council was clearly not to obtain formal approval, but rather to initiate discussion of the proposal “as the first step in the real campaign.”^91^ Ultimately, the project was expensive and challenging. It envisaged a staff of 385 in the first year of operation, with 162 qualified scientists and engineers. This figure was expected to reach 257 out of 567 total staff after four years. Estimated annual running costs were between eight and nine million dollars (around $80 and $90 million today).

During the January 1968 EMBC meeting, the UK delegation did not counter–propose its “split lab” model. Instead, it outright rejected the entire project to establish a new intergovernmental institution—the EMBC—for funding EMBO projects (Cassata 2026, p. 212). The British delegates made it clear that their objections were “mainly scientific:” molecular biology was not, in their view, “a clearly identifiable continuing field” warranting intergovernmental funding.^92^ Rather than creating a permanent intergovernmental body, the UK delegation called for a simple and temporary arrangement “enabling governments to channel support to EMBO through appropriate national non-governmental organisations.”^93^

The UK position was sharply criticized by all meeting participants, leaving the British delegation in complete isolation.^94^ Despite the vigorous opposition coming from London, the establishment of the European Molecular Biology Conference was approved. In addition, the EMBC treaty left open a window for the future EMBO Laboratory as a “special project.”^95^ The proposed EMBO Laboratory project garnered strong support from France, Italy and Austria, all of which declared themselves prepared to submit offers to host the Laboratory.^96^ In each of these cases—Nice, Naples, Vienna—the establishment of the EMBO Laboratory was conceived as a way to strengthen national and local investments in the field of molecular biology.^97^ Nevertheless, the Conference did not recommend approval of the EMBO Laboratory document at this stage, instead requesting that representatives of the member states carefully examine its contents before taking any formal decision.^98^

In sum, this section has shown that the first detailed EMBO Laboratory project was drawn up not in 1963 but in 1967. It emerged from collaboration between the Cambridge and Pasteur groups together with the MPI for Biophysical Chemistry in Göttingen—and envisaged an integrated combination of structural biology (X-ray diffraction, electron microscopy, nuclear magnetic resonance), molecular genetics, and biochemistry, extended to encompass membrane chemistry, immunology, and neurophysiology. Although it was a collective effort of the EMBO Laboratory Committee, a decisive conceptual contribution came from Raymond K. Appleyard, who gave the project a decentralizing turn by envisaging a “service role” for the laboratory in support of national research institutions and universities. At the politico-diplomatic level, what was at stake in this phase was the establishment of the EMBC, that is, the creation of an intergovernmental structure which would guarantee funding for EMBO activities, including the laboratory. From this perspective, Gaullist France, at the time the most influential critic of the EC as a supranational entity, had a crucial role in supporting the establishment of the EMBC (and the EMBO Laboratory project) as a way to advance its domestic policy objectives, while the UK, whose applications for EC membership were vetoed by de Gaulle in 1963 and 1967, opposed the creation of the EMBC, including any commitment to an intergovernmentally funded EMBO Laboratory. Viewed from London, the defense of British national interests lay in fostering loose, non-governmental arrangements rather than new intergovernmental organizations focused on a research area in which they were already pre-eminent.

## On Lake Konstanz: Revising the EMBO Laboratory Proposal (1969–1970)

Hopes for the prompt signing of the EMBC Agreement (scheduled for May 1968) were soon dashed. The EMBC treaty was not signed until February 1969, more than a year after the EMBC meeting of January 1968 at which the EMBO Laboratory proposal had been discussed. During this delay, the political and diplomatic landscape changed significantly, affecting the subsequent development of the EMBO Laboratory project.

The first notable shift was the emergence of West Germany, and particularly of the Max Planck Society (MPG), as a driving force for the EMBC and the EMBO Laboratory. From the outset, EMBO’s program of addressing the “American challenge,”^99^ and reversing Europe’s purported brain drain was closely aligned with the interests and priorities of the MPG, which had long supported interdisciplinary research in biochemistry, physical chemistry and biophysics (Schwerin and Kraft 2024). Two MPG biochemists were among EMBO’s founders in 1964: Adolf Butenandt, director of the MPI for Biochemistry of Munich and president of the MPG from 1960 to 1972, and Hans Friedrich-Freksa, head of the Department for Physical Biology at the MPI for Virus Research in Tübingen. Manfred Eigen joined the EMBO Council in 1968 and became its chairman the following year. Thanks to the efforts of Friedrich-Freksa and Alfred Gierer, director of the Department for Molecular Biology at the MPI for Virus Research, between 1965 and 1968 the Volkswagen Foundation—established in 1961 following the privatization of the car company—supported EMBO’s early development with a three-year start-up grant of DM 2,748,000, roughly the equivalent of $6.6 million today. Inspired by a phone call from émigré physicist-turned-molecular biologist Leo Szilard after the Ravello meeting,^100^ the Volkswagen Foundation’s grant extended to the international level a policy it had already initiated a few years earlier, aimed at introducing new “physical modes of thought” into West German biology (Rheinberger 2002). The generous grant proved essential in funding EMBO fellowships, workshops and courses, as well as covering the preliminary studies for the EMBO Laboratory.

Following the expiry of the Volkswagen Foundation’s grant, the establishment of the EMBC in 1968–69 as an intergovernmental organization, funded by member states in proportion to their GDPs, underscored West Germany’s leading role as first contributor, covering over 22% of the total cost. It is therefore unsurprising that delays in the signing of the EMBC treaty were driven by Bonn’s insistence that German be included as a third official language alongside English and French.

The founding of the EMBC coincided with the consolidation of German research policy through the formalization of the “Alliance of the Scientific Organizations,” as well as with the growing prominence of the MPG, under Butenandt’s presidency, in the definition of West Germany’s science foreign policy (Trischler 2024, 482–483; Sachse 2025, pp. 71–74). This also had an impact on the discussions concerning the EMBO Laboratory. In March 1968, in fact, at a meeting of the German Scientific Policy Advisory Committee, Butenandt suggested that West Germany offer to host the EMBO Laboratory in Munich, where the MPG was planning the establishment of a broad biochemistry campus in the village of Martinsried. Butenandt’s proposal gained significant traction, leading to the appointment of a subcommittee “to consider whether it would be better to set up a European Laboratory or to provide EMBO with the means for intensified collaboration between existing European molecular biology centers.”^101^ In September, after intense discussion, the German ad hoc Committee on European Collaboration in Molecular Biology unanimously endorsed the project of “a central laboratory with strong links to the national Research Establishments,” suggesting “that a suitable site in Germany be offered.”^102^

Along with West Germany’s increasingly influential role, the UK also re-emerged as an active player in the EMBO/EMBC context. Still knocking at the doors of the EC after De Gaulle’s second veto of November 1967, the UK reconsidered its position with regard to the EMBC in order to prevent complete isolation. This time British participation in the EMBC was accepted, but only as a first step toward the establishment of a wider European Research Council, not limited to one single discipline. The UK’s Secretary of State for Education and Science, Patrick Gordon Walker, put forward this proposal in Paris in March 1968, during a OECD Ministerial Meeting on Science dominated by the discussion of the “technology gap” between Europe and the US. A few days later, the CSP Standing Committee on International Scientific Relations, while reaffirming that the EMBC “could lead to the fragmentation, rather than the integration, of science in Europe,” added that “political considerations favoured participation.”^103^ The CSP Committee in fact regarded the EMBC as “an interim step towards the establishment of a wider European scheme involving a European Research Council.” Faced with the difficulties of defining the scientific scope and the administrative structure of the envisaged European Research Council, the project in the end “ran into the sands” (Collins 2016, p. 218). Meanwhile, the UK had reluctantly agreed to become a member of the EMBC.

While West Germany was emerging as a key player and the UK was reconsidering its position within the EMBC, the collapse of the LIGB in Naples in February—part of a broader political and economic crisis in the Italian research system (Cassata 2013, pp. 297–346)—together with de Gaulle’s resignation in April 1969, profoundly undermined the positions of Italy and France in the competition for the EMBO Laboratory.

This temporarily opened a window for Switzerland to reconsider the possibility of hosting the laboratory in Geneva, close to CERN. This had always been—and long remained—the dream of certain EMBO scientists. The idea also received consistent support from CERN Director-Generals, first Weisskopf and then Bernard Gregory. The Swiss Political Department had set the issue aside until 1967 in order not to compromise the diplomatic action for the establishment of the EMBC. By the end of that year, debates over the extension of the CERN site into Franco-Swiss territory had raised the possibility that the EMBO Laboratory could be located in the Pays de Gex, on the French side of the border.^104^ In February 1969, Kendrew revived the idea of an EMBO Laboratory in Geneva, asking Weisskopf to mediate with Bern’s authorities. Weisskopf, in turn, not only immediately contacted the Swiss Department of Foreign Affairs but also wrote to Jacob, suggesting that France explore the possibility of offering a site in the French region adjacent to Geneva. Ultimately, neither Paris nor Bern pursued this option, particularly after it became clear, in 1970, that Geneva would host the new “SuperCERN,” the 300 GeV accelerator, on Franco-Swiss land.

Against this shifting backdrop, a few weeks after the signature of the EMBC Agreement, on March 16, 1969, the EMBO Laboratory Committee convened at Guildhall, Kendrew’s house in Linton, twelve miles south of Cambridge, to revise the EMBO Laboratory proposal in preparation for the EMBC meeting of June. In view of the shortage of time, the new chair of the Committee, the Danish microbiologist Ole Maaløe,^105^ suggested adding a cover letter to the EMBO Laboratory document “emphasizing the changing scenery.” According to Maaløe, a restricted EMBO working group had to be established “to assess the present position” of molecular biology in all European countries and “obtain information about facilities under construction or being planned.” It was necessary “to project the laboratory on to the map of European molecular biology five years hence. In order to convince people of the need for a unique EMBO Laboratory,” he argued, “the committee themselves must be sure of this.”^106^ Any revision of the EMBO Laboratory document had to be done between June 1969 and the next EMBC meetings in early 1970.

At Linton, the members of the EMBO Laboratory Committee discussed the issues that had emerged following the submission of the “Nice proposal.” The first point of tension concerned the relationship between the European perspective and differing national contexts and needs. On the one hand, the EMBO Laboratory needed “to be part of a pan-European movement,” and ultimately contributing to a “European Research Council;” on the other, care had to be taken to ensure that time spent at the Laboratory would not disadvantage researchers when they returned to “the career structures of their own countries.”^107^ A second, controversial point concerned the scientific program and the size of the EMBO Laboratory. A “balance” had to be found between the vision of the laboratory “as an avant garde research establishment” and that of “a multidisciplinary advanced university.” The difficult compromise between an elitist research laboratory and a broader center for postdoc training was also closely tied to questions of scale. The EMBO Laboratory had to be large enough “to be available to people from the smaller countries and to cover as large an area of the field as possible” but not so large as to hinder communication between the research groups and “exceed the number of people of the right calibre available.”^108^ Finally, the question of the site for the EMBO Laboratory was still open. While “scientific excellence” was the priority, the “amenities” of the site were “important factors in attracting good staff and their families.”^109^

In the end, the EMBO Laboratory Committee selected “three ideas” which had to orient the revision of the EMBO Laboratory document in order to “appeal to governments:”

- Applications of research to technology
- Will the laboratory aid the development of the university system of Europe
- Pan European ideas.^110^

At the end of the Linton meeting, a small group was tasked with providing information on “the present and future developments of molecular biology” in each EMBC country: Jaap A. Cohen and Arthur Rörsch for the Netherlands; Chantrenne for Belgium; Maaløe for Denmark; Giuseppe Bertani for Sweden; Brenner and Kendrew for the UK; the biochemist Hans G. Zachau for West Germany; Jacob (or Vittorio Luzzati) for France; Tissières for Switzerland; Hans Tuppy for Austria, and Buzzati-Traverso for Italy.^111^ Juan Antonio Subirana, for Spain, and Athanasios Evangelopoulos, for Greece, were added at a later stage.

Collected between April and May 1969, the reports confirmed that the general sentiment expressed at the Linton meeting—that “the mere existence of our laboratory project has stimulated development and planning of national laboratories along similar lines”—was not far from the truth. The most dynamic situation was observed in West Germany. In his report, Hans Zachau—who had laid the foundations of molecular genetics at the University of Cologne alongside Delbrück before moving to the University of Munich in 1967 to become professor of physiological chemistry^112^—identified three areas of particular relevance: Munich, with the creation of a new broader MPI for Biochemistry, planned for 1972, in Martinsried, a suburb of the city; Göttingen, home to the MPIs for Physical Chemistry and Experimental Medicine; and Tübingen, with the MPIs for Biology and Virology, as well as the Federal Research Center for Virus Diseases of Animals.^113^ In other national contexts—Sweden, Denmark, the Netherlands, Switzerland, Belgium, France, and the UK—developments were less dramatic but nonetheless pointed to the growing integration of molecular biology within university systems.^114^ Finally, a third group of reports highlighted the situation in countries such as Austria, Greece, and Spain, where molecular biology had not yet been sufficiently institutionalized. In all these contexts the future EMBO Laboratory was envisioned as a strategic tool to boost local developments.^115^ The only report missing was that from Italy, a telling sign of the collapse of the LIGB in Naples and, more broadly, of the disrupted state of the Italian research system (Cassata 2013, pp. 350–360).

After this in-depth survey of the state of molecular biology and its potential development in Western Europe, the EMBO Council and the EMBO Laboratory Committee reconvened in Geneva on June 1, 1969, just before the EMBC session. Both meetings agreed to ask the EMBC “to recommend that EMBO continue to study the possibilities of an international Laboratory.”^116^ The political context brought some positive news. First, Gerhard Stoltenberg appeared “very pro-lab” and it seemed possible that “the Germans” would “offer a site near Munich.”^117^ Second, thanks to the mediation of Weisskopf and Jacob, the Swiss and French governments were reconsidering the possibility of supporting an EMBO Laboratory located near Geneva:^118^ “My understanding—Kendrew wrote to Gregory in June 1969—is that the French Government is now prepared to consider a site immediately adjacent to CERN and to drop the idea of Nice. This seems to me a most admirable solution.”^119^

However, reactions from the scientific community were more divided, particularly in the UK and Sweden. At the EMBO Laboratory Committee meeting, Kendrew reported that Arne Engström had “put up a scheme for a Swedish Laboratory of Molecular Biology which seemed to be a direct competitor.”^120^ This Swedish initiative aligned with British advocacy for a “split laboratory” as an alternative to EMBO’s single European Laboratory concept.^121^ As a member of the CSP Working Party on the Proposed European Laboratory for Molecular Biology—established in December 1968—Kendrew had observed growing interest in the “split lab” model.^122^ In its first two meetings, held in February and March 1969, the Working Party had weighed the options of a “single European Laboratory” versus the “Federation of Laboratories,” favoring the “federal grouping of smaller European laboratories”—potentially in France, Germany, and UK (with “a Scandinavian site likely to be acceptable to UK as an alternative to one in the UK itself”)^123^—as the most effective way of fostering fruitful interaction with national universities. By contrast, the idea of a single European Laboratory for molecular biology was considered too costly and unjustifiable without reference to the “general development of the European scientific community.”^124^ For this reason, the Working Party had carefully assessed comparisons with CERN, reaching the conclusion that the CERN analogy was not an appropriate argument in favor of a single laboratory model for molecular biology.^125^

Not surprisingly the idea of a “split lab” resurfaced in April 1969, during a meeting in London between a CSP delegation and representatives of the German Council for Science and the Arts (*Wissenschaftsrat*). While all members of the CSP delegation—except Kendrew—supported the “multiple laboratory” model, the *Wissenschaftsrat* representatives firmly advocated for “the formation of a central international laboratory for molecular biology” as the only way to provide “an imaginative stimulus particularly to the young men going abroad to be trained in it.”^126^

At its meeting of June 1969, the EMBO Council formally rejected the idea of the “split lab,” while defending the concept of the EMBO Laboratory as an international, unified “meeting place.” Moreover, it accepted Brenner’s proposal to organize a brain-storming meeting with the aim of improving and redefining the scientific program of the EMBO Laboratory.^127^ In Geneva, the EMBO Laboratory Committee decided to arrange this “high level group” meeting—“15 to 20 very bright people” for “a 2- or 3- day brainstorming”—at the end of November 1969, during the Thanksgiving weekend, to facilitate the participation of the US colleagues.^128^

The EMBC meeting, held on June 2–3, authorized EMBO to “continue its studies on the proposed laboratory” and allocated 12,000 EAU ($12,000) for this task.^129^ Only the British delegation submitted a critical note, prepared by the CSP Working Party on the Proposed European Laboratory for Molecular Biology, which described EMBO’s “assertions” as “mainly subjective,” urging the EMBC to carefully consider a set of broader issues: the overall objectives and priorities for Europe as a whole in fundamental biology; the eventual forms of joint action; the relative merits of these forms, “with particular reference to the creation of a single large laboratory;” and the implications of such a project “for other fields of science and for the development of science as a whole.”^130^

Significantly, the British Department of Education and Science (DES) had anticipated the content of this note by circulating a draft on May 20 to the science policy advisors of several EMBC member states (Denmark, France, West Germany, Italy, the Netherlands, Norway, Sweden, Switzerland). In the circular letter accompanying the draft, the DES deputy under-secretary John Francis (Jack) Embling openly shared the reservations of scientific councils in Britain about the eventual negative impact of the EMBO project on the national university system.^131^ The aim of the UK delegation was therefore to call on EMBO to “put their arguments on a more factual basis.” Moreover, as the issue of the EMBO Laboratory was seen as affecting “science policy as a whole,” EMBC governments were urged not to confine discussion to “a forum devoted to questions of molecular biology alone.”^132^

British political and scientific criticism of the EMBO Laboratory proposal re-emerged a few months later, in October, during a special meeting convened by the Royal Society to debate the “arguments in favour of, and those against, greater participation in its proposed laboratory or laboratories.”^133^

Background papers for the meeting were provided by the director of the Imperial Cancer Research Fund, Michael G. P. Stoker, and by Kendrew. Stoker summarized the main issues discussed by the CSP Working Group on the Proposed European Laboratory for Molecular Biology. While acknowledging that “political arguments” for UK support of the EMBO Laboratory could “substantially influence the ultimate decision,”^134^ he nonetheless stressed the “lack of objective evidence” for the EMBO proposal,^135^ and argued instead for the advantages of the “split lab” model.^136^ In contrast, Kendrew reaffirmed the arguments in favor of the EMBO Laboratory, focusing especially on its federative elements “as a natural focus for European biologists” in both research and postgraduate training, comparable to institutions such as Woods Hole and Cold Spring Harbor.^137^ Opening the discussion and speaking “as an individual and not as an official representative of EMBO,”^138^ Sydney Brenner reiterated once again that the EMBO Laboratory would not have drained resources from national universities but would have trained a “new generation of scientists,” spreading the molecular approach “more widely in University biological departments.”^139^ Minutes of the general discussion at the Royal Society meeting were not recorded. However, the debate was reported in *Nature* and *Scientific Research*, which documented the attack on the EMBO Laboratory project orchestrated in particular by cell biologist Henry Harris from Oxford University and biochemist Hans Leo Kornberg from Leicester University.^140^

While Kendrew and Brenner were busy defending the Laboratory project from the attacks of the Royal Society, EMBO was involved in organizing the brainstorming meeting for the revision of the Laboratory scientific program.

**Fig. 2:**
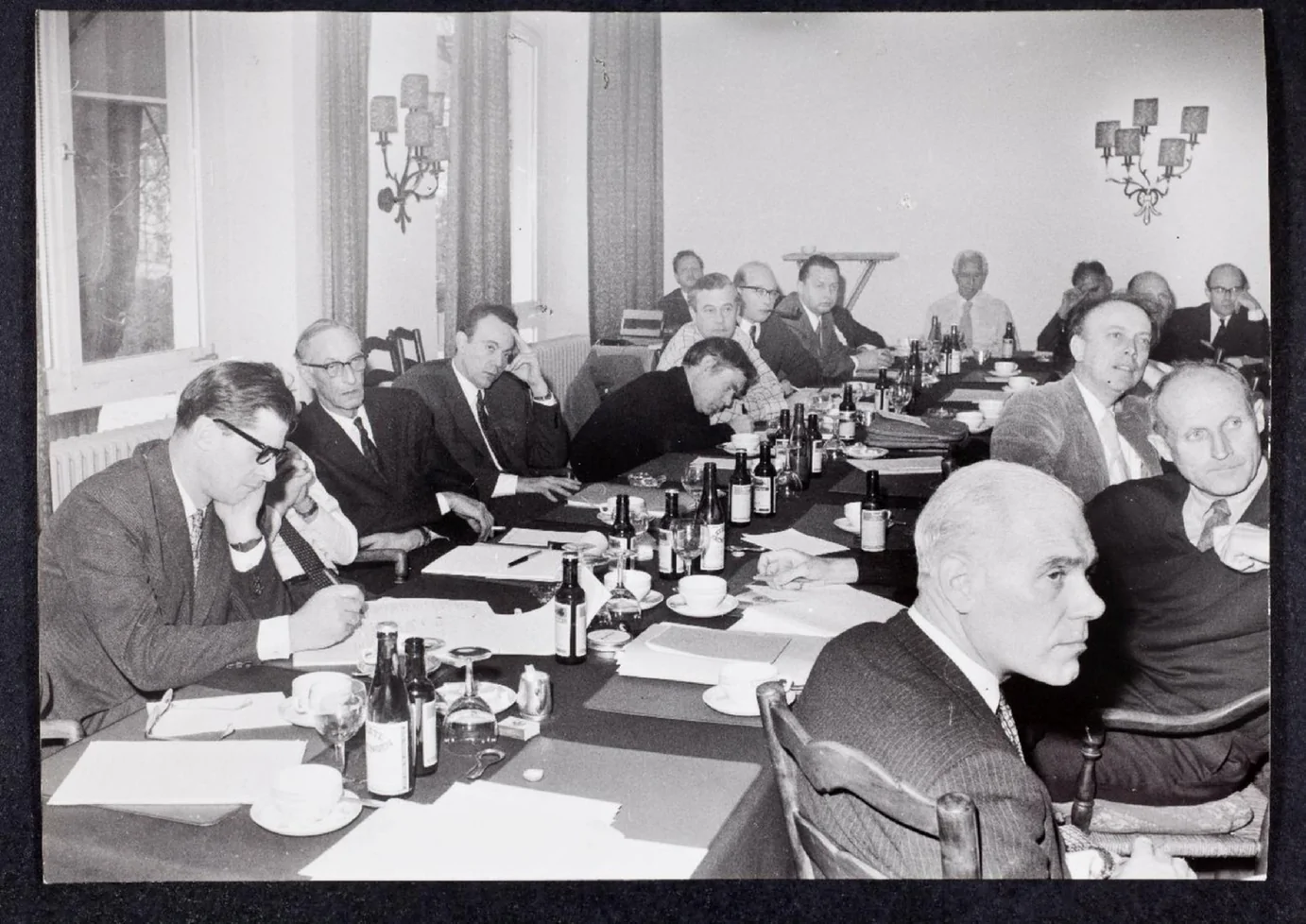
The “Konstanz” meeting. Left to right clockwise around the table: Stoker (uncertain), Ephrussi, Jacob, Brenner, unidentified, Zachau, Wittmann, Wyman, unidentified, Perutz, Watson, Tissières, unidentified. Courtesy of the Sydney Brenner Collection, BGI Nobel Laureates Archives, Cold Spring Harbor Laboratory, New York

The meeting was held at the Insel Hotel, a former monastery on Lake Konstanz, converted into a modern hotel, between 27 and 30 November 1969. There were forty-two participants, twenty of whom were members of the EMBO Council and EMBO Laboratory Committee.^141^ The remaining twenty-two were invited, mostly from the UK (nine), the US (four), West Germany (three), and France (three).^142^ Kendrew’s and Brenner’s influence on the selection of the participants was evident in strengthening the emphasis respectively on structural biology (Burgen, Klug, Phillips) and neurobiology (Changeux, Eccles, Jouvet, Hubel, Levinthal, Marr, Miledi, Reichardt (Fig. 2).

The Konstanz meeting was of crucial importance for several reasons. First, it was a future–oriented scientific event: a three-day brainstorming of “very bright people” discussing—in Brenner’s words—“the next 25 years of molecular biology.”^143^ As a dedicated occasion for revising the EMBO Laboratory proposal, it established a methodological precedent. The term “Konstanz” subsequently came to designate any EMBO future–oriented discussion aimed at revising the Laboratory research program. A similar occasion at the Hotel Haarlass, in Heidelberg, in November 1972, was referred to as the “second Konstanz meeting.”^144^ Second, it was a transatlantic meeting, scheduled during Thanksgiving to enable the participation of molecular biologists from the US, thereby reinforcing EMBO’s political self-representation as a European tool for reversing the “brain drain.” Third, it facilitated a process of epistemic mainstreaming in which Cambridge structural biology blended with the MPG’s more biochemically oriented approaches through a shared emphasis on expensive high-tech apparatus (X-ray crystallography, electron microscopy, spectroscopy, etc.).^145^

The first day of the Konstanz meeting, November 27, was devoted to a general discussion on the EMBO Laboratory project. In his introduction, Kendrew emphasized the “urgent need” to revise the 1967 proposal and update it. He argued that it was “vital” to persuade the “three major countries”—France, West Germany and the UK—to “cooperate in promoting” the new project. “Politically speaking,” he observed, West Germany was “the more enthusiastic,” while Britain was “the least.” On the question of the Laboratory’s site, offers had been made, “including ones from France and Italy,” but the “most appealing idea” remained to establish it “next to CERN in Geneva.”^146^ In his intervention, Victor Weisskopf endorsed this option, stressing once again not only the value of “an EMBO laboratory next to CERN” but also the close analogy between the proposed Laboratory and CERN itself. He pointed to the intellectual atmosphere, the growing mobility of European physicists, and the impact on the development of physics at the national levels: all these factors in the “success of CERN”—he argued—could readily be translated into the field of molecular biology.^147^

Weisskopf’s CERN analogy shifted the discussion to a central issue: the eventual size of the EMBO Laboratory and its relationship to the scientific program. Many participants, including Crick, Perutz, and Watson, argued for the need to scale it down in order to foster exchange among researchers and avoid “a great amount of mediocrity and inertia.”^148^ It was Brenner, however, who offered the most effective suggestion to resolve what he saw as an inherent contradiction in the 1967 EMBO Laboratory document, that is its oscillation “between cheapness and ‘Cernization,’ i.e. vast expense.” He proposed to give priority to “a nucleus for the laboratory,” around which the rest of the project could gradually develop: “The present Laboratory project would die while still under discussion—Brenner warned—if nothing were done to implement it.” This nucleus—“3–5 groups of research workers, totaling 30–50 in all”—was to be recruited largely “from Europeans now in America” and supported initially by a single country for a limited period.^149^ The EMBO Laboratory’s subsequent development would depend on the quality of this nucleus and the direction of its early work. Brenner also insisted that the “final objective” of the EMBO Laboratory needed to be redefined. The original claim of the 1967 document—that molecular biology was poorly supported in Europe—could “no longer be said to be true.” Instead, “maximum importance” should now be placed “on the providing of facilities, which could not be provided by the university, for young people to do productive research in the five most important years of their scientific career.”^150^

Brenner’s energetic and pragmatic intervention had a strong impact on the discussion that followed. Most participants accepted his suggestion, while rejecting the idea of a unidirectional brain drain from the United States to Europe. They proposed instead that EMBC governments be asked to provide job assurances—“a post in a university or national laboratory”—for young scientists who accepted temporary appointments at the EMBO Laboratory.^151^

The remainder of the Konstanz meeting focused on revising the scientific program. Over two days of intensive discussion (November 28 and 29), the participants explored the frontiers of molecularization in the life sciences. Developmental biology (Jacob, Brenner), structural biology of complex systems, in particular membranes and ribosomes (Burgen, A. Katchalsky, Klug, Phillips), neurobiology (Marr, Reichardt), and immunology (Klein) were among the most discussed topics. Computation and advanced instrumentation were constantly referred to.^152^ When François Jacob presented his “megamouse project”—a vast research program for the study of development based on the mouse as a new model organism—he reframed it as the “big machine” that the EMBO Laboratory could “sell” to EMBC governments.^153^

Sunday (November 30) was devoted to a meeting of the EMBO Laboratory Committee, with the aim of developing a strategy to restructure the project by January 1970. The Committee identified the “basic error” of the 1967 proposal as lying in “its completeness.”^154^ Following Brenner’s suggestion, it was agreed that the new proposal should encompass “a wide area” but be articulated in two stages, with an “initial nucleus” of around sixty scientists, eventually followed by a successive expansion, “depending exclusively on scientific requirements.”^155^ The subjects proposed for inclusion in the revised project were “instrumentation, cell biology, structure studies of complex systems, membranes, and the mouse project (to include neurobiology).”^156^ Working groups were established to define the new program, focusing respectively on subcellular structures, cell genetics, and instrumentation/computation.^157^ The importance of postgraduate teaching, and the potential for involving undergraduate students “for part of their degree course,” were identified as persuasive arguments “for convincing governments,” and were to be emphasized in the revised proposal.^158^ To begin defining the “initial nucleus,” lists were drawn up of scholars “who might come to work at an eventual EMBO Laboratory” in the fields of “macromolecular assembles,” “membranes,” “cell biology,” “neurobiology,” and “protein and nucleic acids.”^159^ Regarding the site of the Laboratory, the Committee agreed that the option of locating it “next to CERN” should be mentioned, though “only as a possibility, without any commitment whatsoever.”^160^

The revised proposal for the EMBO Laboratory was drafted in a couple of months, between December 1969 and February 1970. Referring in its introduction to the Konstanz meeting, the new project placed “much emphasis on instrumentation, as well as on a strong programme of biological research conducted in close collaboration with this.”^161^ The focus on advanced instrumentation reinforced the CERN analogy, establishing a direct connection between international concentration of resources, interdisciplinary approach, and the kind of technological innovation that EMBO considered fundamental for the advancement of molecular biology. Moreover, the new project not only made the EMBO Laboratory more specialized in instrumentation development but also dramatically scaled down its size, to “between one quarter and one fifth the size envisaged in the previous plan.”^162^ Unlike the 1967 proposal, the new EMBO Laboratory was not presented as a self-sufficient institution. On the contrary, its success depended on being placed in an appropriate technological and industrial environment, in close connection with a large research center in which sophisticated instrumentation and computation were being developed for other purposes. Once again, the first option appeared to be CERN.

In terms of scientific staff, the total number of permanent positions was rather small (around eight). With what was undoubtedly an excess of optimism, the new proposal stated that a “number of first-rate molecular biologists, among them some Europeans now working in America,” had already expressed interest in joining the EMBO Laboratory. These arguments were clearly instrumental in endorsing the image of the new international laboratory both as a resource for “existing national institutes and universities” in Europe,^163^ and as a remedy for the brain drain to the United States.

The organization of the EMBO Laboratory was structured around two main divisions: the Biological Division and the Instrumentation Division. The former included two groups, focusing respectively on subcellular structures (membranes, ribosomes) and on cell genetics. These research activities—it was argued—demanded “radical developments in instrumentation, technology, and computer software.”^164^ For this reason, the “unique contribution” of the EMBO Laboratory at the international level was identified as lying in the development of “radical and imaginative innovations in instrumentation and technology.”^165^

The latter was the focus of the Instrumentation Division. The research program was elaborated through the advice of Mervyn Hine and Kenneth Holmes: Hine was Director for Applied Physics at CERN and supervisor of CERN’s computer development; Holmes had recently moved from Cambridge to the MPI for Medical Research in Heidelberg and had contacted the German Electron Synchrotron (*Deutsches Elektronen–Synchrotron*, DESY) in Hamburg to inquire about the potential of synchrotrons as a source of high-intensity X-rays capable of generating higher resolution diffraction patterns for research in structural biology.^166^

With the aim of matching “the intellectual level of scientific development in the biological field with a comparable level of experimental technique,”^167^ the Instrumentation Division identified X-ray (or neutron) diffraction and electron microscopy as the two areas with “immediate priority.”^168^ Both projects were intended to provide “useful services” for biologists across Europe.^169^ In electron microscopy, an interdisciplinary, “problem-oriented” group was deemed necessary to improve specimen preparation techniques for unconventional prototype instruments, such as the high-resolution scanning microscopes and microscopes equipped with phase-contrast or dark-field devices. With regard to X-ray diffraction, thanks to the contacts that Holmes had already established in Hamburg, the new project envisioned the creation of a molecular diffraction facility at the DESY storage ring as a “detached outstation” of the EMBO Laboratory.^170^

The notion of “outstation” was a significant conceptual and institutional innovation: on the one hand, it echoed the CERN model, reinforcing the dependence of the EMBO Laboratory on unique technological facilities in the Western European context; on the other, it paved the way to the decentralization of the EMBO Laboratory through bilateral agreements between the central laboratory and specific facilities located in other European national institutions, reducing intra-European competition. Plans for the incorporation of an “EMBO radiation bunker” into the DESY storage ring were already in place in the first months of 1970,^171^ and a more detailed project for this outstation was added to the revised proposal for the EMBO Laboratory.^172^ A second outstation was proposed in 1971, when the Franco-German high-flux reactor became critical at the Institut Laue-Langevin (ILL) in Grenoble, providing an opportunity to establish an EMBO neutron diffraction facility there.^173^

In addition to the outstations, the revised proposal included two other projects that enhanced the federative functions of the EMBO Laboratory. The first concerned the role of the Laboratory as a center for pre- and post-doctoral training, “closely following the example of Cold Spring Harbor in the USA.”^174^ This educational dimension was particularly emphasized in the new proposal, responding to explicit requests from EMBC member states. For example, in December 1969, after talks with Jack Embling, Kendrew suggested “to lay particular stress on the teaching functions of the laboratory, on the analogy with Cold Spring Harbor, and on the possibility of post PhD training.”^175^

In addition to providing advanced teaching for postdoctoral fellows and for a small number of pre-doctoral students, the second federative feature of the proposal was more closely linked to advanced instrumentation. This involved allocating an area as a “project space,” available to short-term visiting scientists from national laboratories working either individually or in groups on projects of their own, where the EMBO Laboratory offered specific facilities.^176^

Although envisaged as “a rather small laboratory of definite size, with a program of obvious scientific significance devised to be of maximum assistance to biological laboratories throughout Europe,”^177^ the EMBO Laboratory was also presented as a flexible “pilot enterprise,” from which “something much more ambitious and comprehensive could be developed.”^178^ The construction of new facilities for “biochemical engineering,” the establishment of a very large mouse colony, the creation of a high-intensity X-ray source within the EMBO Laboratory, and the development of advanced computer applications were identified as four major avenues for its future expansion and for consolidating its international role.^179^

Looking at possible sites, CERN was mentioned as the most natural neighbor for the EMBO Laboratory.^180^ However, other possibilities had to be carefully examined, not only with a view to the desirability of close contact with an existing center of advanced technology but also bearing in mind other desiderata such as “intellectual milieu, accessibility and availability of services including housing and schools.”^181^

Even before its submission to the EMBC, the revised proposal of the EMBO Laboratory gained the support of Great Britain.^182^ At the meeting of February 17, 1970, which significantly included Hugh Huxley among the participants in his role of chairman of the EMBO Working Group on Techniques and Instrumentation, the CSP Working Party on the Proposed European Laboratory for Molecular Biology accepted the new EMBO Laboratory proposal. At this stage, the CSP deemed the proposal justifiable because its research projects “seemed necessary and would be difficult to fund in UK,” because “suitable manpower would be more readily recruited,” and because “the effect on teaching would be more widespread.” The UK’s support for “the growth of European science” was considered desirable “even if some short-term financial restriction resulted,” and the scale of the proposal was now of “the right order,” making a strong case for a possible UK site of the Laboratory.^183^ Culham in Oxfordshire, where the laboratory of the UK Atomic Energy Authority (UKAEA) for fusion research was located, was suggested as a suitable option.

In early April, the first plenary EMBC meeting was held at CERN headquarters. On this occasion, with the partial exception of Sweden, all delegations endorsed with enthusiasm the newly revised EMBO Laboratory proposal. They supported EMBO’s suggestion to establish an EMBC Study Group on the EMBO Laboratory, composed of representatives from all member states and chaired by the President of the EMBC (and head of the Swiss delegation) Henri Voirier, which was to convene in autumn—before the next EMBC meeting of November 1970—with the aim of conducting a more thorough analysis of the Laboratory project. Particular attention had to be paid to “the important question of the relationship between the work on molecular biology being carried out at universities and other national institutions and the future European programme.”^184^ In response to this request, the EMBC also agreed to organize “a broad-level meeting of representatives from national institutions” to coincide with the EMBC Study Group meeting.^185^ These high-level EMBO/EMBC meetings were planned for the end of September 1970 and hosted by the Netherlands in the city of The Hague. The choice of this location had a powerful symbolic meaning. Just a few months earlier, in December 1969, the six EC heads of state or government had convened in what became known as “The Hague Summit,” a meeting that marked a “significant milestone” (Patel 2020, p. 11) in the process of European integration, with the agreement to deepen the EC through economic and monetary union, as well as to widen it—by reopening the question of British accession. The “spirit of The Hague” seemed to hang over the EMBO Laboratory project as well.^186^

In anticipation of the forthcoming EMBO/EMBC meetings of September, West Germany took the initiative. At the end of April, the German ad hoc Committee on European Collaboration in Molecular Biology endorsed the newly revised proposal.^187^ One month later, an interministerial meeting, convened at the Federal Ministry of Education and Science in Bonn, forged a new political and scientific consensus regarding West Germany’s bid to host the EMBO Laboratory, with Munich emerging as a possible location. The Standing Conference of Ministers of Education (KMK), the German Research Foundation (DFG), and the Max Planck Society (MPG) all expressed their general support for a German hosting proposal. The establishment of a “German center” in Munich was compared to the creation of CERN “but this time to Germany’s advantage.” Moreover, Manfred Eigen reported that John Kendrew “had expressed his willingness to go to Munich and had wondered why no such German offer had yet been made.”^188^

At the end of July, the MPG sent Eigen the final draft of the site proposal for an EMBO Laboratory in Martinsried, south of Munich.^189^ The proposed location was adjacent to the planned biochemical complex, where the MPIs for Biochemistry, Cell Chemistry, and Protein and Leather Research—respectively headed by Butenandt, Feodor Lynen (Nobel Prize for Chemistry in 1964) and Walter Hoppe—were expected to merge.^190^ The proposal also mentioned the possibility of an alternative location near Munich–Garching, home of the MPI for Plasma Physics, an even larger research facility (200 physicists, 800 engineers and technicians) including a *Rechenzentrum* (data center) equipped with an IBM 360/91, one of the most powerful computer installations available at that time. The proposal was politically robust, enjoying the support of all the major institutional actors within West Germany’s complex research system; economically advantageous, as the government offered both the land and the laboratory buildings while also financing the construction of the DESY outstation; and flexible with regard to the possible sites for the EMBO Laboratory. Timing was also essential. In February 1970 John Adams, the 300 GeV project director, had suggested that the accelerator be built on the existing CERN site on Franco-Swiss territory, thereby sharply reducing costs. This turning point strengthened Bonn’s claim to host the EMBO Laboratory while undermining any competing proposals from Switzerland or France.

On a summer trip to Germany, Wyman immediately seized the opportunity to visit Munich with Walter Hoppe and later sent his impressions to Kendrew. Munich was “quite appealing,” Wyman wrote, but a German site was likely to raise objections “at a national level from some other countries, and others at a personal level.”^191^ The reference was to possible resistance among Jewish colleagues, as Wyman made clear with a personal anecdote: “Gratzer, hearing that I was going on with Hoppe, said ‘I hope you don’t find one’ (I have the impression Gratzer is Jewish).”^192^ Gratzer had been born in Breslau, Germany (now Wrocław, Poland), into a Jewish family. The family had lived in a small town near Katowice before fleeing the Nazis, moving first to Czechoslovakia and then, in 1939, to England. Memories of the Second World War, the Nazi occupation, and the Holocaust remained vivid among many members of the EMBO Council. Some—such as Max Perutz—had been refugees from Nazi Germany; others—such as Charles Sadron—had been deported to Nazi concentration camps; still others, including Monod and Jacob, had fought Nazism as members of the armed Resistance. As Kendrew himself acknowledged in a letter to Rörsch, such “feelings” were not limited to Jewish scientists within EMBO circles:

It seems likely that the German offer will be for a site at Munich and for my part, and speaking quite personally, I think this site would be excellent in every respect save one, namely the feelings that many people have expressed about working in Germany. Of course these feelings are strongest among Jews, but I know that others who are not Jewish have them too.^193^

Anti-German sentiment was not completely unexpected.^194^ During the first postwar decade, the Nazi past and post-war attempts by prominent German scientists to deny or downplay it had significantly hindered the normalization of international scientific exchange (Deichmann 2001). Although EMBO counted Adolf Butenandt among its Council members from the outset despite his controversial Nazi past, the EMBO Council had already rejected in 1964 the possibility of using the Volkswagen Foundation’s grant to begin building an EMBO Laboratory in West Germany. At the time, this decision had been motivated both by the limited institutionalization of molecular biology at the national level and by the desire to preserve the European dimension of EMBO’s programs. Nevertheless the “national smell”^195^ emanating from West Germany was certainly a factor. As Perutz wrote in March 1964: “All members of Council are opposed to our committing ourselves to having CERB in one particular country at this stage, and all are doubtful *especially about having it in Germany*.”^196^ Although the context in 1970 was different—the Munich proposal had been conceived under the umbrella of the EMBC, and the EMBO fellowships had already strengthened cooperation with West Germany in molecular biology—the past refused to pass, at least for some EMBO members.

Over the summer 1970, Bonn launched a strong diplomatic action directed at all EMBC member states with the aim of securing their support for locating the EMBO Laboratory in West Germany.^197^ In particular, on July 21, 1970, the Federal Ministry of Education and Science sent a note to the British Foreign Office expressing its firm wish to have the EMBO Laboratory established in Germany, along with the hope that the UK would support this proposal and accept Kendrew as director. The British response made clear that the UK held “a similar position,” but did not address the suggestion regarding Kendrew.^198^ To clarify the issue, a meeting between British and German representatives was arranged for September 3, in preparation for the intergovernmental meeting at The Hague. In a letter to the CSP chairman, Frederick Dainton, Jack Embling clearly anticipated what was at stake: “The German hope is to persuade us to withdraw from any rival offer of a site.”^199^ After “the failure of the 300 GeV project”—that is, the failure of Bonn’s attempts to secure the new accelerator on a site it had offered (Krige 2003, p. 905)—West Germany wanted the EMBO Laboratory on its territory, as recognition of its role as the largest contributor to European international projects. Moreover, if Kendrew were to be the first director, it would have been impossible to locate the laboratory in the UK. According to Embling, no other country appeared inclined to offer a site: “The French did so at an earlier stage but have now, I gather, abandoned it, and the Germans have hinted that the French support their offer.”^200^ In any potential competition with Germany, British chances of success were very limited. First, the German economy was in better condition: “They seem to have plenty of money, whereas we are short and likely to be even shorter.”^201^ Second, Germany was less restrictive than the UK with regard to the privileges and immunities of international organizations. Finally, the Germans could provide an attractive site in Munich, while the British proposal for Culham was undermined by the imminent construction of a maximum-security prison nearby.

Embling’s worries turned out to be rather realistic. At the meeting of September 3, British and German representatives reiterated their respective arguments.^202^ The Germans maintained that their offer of a site was already “on the table” and that it was “good for German science.” Moreover, the “German public and parliament” needed something “to show in Germany for their involvement in, and contributions to, European scientific cooperation.”^203^ In turn, the UK representatives not only declared that they too needed “something to show” in their country, but also claimed that their “two-decade record of strength in molecular biology” justified their intention to extend their contribution at the European level. In addition, while Germany was already an EC member, the UK needed “a symbol” of its involvement in European science.^204^ The German reaction was that, as an EC founding member, Germany’s claim was stronger than that of a prospective member like the UK. In balancing European projects in the UK and Germany, the Germans argued that the Dragon Reactor of the European Nuclear Energy Agency (ENEA) at Winfrith, in Dorset, compensated for the ESRO Data Center at Darmstadt.^205^ Other factors reinforced Germany’s position in the negotiation: the German offer was supported by Austria, Belgium, and Italy; the Germans had a “Munich site” “in mind,” but other possibilities, such as Heidelberg or Göttingen, could also be considered. Finally, Germany had signed the United Nations Statute of Privileges and Immunities and was ready “to give whatever P & I were needed.”^206^

These diplomatic negotiations anticipated the EMBO/EMBC meetings at The Hague, held at the *Nederlands Congresgebouw* (now the World Forum) on 28 and 29 September 1970. Henri Voirier presided over the two meetings. Conceived as a series of “short talks by EMBO people about different aspects of the project followed by question and answer sessions,”^207^ the meeting with the representatives of the national research institutions, on the afternoon of September 28, proved instrumental in demonstrating the support of European scientific communities for the EMBO Laboratory.^208^ On this occasion, Maaløe, the chairman of the EMBO Laboratory Committee, presented the results of a written enquiry that EMBO had launched in August, addressing all its members to respond on the following question: “Would you expect a European laboratory, as described in the revised proposal, to contribute directly, indirectly or not at all to the research and teaching efforts in your own laboratory?”^209^ Only seventy-three of the 213 members reacted to the questionnaire, more than half coming from the UK (fourteen out of forty-two), France (thirteen out of thirty-nine), and West Germany (eleven out of thirty-four); only one member out of fifteen had responded from Italy, and only eight out of twenty from Sweden. As predictable, most of the participants deemed that the EMBO Laboratory could have a “direct” effect on research and an “indirect” one on teaching.^210^ The intergovernmental meeting confirmed these results. The “strongest reservations” came from the representatives of Greece, Sweden and Norway, “mainly as a result of competition between the international and the national projects.”^211^ In Italy “the situation was confused,” Voirier wrote in his report.^212^

**Fig. 3:**
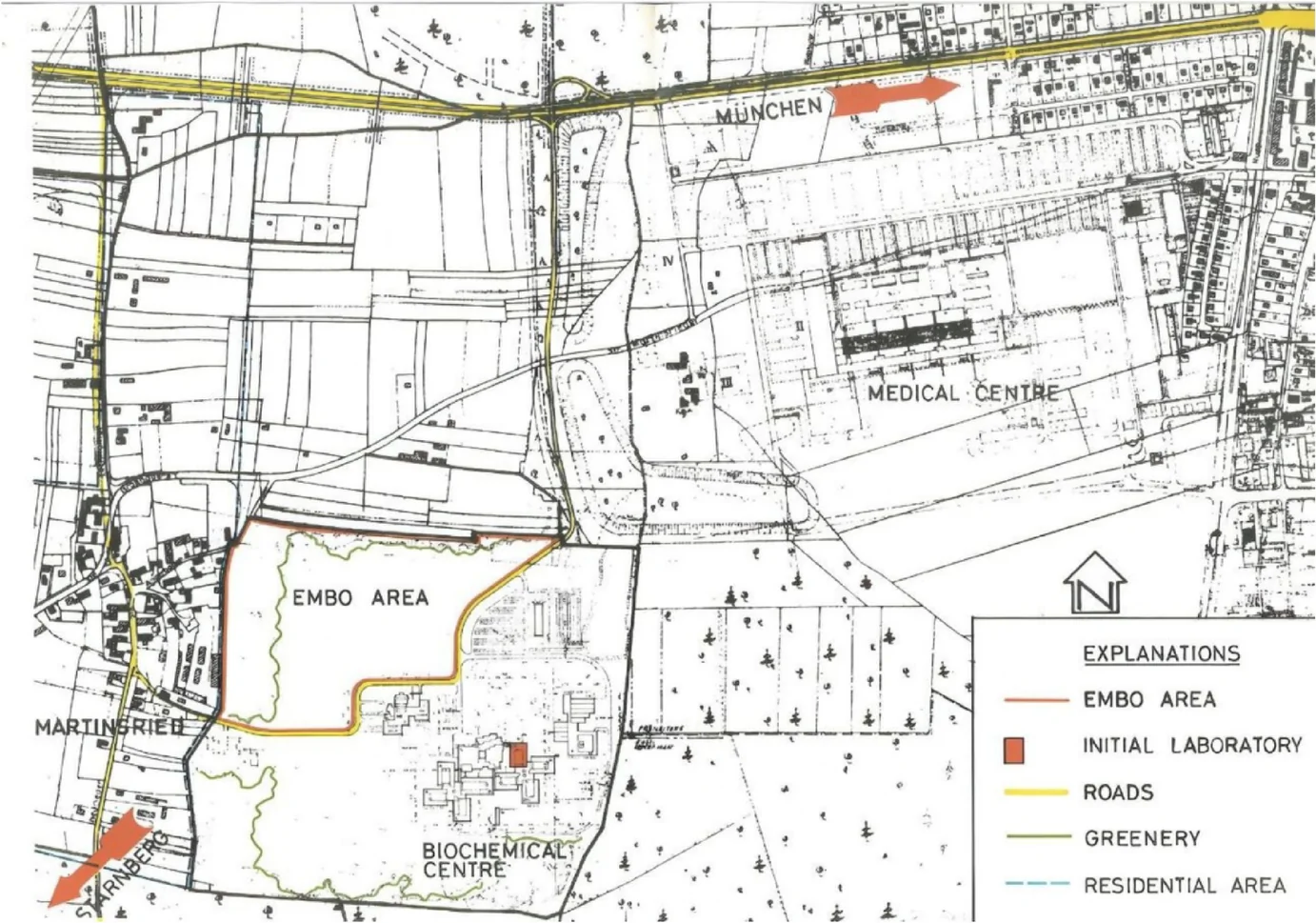
Note from the Delegation of the Federal Republic of Germany–Proposal for the location of a European Laboratory of Molecular Biology [1970] (CEBM/70/29). CC-BY

The following day, the meeting of the EMBC Study Group on the EMBO Laboratory marked the extent of the political consensus on the revised project.^213^ West Germany and the Netherlands were the most energetic in supporting the proposal, while the British delegates solicited EMBO to further clarify how the EMBO Laboratory could positively affect the state of molecular biology at the national level.^214^ Smaller countries—such as Greece—demanded forms of *juste retour* for their eventual investment in the project, going so far as to request that “instruments and apparatus made in the European Molecular Biology laboratory during a scientist’s visit, be brought back to his country at the end of the visit.”^215^ In the final resolution, all participants agreed to consider the EMBO Laboratory “scientifically desirable” and suggested EMBC to prepare “a detailed plan of action and development” and to set up jointly with EMBO “appropriate machinery to carry out the necessary detailed studies.”^216^ The German delegates formally submitted Martinsried as the candidate site, claiming to have the backing of the French Ministry of Foreign Affairs, for “reasons of political order (Fig. 3).”^217^

While approving the EMBO Laboratory in principle, France, the Netherlands and the UK preferred to await further details and the official EMBC session before endorsing the German proposal. The Italian delegation was absent, and this “defection” represented “a worry for EMBO.” Moreover, many members of the EMBO Council still regarded CERN, in Geneva, as the best location for the laboratory.^218^ Despite the uncertainty related to the Italian attitude, Voirier could optimistically report that European cooperation in the field of molecular biology was developing “harmoniously” and perspectives for the future were “encouraging.”^219^

To conclude, this section has shown how the EMBO Laboratory project was reconceived between 1969 and 1970 through a twofold yet convergent process of diplomatic negotiation and scientific revision. First, from the intergovernmental perspective, the formal establishment of the EMBC paved the way for the growing ascendancy of West Germany as the key diplomatic actor in the competition for the EMBO Laboratory. In addition, the resignation of de Gaulle in April 1969 and The Hague Summit in December 1969 gave fresh impetus to European integration at large. Second, from the scientific perspective, EMBO responded to sustained criticism coming from British and Scandinavian circles by producing a substantially revised proposal: smaller in scale, more focused on cell genetics and subcellular structures, and more explicitly federative in its institutional design. The CERN analogy was reinforced through a new emphasis on instrumentation and on the laboratory’s necessary proximity to a major physics research center, while new decentralizing mechanisms—the outstations, the “project space”—were incorporated to extend the laboratory’s appeal across a wider range of EMBC member states.

## “Les carottes sont cuites:” EMBO Laboratory’s Difficult Landing in Heidelberg (1970-1971)

Why Heidelberg, then, and not Munich?

In September 1970, the only formal offer of a site for the EMBO Laboratory was that of Munich, submitted by West Germany. Influential EMBO members such as Hans Friedrich-Freksa, Manfred Eigen and Hans Zachau had been carefully cultivating the Munich proposal since 1967.^220^ With Butenandt’s support, they had patiently navigated the intricate networks of the German research policy system, building regional and federal consensus around a project that integrated the envisioned EMBO Laboratory alongside the growing MPG programs in the Bavarian capital. Nevertheless, when the Munich proposal finally became a real and concrete option, it was not warmly received by the majority of the EMBO Council and the Laboratory Committee. Several factors help explain this cold reception. First, EMBO’s preferred option had always been Geneva. This enduring *esprit de Genève* was rooted in EMBO’s special relationship with CERN as a symbol of scientific internationalism and European cooperation. Second, after Konstanz, the revised EMBO Laboratory proposal placed particular emphasis on the role of Kenneth Holmes and his contacts in Hamburg, where the DESY outstation was to be established, and with the MPI for Medical Research in Heidelberg, where Holmes had moved from Cambridge. Finally, wartime wounds had not yet healed for some EMBO members, for whom Munich continued to be haunted by its past as the birthplace of the Nazi Party and epicenter of Hitler’s rise to power. These feelings were never openly expressed and were not recorded in any official document; however, they were occasionally hinted at in private correspondence among EMBO members or, as we will see, in confidential reports of the Swiss Department of Foreign Affairs, monitoring positions within EMBC and EMBO circles.

For all these reasons, even after West Germany energetically proposed to host the EMBO Laboratory, the path toward its establishment was far from straightforward, adding a further level of competition to the intergovernmental negotiations among EMBC member states—one that set divergent local projects and strategies against one another within EMBO. Despite these tensions, the joint EMBO/EMBC administrative structure provided a flexible institutional channel that helped balance EMBO scientific autonomy with the political goals of the EMBC member states.

At The Hague, on September 28, 1970, the EMBO Laboratory Committee established a Sites Committee to examine, first, the technical criteria to be met by any proposed site and, second, the extent to which the sites under consideration fulfilled these criteria. The Committee was chaired by Maaløe and included Hugh Huxley, Ken Holmes, and Charles Weissmann from ETH Zurich.^221^ Even in the earlier informal exchanges about criteria, different approaches emerged among members of the Sites Committee. The correspondence between Huxley and Holmes is particularly revealing in this regard. Drawing on the culture of the MRC Laboratory of Molecular Biology (de Chadarevian 2002, pp. 265–268), both shared the image of the canteen (or cafeteria) as a space of informal scientific interaction between the future EMBO Laboratory and an adjacent physics institute. But transposing this Cambridge model—at once symbolic and material—onto German soil was not straightforward. Much depended on the size of the physics neighbor. For Huxley, the EMBO Laboratory had to be located within walking distance of a major physics institute, employing some 300–400 qualified scientists and engineers, including “people involved in computing.”^222^ Holmes had a more nuanced position. By evoking the political and ideological climate of the early 1970s, he described the “big physics institute” as potentially being “an organization in the process of intellectual decline:”^223^

It would be desirable for EMBO to issue an overall statement of why fundamental biological research is useful at all i.e. are we doing fundamental medical research, is it only advanced play-therapy for neurotic scientists, or are we fulfilling on behalf of society a Freudian-like urge that society has for new knowledge. It really is necessary to make this justification to young scientists starting on their careers. They are very politically conscious and want to use their lives in the service of society if they can.

This point is especially important with respect to the large physics institutions. Since they find it very difficult to justify the role of fundamental particle physics with respect to the acute problems of present day society, a lot of good young people ran off and do [sic] sociology.^224^

Technological support—Holmes argued—could be obtained not only from large physics institutes but also from “the enormous and varied expertise locked up in European industry.”^225^ The “problem of computers”—he added—was not fundamental “since computers can be bought and, in any case, must be provided for growing user demand.”^226^ In addition to physics institutes, he suggested that universities could also serve as suitable neighbors of the EMBO Laboratory, provided they were “compact,” to preserve opportunities for close interaction. With these considerations in mind, the “best home for EMBO—Holmes wryly concluded—would be in Boston.” Among European universities, Oxford and Cambridge had “some attraction,” Munich was too “diffuse,” while Heidelberg was “compact” and showed promising developments in cell biology.^227^ Regarding large physics institutes, there were only three realistic options in West Germany: DESY in Hamburg, the MPI for Plasma Physics in Garching, and the Reactor Research Center in Karlsruhe.

These divergent views clearly conditioned the first round of visits by the EMBO Sites Committee. At the end of October, the group explored Martinsried and Garching in the Munich area, with mixed impressions.^228^ Some preferred to build the EMBO Laboratory near a “beginning institute” such as the Martinsried biochemistry complex, rather than risk being overshadowed by the MPI for Plasma Physics in Garching;^229^ others, such as Huxley, felt that only Garching could ensure the “close contacts with the physicists that would be necessary if the instrumentation projects were to prosper.”^230^ Clearly wary of EMBO’s reservations over the Munich proposal, Manfred Eigen informed the group that the German government “was willing to offer other sites in Germany.”^231^

The EMBO Sites Committee sought to overcome these tensions by providing the EMBC with a “memorandum on the criteria to be considered when selecting a site for the proposed EMBO Laboratory.”^232^

The document reasserted that the core of the “technological and intellectual milieu” envisaged for the EMBO Laboratory lay in its “immediate physical proximity” to large physics centers.^233^ The importance of “easy informal day-to-day contact” was justified on several grounds.^234^ First, a number of “technical problems” could be solved by directly asking the physicists next-door for specialized information and advice: “A great deal of time can be saved by talking to the right man over coffee in the cafeteria.”^235^ More generally, the interdisciplinary flow of information—whose historical relevance in molecular biology was recalled—could occur more effectively through “close personal contact” and in “an informal way.” Secondly, given the “continued extension of the use of computers in biology,” the interaction with a “large number of people” already involved in a “computer installation” was considered a crucial factor.^236^ Finally, from a more epistemological perspective, the “success of molecular biology in the last 25 years” was seen as demonstrating the fruitful influence of “a philosophy formerly prevalent among physicists,” namely “the optimism that unifying concepts can be found,” combined with “rigorousness” and experimental elegance.^237^

While close interaction with a large physics research institute was deemed “vital for the success of the laboratory,” contact with local biological centers was considered less important. On the contrary, being placed “immediately alongside an outstanding local centre of biological research” was seen as a potential threat to the scientific identity of the future EMBO Laboratory.^238^

The EMBO memorandum informed the deliberations of the EMBC Working Group III at its first meeting, in January 1971.^239^ Together with EMBO observers (Holmes, Huxley, and Luzzati), the Working Group included delegates from those states who had expressed interest, to varying degrees, in hosting the EMBO Laboratory: West Germany, Switzerland, Denmark, Italy, Austria, and Greece.^240^ On this occasion, the chair, Arthur Rörsch, listed a number of minimum requirements for the proposed official offer for a site. These included the proximity to a physics institute, “specialized in instrumentation” and staffed by people inclined to collaborate with biologists; a “good intellectual, cultural, and scientific environment,” in which, however, the new EMBO Laboratory could still “develop its own identity;” “good housing and additional facilities,” particularly foreign language schools, for scientists bringing their families; sufficient land (from three to ten hectares) for future extension; existence of local industries to provide services, material, specialized manpower, etc.; and good accessibility (“within one-hour drive from an international airport”).^241^ Following the EMBO Sites Committee memorandum and Rörsch’s suggestions, the EMBC Working Group III sent a detailed questionnaire to all EMBC member states, asking them to submit possible site proposals by mid-March.^242^

Between January 29 and February 3, 1971, a second working party of the EMBO ad hoc Laboratory Committee—comprising Maaløe, Holmes, Luzzati and the architect Jacques de Hoe^243^—visited West Germany and inspected not only the formally proposed Munich sites but also potential locations in Heidelberg, Karlsruhe, and Hamburg.^244^ The clear intention was to find alternatives to Munich.^245^ Holmes’s presence in Heidelberg proved crucial at this juncture. Two young researchers, Hermann Bujard and Peter von Sengbusch, who had recently arrived in Heidelberg from the United States,^246^ took the initiative, in collaboration with Holmes, to prepare a proposal for a Heidelberg site situated in the south of the city, in a hilly forested area, adjacent to the MPI for Nuclear Physics but also close to other important MPIs (Astronomy, Medical Research, Plant Genetics), the German Cancer Research Center, and the IBM Computer Center of the University of Heidelberg.

In contrast to the evaluation of October 1970, the EMBO group concluded that Heidelberg represented the strongest candidate. With respect to close liaison with a strong center of physics, while Martinsried had to be ruled out, there was “little to choose between the sites of Hamburg, Heidelberg, Karlsruhe and Garching.”^247^ However, the Heidelberg site benefited from the “very close geographic association” with the MPI for Nuclear Physics, allowing “better and more intimate contacts with neighbouring physicists.” The canteen, to be used by both groups, was “centrally located at easy walking distance.”^248^ Moreover, the MPI for Nuclear Physics had ongoing projects with the Karlsruhe Nuclear Research Center, which made the Heidelberg site “doubly strong.”^249^ Finally, the “general environment” was considered a “major argument” in favor of Heidelberg. The proposed site lay within walking distance from two modern housing developments under construction, each with adequate shopping centers and kindergartens; a new school, offering instruction in English, German and Russian, was also planned for the area. Both the housing centers and the laboratory site were situated on wooded hills just above the city. The “combination of natural scenic beauty of the immediate surroundings and the tradition and atmosphere of an ancient university town relatively uncontaminated by industry” was regarded as “an important factor in attracting scientists from outside Germany.”^250^ In contrast, the “immediate environment” of the other sites was described as “quite unappealing.”

The second EMBO report on sites threw the internal competition into sharp relief. The conflict clearly stemmed from different local strategies as well as divergent visions of the future identity of the EMBO Laboratory. Whereas Zachau prioritized the inclusion of the EMBO Laboratory in the broader MPG biochemical complex at Martinsried, Holmes favored the close day-to-day contact with the MPI for Nuclear Physics in Heidelberg and with other institutes of the local university. But other unspoken considerations were also at play. According to confidential reports by Swiss diplomats, who were monitoring the situation to assess a possible bid from Bern,^251^ many EMBO scientists regarded Munich as “the archetypal city of pure Germanic culture, closed to the outside world.”^252^ The reports further suggested that historical associations linked to Munich’s role in the rise of Nazism continued to shape perceptions, since “certain misgivings rooted in the past” were “less acutely felt in the case of Heidelberg.”^253^

When confronted with the second EMBO report on sites, Zachau did not hesitate to accuse Holmes of conflict of interest, disputing the technical details and insisting on the superiority of the Munich proposal in terms of “overall scientific potential,” “free time value” of the city, and the immediate availability of the lab space.^254^ Holmes rejected Zachau’s accusations and clarified his own position. In his view, DESY (Hamburg), IPP (Garching), and the MPI for Nuclear Physics in Heidelberg were all “excellent,” with not “much to choose between them.”^255^ The Karlsruhe Institute, with its 3000 employees, was “enormous” but also “rather attractive as a more distant neighbor.”^256^ Adopting what he called “the Huxley criteria,” Holmes argued that DESY was the best solution, followed by Garching and Heidelberg. However, the EMBO working party had decided that “the Huxley criteria” could be relaxed in favor of Heidelberg, taking into account that the MPI for Nuclear Physics “had good connections to the powerful physics faculty in the University of Heidelberg and to the reactor research centre at Karlsruhe.”^257^ The final report—Holmes noted—had been drafted in his absence and did not fully represent his “personal views.”^258^ In his opinion, the EMBO working party had several shortcomings itself: it was too small to be representative; the time was too short to gather all the necessary data; the list of sites was too restrictive: Göttingen, Stuttgart, Jülich, and Bonn should also have been “seriously considered.” For all these reasons, Holmes reaffirmed his good faith, refused to take part in further discussions involving Heidelberg and declined to serve again as a member of any EMBO sites working party.

The deadline for site proposals from EMBC member states expired on March 15 without the submission of any formal offer other than the German proposals for Martinsried and Garching. After visiting the two Munich sites, on March 17–19, the EMBC Working Group III was therefore obliged, in its report, to confront the “dilemma” of a choice limited to this area. In Martinsried the physics laboratories were located 30 km away, while the planned MPG biochemical complex represented a “‘threat’ to the identity” of the EMBO Laboratory. At Garching, by contrast, the facilities in physics were excellent. However, the EMBC Working Group expressed concern about the future large-scale development of the Technical University of Munich and of other projects in the area, which could eventually result in the EMBO Laboratory becoming “embedded in a huge national campus, albeit in a different field.”^259^ Moreover, the Garching site looked like a “wasteland.”^260^ While acknowledging that Heidelberg could be a competitive site according to EMBO’s own evaluations, the EMBC group could not discuss this option in the absence of a formal offer from the German government.

To overcome this impasse, Kendrew convened an “emergency meeting” of the EMBO Council on April 26 with the aim of reaching a “final EMBO opinion” on the relative merits of Martinsried, Garching, and Heidelberg. The German government—he argued—had decided that the site on offer would be “in Bavaria and nowhere else,” and this decision could be reversed only if the EMBO Council expressed “a very weighty opinion” in favor of a different site.^261^

A third EMBO Sites working party, chaired by Niels Jerne and including Kendrew, Günter Maass, Heinz-Günter Wittmann, John Cairns, and François Chapeville, visited Heidelberg on April 1.^262^ The group met the Heidelberg City authorities, together with the representatives of various University departments, of the German Cancer Institute, and of a number of MPIs. They obviously had lunch at the canteen of the MPI for Nuclear Physics. A few days later Jerne sent a “personal report” to Rörsch and to the members of the EMBO Council and EMBO Laboratory Committee.^263^

In terms of proximity with institutes of physics and biology, the third EMBO Sites working party restricted the choice to a preference between Garching and Heidelberg. Karlsruhe and Hamburg had to be ruled out because biological research was very limited there; on the other hand, Martinsried, Göttingen, Freiburg, Stuttgart were also to be excluded as sites in close proximity to strong biology research centers. According to the Jerne’s report, the MPI for Plasma Physics in Garching was “in no ways less strong” than the physics centers at Heidelberg—including the MPI for Nuclear Physics, the MPI of Astronomy, the University Faculty of Physics, the IBM development center, etc.—while the biological institutes in the neighborhood of Garching—namely the new Max Planck biochemical complex in Martinsried—were “as a whole, on a superior level” than those at Heidelberg.^264^ Nevertheless, “the diversity of efforts in Heidelberg, the emphasis on development and the smaller distances between the various institutes” made Heidelberg the best compromise.^265^ Heidelberg ranked higher than Garching also in terms of accessibility and attraction of the site. Although Munich was superior in schooling for international pupils and in all aspects of cultural life, Heidelberg had all the advantages of a small university town: it could be “compared to Oxford with respect to size and with respect to the importance of its university for the community.”^266^ Moreover, on the European map, while Munich, anchored to the Bavarian foothills, was oriented southward and eastward, Heidelberg sat in the Rhine rift valley at the meeting point of three natural corridors leading toward Amsterdam, Paris, and Basel: it was no coincidence that Jerne’s report explicitly referred to the good connections with Belgium and the Netherlands, France, and Switzerland. The housing situation looked attractive in Heidelberg as a satellite city (Emmertsgrund) was under construction on a beautiful site close to the one proposed for the EMBO Laboratory. For all these reasons, the third EMBO Sites Working Party concurred with the final recommendation of its predecessor in favor of the Heidelberg site.

Following Jerne’s report, the EMBO Council agreed that the case for Heidelberg seemed to be “a strong one, and that there was a *prima facie* case that it was at least as suitable as the two sites at Munich.”^267^ It was then decided to ask the German government to make an official offer, in order to organize other visits and prepare a proper recommendation for the next EMBC session.

At the end of May 1971, the Germans proposed a site in Heidelberg.^268^ As expected, the offer was based on the original draft elaborated by Holmes, Sengbusch, and Bujard.^269^

The EMBC Working Group III visited the site in July, exploring the local scientific institutions and walking along the *Philosophenweg* to the famous Castle overlooking the city.^270^ After further comparative analysis, the group recommended Heidelberg as the first choice among those offered by West Germany. Both Garching and Heidelberg satisfied the key requirement of having a strong adjacent physics institute, a condition lacking at Martinsried. However, the broader scientific environment was considered more favorable in Heidelberg. At Martinsried, the new MPG biochemical complex would have been much larger than the proposed EMBO Laboratory, while in Garching the EMBO Laboratory risked eventually being absorbed into a large national campus dominated by the Technical University of Munich and the MPI for Plasma Physics. Educational and other facilities for scientists and their families were judged to be roughly equivalent in Munich and Heidelberg.^271^

In the end, from a local perspective, it was Holmes’s victory. “Things are swinging quite literally in your direction,”^272^ Appleyard wrote in June 1971, and it inevitably caused some discontent. Zachau left the meeting of the EMBC Working Group III before its conclusion, leaving a few handwritten notes that once again emphasized the “bigger scientific potential” of Munich not only in biology, biochemistry, and chemistry but also in physics, provided—he added—that “the idea of a joint cafeteria with the physicists is dropped.”^273^ Temporary accommodation for the EMBO Laboratory—he wrote—was “excellent,” and Munich’s cultural life was “without parallel anywhere in Germany.”^274^ In July–August 1971, Hugh Huxley also protested against the choice of Heidelberg, claiming that an international laboratory based on advanced instrumentation would “fall to the ground” without proximity to a large physics institute,^275^ such as the one in Garching: “I believe—he wrote to Kendrew—the technical arguments should override a few people’s personal objection to Southern Germany, which I suspect is at the bottom of the objections to Garching.”^276^

The establishment of the EMBO Laboratory in Heidelberg was favored not only by the resolution of the dispute between the competing proposed sites in West Germany, but also by the parallel abandonment of alternative locations in the UK and Switzerland that had at various points been seriously considered.

In September 1971, the British government decided not to pursue Culham, in the Oxford-Harwell area, as a possible site any further. The news was not unexpected.^277^ If Kendrew was to become director of the EMBO Laboratory, as the EMBO Council had consistently suggested, it would have been impossible to locate the international laboratory in the UK. Moreover, West Germany had made it clear that it was prepared to offer “substantially more financial help to the international laboratory than any of the other 12 member governments.”^278^ Finally, although Culham was close to Oxford University and the Atomic Energy Research Establishment at Harwell, it would also have had an unwelcome neighbor: the maximum-security prison of Lockwood.

**Fig. 4:**
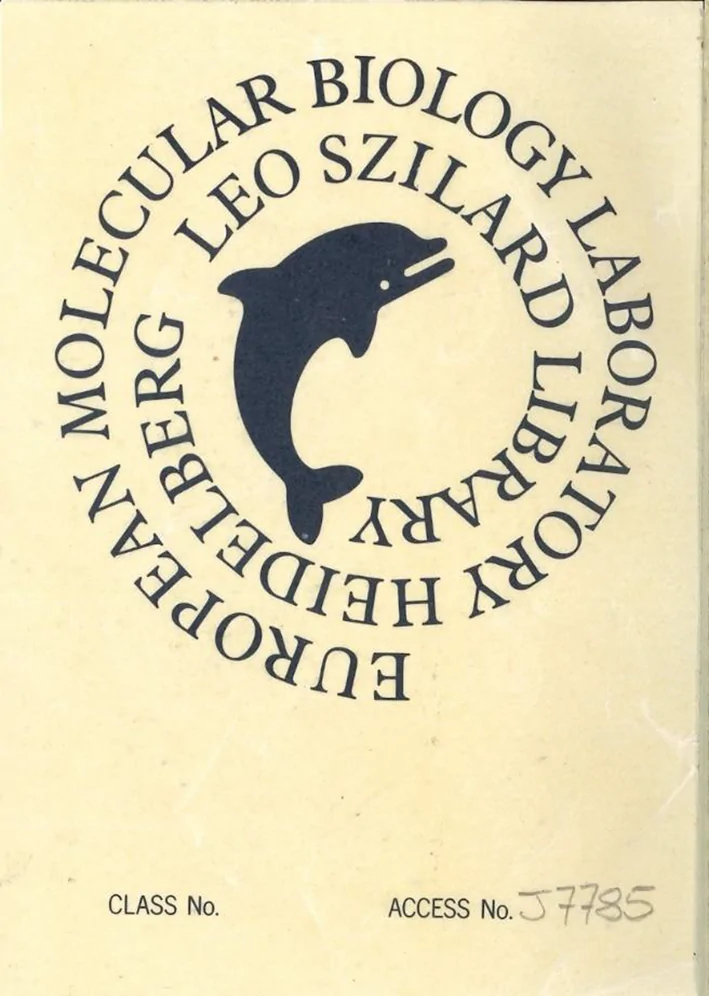
EMBL Library Bookplate, 1978, Library Promotional Material, DE 2324 SZI-A, BOX 369. CC-BY

Regarding the possibility of locating the EMBO Laboratory near CERN, as early as September 1970 Weisskopf had warned Kendrew that any attempt to establish the laboratory in Geneva would need to be postponed until March 1971, after the final decision on locating the 300 GeV SPS at CERN: “If the decision in positive,” Weisskopf wrote, “I would be rather skeptical about getting the EMBO lab to Geneva (I am sorry to say). Most people on the CERN Council are afraid of CERN getting too big. Also, the nations who did not get the machine are anxious to get something else, such as EMBO. In particular, Germany will fight hard to get EMBO, if CERN gets the machine.”^279^ The construction of the new accelerator at CERN was approved in February 1971. A few weeks later, Rörsch updated Kendrew about his conversation with the physicist Jan Bannier. Bannier, the vice-president of the Dutch organization for pure research (ZWO), as well as a member of CERN Council and chairman of the ESO Council, was rather dismissive about the prospects of the EMBO Laboratory eventually landing in Geneva at this stage: “According to Bannier—Rörsch wrote—there can be little doubt that nowadays the board of directors will not be very enthusiastic about an EMBO–annexe, because the building of the 300 GeV machine will keep them more than occupied.”^280^ Despite the pessimistic assessments of Weisskopf and Bannier, in May 1971 a small EMBO delegation—Jerne, Jacob, and Tissières—met with CERN Director-General Willibald Jentschke to explore the remaining possibility of establishing the EMBO Laboratory near CERN.^281^ Drawing on discussions with his colleagues, CERN scientific advisors, and the German EMBC delegate, Claus Zelle, Jentschke dashed EMBO’s hopes: CERN physicists “were not willing to come out with a unanimous statement in favour of EMBO in Geneva, until they were reassured that the political conditions were already solved, i.e. that the EMBO Conference was already willing to choose Geneva for the site of the laboratory and the Swiss government was prepared to go along.”^282^ Given these circumstances—Jerne wrote to Kendrew—it was “impossible to obtain a statement from the physicists at CERN to be used in order to affect the political negotiations,” and it was “pretty hopeless to pursue this matter further.”^283^ Jacob reached the same conclusion: “I think we had to do the trip to Geneva to meet with Jentschke, but I think this discussion was a decisive one in the sense that CERN can do nothing for us. They would be glad if we can manage to come there but by themselves they can not initiate any motion. I think therefore that ‘les carottes sont cuites’ and Heidelberg is the site.”^284^ The Swiss Department of Foreign Affairs soon adopted a similar position. In May 1971, when the German Foreign Minister and Vice-Chancellor Walter Scheel urged the Swiss government to renounce any potential bid to host the EMBO Laboratory, arguing that Switzerland had “already the SuperCERN,”^285^ Henri Voirier declined to provide an official response. “The SuperCERN was not a gift to be negotiated,” he remarked.^286^ Nevertheless, in an internal memorandum addressed to the head of the Swiss Department of Foreign Affairs, he encouraged the Swiss EMBC delegates to support the Heidelberg candidature. Concentrating the EMBO Laboratory and the “SuperCERN” in the Geneva region would have been politically inopportune and financially burdensome. Moreover, Voirier regarded West Germany’s ambitions as entirely legitimate.^287^

To sum up, this section has traced the siting process of the EMBO Laboratory as an integral part of the scientific and diplomatic negotiation that accompanied its establishment. Far from being exclusively the result of “the export of the ‘LMB culture’ to the European level” (de Chadarevian 2002, p. 332), or the natural outcome of a discipline constitutively defined as an international space (Abir-Am 1993), the choice of Heidelberg emerged from the complex interaction of three distinct sets of factors. First, it was conditioned by international competition among West Germany, Great Britain and Switzerland, each of which was simultaneously pursuing interests in both the EMBO Laboratory and the new CERN accelerator, and each of which ultimately had to weigh its claim to the EMBO Laboratory against its other interests in European scientific cooperation, and in European integration more broadly. Second, it reflected unresolved conflicts between competing research agendas within EMBO itself, notably between the Heidelberg group (Holmes), with its orientation toward structural biophysical studies, and the Munich group (Zachau), more interested in biochemical approaches and in the wider MPG complex in Martinsried. Finally, it was shaped by a comparative evaluation of the cities of Munich and Heidelberg that anchored the diplomatic negotiation to the material and cultural specificities of the place: the geographical position on the European map, the industrial landscape, the climate, the housing and schooling facilities for the families, and—less visibly—the unspoken postwar memory of the Nazi regime.^288^

## Conclusions

The EMBL Agreement was signed at CERN on May 10, 1973, by representatives of ten EMBC member states. In his speech at the ceremony, Kendrew proposed, for the first time, an origin myth for the history of the EMBO Laboratory: “As far as I was concerned it all began ten years and five months ago when I met in this building with the then Director-General of CERN, Professor Viki Weisskopf, with the distinguished ex–Hungarian American biologist, Leo Szilard, and with my own ex–pupil Dr Jim Watson, and there we discussed the idea of founding a European laboratory in molecular biology,” he wrote.^289^

The origin myth defined a date (December 1962), a place (CERN, Geneva), and a small group of founding fathers: Kendrew and Watson, arriving in Geneva following the Nobel Prize investiture ceremony in Stockholm, and two American physicists of European origin, the Austrian–born Weisskopf and the Hungarian–born Szilard, both involved in the Manhattan Project during the Second World War and later committed to nuclear non–proliferation.

A few years later, in 1978, following the inauguration of the EMBL building in Heidelberg, Kendrew’s origin myth was reinforced by naming the laboratory library after Szilard.^290^ A small dolphin swimming in a spiral, suggested by Szilard’s widow, Gertrude Weiss, and inspired by the logo of the Council for a Livable World, founded by Szilard as part of his crusade for nuclear arms control,^291^ was chosen to illustrate the library bookplate (Fig. 4). The EMBL dolphin visually connected Szilard to Kendrew: it evoked Szilard’s science fiction fable and political testament, *The Voice of the Dolphins*, while also referring to Kendrew’s research,^292^ which had made use of dolphins in his quest for myoglobin crystals suitable for X-ray analysis (de Chadarevian 2018).

As is often the case with foundational myths (Abir-Am 1999, p. 15), Kendrew’s recollection was factually inaccurate but symbolically powerful. Although very active in promoting the idea of an international laboratory in molecular biology around November–December 1962, Szilard was not present at the Geneva meeting.^293^ More than Szilard, Weisskopf had first suggested establishing an international laboratory of molecular biology patterned on CERN. *Before* December 1962, he had already taken concrete steps in this direction: in July, he contacted Appleyard, then head of EURATOM Biology Division, to discuss possible uses of CERN accelerators for biophysics research: “We ourselves, within the house,—Weisskopf said—are very attracted by the idea of having a biophysics neighbour;”^294^ in September, he also wrote to Buzzati-Traverso, welcoming the LIGB, recently founded by the Italian geneticist in Naples, as a potential realization of his own hope of having “an International Laboratory of Molecular Biology on a similar basis as CERN.”^295^

By placing Szilard and Watson (and, of course, himself) at center stage while expunging other relevant *European* actors (Appleyard, Buzzati-Traverso), Kendrew’s origin myth mobilized a powerful set of identitary boundaries. First, it defined a ten-year chronology linking the birth of an idea—allegedly stemming from an informal meeting of prestigious scientists at CERN in December 1962—to its successful realization with the signing of an international treaty in May 1973. Second, it cast the shadow of Cambridge (Kendrew, Watson) over the origins of the European laboratory, effacing its plural, conflictual, and European dimensions. Third, it reaffirmed—via Szilard and Weisskopf—the association of molecular biology with physics in terms of epistemological authority, science policy discourses, and Cold War imaginaries. Finally, and perhaps more importantly for the purposes of this article, it brought together the complex and partly conflicting models of CERN and Cold Spring Harbor into a single foundational narrative: Weisskopf had been CERN Director-General from 1961 to 1966; Watson had been director of the Cold Spring Harbor Laboratory since 1968; and the Cold Spring Harbor symposia had been crucial in Szilard’s post-war conversion to biology. Connecting the past to the future, Kendrew, in his speech at the 1973 ceremony, acknowledged the role of CERN—“our friendly host and counsellor ever since the beginning”—but described the EMBL as a provider of “services” for “national institutes” in Europe, evoking the presumed model of Cold Spring Harbor.^296^

Kendrew’s origin myth soon crystallized into a consolidated internal institutional history, repeated for decades (Tooze 1981, p. 1; States 1994, p. 5; Wassarman 2020, p. 198). From the 2000s onward, historiography has effectively deconstructed this foundational narrative, focusing in particular on the origins of EMBO (Krige 2002; Cassata 2015). By exploring the complex scientific and diplomatic process that led to the establishment of the EMBL, this article challenges many elements of Kendrew’s narrative while retaining his interpretation of the laboratory as a compromise between two different imagined models: CERN and Cold Spring Harbor.

In conclusion, three points merit emphasis. The first aim of this article was to clarify when and how the EMBO Laboratory was socially and politically constituted. EMBL did not emerge in 1962 from a meeting of world-renowned scientists in a room at CERN. It was the outcome of a complex social and diplomatic process, collectively driven by EMBO and EMBC, and significantly reframed between 1967 and 1970. Political negotiation and epistemic mainstreaming went hand in hand in forging a transnational laboratory for the molecular life sciences with a decentralized dimension (project space, outstations, services) and a scientific core centered on advanced instrumentation, especially oriented toward cell biology and subcellular structures.^297^

Secondly, this article has explored why the idea of a European laboratory of molecular biology, although it circulated widely in the early 1960s, gained decisive momentum only a decade later. Existing historiography offers two main explanations: the more favorable political climate of the early 1970s and the consolidation of molecular biology within national institutions, which reduced fears that a central European laboratory would siphon off domestic resources. This study complements that explanatory framework with a further argument. As shown in this article, national opposition to the EMBO Laboratory project remained strong in 1969–70, *despite* the increasing institutionalization of molecular biology within national academic systems. Resistance came not only from national research structures but also from alternative international—and more federative—configurations: the “split laboratory” distributed among three or four national laboratories; support for Europeanized centers of excellence; research projects carried out by groups of laboratories; or some form of European extension of the British MRC units. Why, then, did the *specific* solution of a central European laboratory ultimately prevail? Beyond the political and disciplinary factors already identified, this article has highlighted the relevance of institutional innovation. The EMBO Laboratory project cannot be separated from the new institutional architecture that emerged in 1967–73—the EMBO-EMBC-EMBL triad—which preserved EMBO’s autonomy, facilitated the framing of the project, eased political tensions, and provided new imaginative mechanisms for the Europeanization not only of molecular biology but of science more broadly.

Finally, this article has shown how the Europeanization of molecular biology culminated in a siting process involving two German cities: Munich and Heidelberg. Both sites were endorsed by a younger generation of German scientists (Zachau for Munich, Bujard and Sengbusch for Heidelberg) who had received part of their training in the US and sought international recognition. At the same time, within EMBO circles, both sites evoked memories of the past that still divided individuals of different ages, nationalities, and wartime experiences.

For some EMBO and EMBC members, Munich remained uncomfortably close to Dachau. By contrast, as Bill Slater, president of the EMBL Council, recalled at the inauguration of the laboratory building in May 1978,^298^ Heidelberg was the city where the biochemist Otto Meyerhof, recipient of the 1922 Nobel Prize in Physiology or Medicine, had been appointed director of the Kaiser Wilhelm Institute for Medical Research at the end of the 1920s. In 1938, facing increasing antisemitism, Meyerhof and his family fled Nazi Germany for Paris and later, in 1940, emigrated to the United States.

Meyerhof died in Philadelphia in 1951. Since the late 1970s, the EMBL address in Heidelberg has been Meyerhofstrasse 1.

## Data Availability

No datasets were generated or analysed during the current study.

## Footnotes

1 On the institutionalization of molecular biology in postwar Europe, see de Chadarevian (2002), Deichmann (2002), Gaudillière (2002), Santesmases (2002), Strasser (2006), Cassata (2013). For a comparative perspective, see Strasser (2002).

2 EMBO Council members came from very different scientific backgrounds: crystallography (Max F. Perutz and John C. Kendrew from the United Kingdom), microbiology (François Jacob from France and Ole Maaløe from Denmark), biochemistry (Hans Friedrich-Freksa and Adolf Butenandt from Germany; Arne Tiselius from Sweden; Jeffries Wyman from Italy), biophysics (Eduard Kellenberger from Switzerland, Charles Sadron from France and Arne Engström from Sweden), physical chemistry (Alfonso Maria Liquori from Italy and Ephraim Katchalski from Israel), embryology (Jean Brachet from Belgium), and genetics (Adriano Buzzati-Traverso from Italy).

3 These key agencies and institutes included the Medical Research Council (MRC) Laboratory of Molecular Biology in Cambridge (John Kendrew and Max Perutz); the Pasteur Institute (François Jacob); the Max Planck Institutes (MPIs) for Biochemistry in Munich and for Virus Research in Tübingen (Adolf Butenandt and Hans Friedrich-Freksa); the Institute of Molecular Biology at the University of Geneva (Eduard Kellenberger, Alfred Tissières); the International Laboratory of Genetics and Biophysics (LIGB) in Naples, headed by Adriano Buzzati-Traverso; the Swedish research institutes in Stockholm and Uppsala (Arne Engström and Arne Tiselius, the latter a 1948 Nobel Laureate in Chemistry for his research in electrophoresis); the group of the Free University in Brussels, coordinated by the embryologist Jean Brachet; and the Institute of Microbiology at the University of Copenhagen, directed by Ole Maaløe.

4 In the 1950s and 1960s, the Organization for the European Economic Co-operation (OEEC; from 1961 Organization for Economic Cooperation and Development, OECD, including also United States and Canada from the beginning), founded in 1948 to administer Marshall Plan aid, proved instrumental in transmitting the American ideational framework that connected scientific research to economic growth. According to Veera Mitzner, this specific framing of science policy, together with a long tradition of national narratives of “decline” and, somewhat paradoxically, the increasing wealth of Western European states, fueled European debates in the 1960s over the science “gap” and the “brain drain:” see Mitzner (2020, pp. 35–51). The phrase “brain drain” was originally coined in 1963 in the British context. It was first used by the London *Evening Standard* but is more commonly associated with the response of the British Minister of Science, Lord Hailsham, to a 1963 Royal Society report, entitled “The Emigration of Scientists” (Godwin et al. 2009). EMBO’s foundational documents reflected this debate: see EMBO (1966, p. 3). For the impact of these discussions on the institutionalization of molecular biology in Western Europe, see in particular Gaudillière (1996, pp. 422–423), de Chadarevian (2002, pp. 304–307), Cassata (2013, pp. 145–150), and Kraft et al. (2024, pp. 698–699).

5 I draw on the category of “technoscientific Europeanism” from Skogerboe (2025).

6 See EMBO (1966). For draft versions of the “EMBO Policy document,” revised by Kendrew and Wyman, see Kendrew Papers, Bodleian Library, Special Collections and Western Manuscripts (hereafter KP), MSS.Eng.c.2421.

7 I refer here to Hirschhausen and Patel’s interpretation of Europeanization as “a variety of political, social, economic and cultural processes that promote (or modify) a sustainable strengthening of intra-European connections and similarities through acts of emulation, exchange and entanglement and that have been experienced and labelled as ‘European’ in the course of history” (Hirschhausen and Patel 2010, p. 2). For a recent historiographical discussion of “Europeanization” as a category, with particular reference to science and technology, see Irion and Volf (2024).

8 Two “preliminary” sessions of the EMBC, respectively in April 1967 and January 1968, prepared the formal establishment of the European Molecular Biology Conference (EMBC). The Agreement was signed in Geneva on February 13, 1969. Following its ratification by the required number of European governments, the treaty officially came into force in April 1970.

9 The European Molecular Biology Laboratory (EMBL) was officially established as a legal entity on July 4, 1974, when the 1973 intergovernmental Agreement was ratified by the minimum number of signatory countries.

10 The acronym EMBL was officially adopted in 1972. Prior to this, the laboratory was referred to as “EMBO (or EMB) Laboratory.” In this article, I use “EMBO Laboratory.”

11 The European Communities (EC) refers to the three European organizations—the European Coal and Steel Community (ECSC), the European Atomic Energy Community (EURATOM), and the European Economic Community (EEC)—which preceded the foundation of the European Union (EU) in 1992. The ECSC was founded in 1951; the EURATOM and the ECC were established in 1957 by the so-called Treaties of Rome. The Merger Treaty of 1965, ratified in 1967, merged the executive bodies of the ECSC, EURATOM, and EEC into a single Commission and established a single Council of the “European Communities,” which were increasingly frequently referred to in the singular, even though each of the organizations remained legally separate.

12 They included: structure of biological macromolecules; physico-chemical studies of biological macromolecules; protein and nucleic acid chemistry; viruses; immunology; molecular genetics; protein and nucleic acid synthesis; control systems; enzyme function and co-ordination; differentiation and embryology; membranes; the nervous system; energy transfer systems; contractile tissues. See EMBO (1966, p. 19).

13 In 1960 De Gaulle created a new State agency—the *French Délégation Générale à la Recherche Scientifique et Technique* (DGRST)—to promote research in France on an unprecedented scale. Molecular biology was chosen as one of the areas of special intervention (*action concertée*). See Gaudillière (1990).

14 For a detailed analysis of the 1967 session of the EMBC, see Cassata (2026, pp. 205–208).

15 Appleyard was formally appointed part-time Executive Secretary of EMBO, while still retaining his EURATOM post.

16 Initially established in 1963 at the Ravello meeting, the EMBO Laboratory Committee included Cavalli-Sforza, Chantrenne, Engström, Friedrich-Freksa, Jacob, Kendrew (chairman), and Tissières. Adriano Buzzati-Traverso, head of the International Laboratory of Genetics and Biophysics (LIGB) in Naples since 1962, joined the Laboratory Committee in 1966. See *EMBO Activities 1966–1968: A Report to the Volkswagen Stiftung on the Utilisation During 1966, 1967, and 1968 of the Grant of December 1965*, Appendix 3, p. 31, in EMBO Archive, EMBO SEC-C-2-12, https://archive.embo.org/index.php/embo-activities-1966-1968-a-report-to-the-volkswagen-stiftung-on-the-utilisation-during-1966-1967-and-1968-of-the-grant-of-december-1965.

17 Wyman was “greatly impressed” by the neurobiological research that Werner Reichardt was conducting at the MPI for Biology in Tübingen; see Wyman to Kendrew, April 12, 1967, in KP, MSS.Eng.c.2434.

18 Kendrew to Wyman, April 19, 1967 (also to Appleyard, Eigen, Jacob, Porter, Tissières), in KP, MSS.Eng.c.2434.

19 Kendrew to Wyman, April 19, 1967 (also to Appleyard, Eigen, Jacob, Porter, Tissières), in KP, MSS.Eng.c.2434.

20 Jacob to Kendrew, April 17, 1967, in KP, MSS.Eng.c.2434.

21 Tissières to Kendrew, May 3, 1967, in KP, MSS.Eng.c.2434.

22 Tissières to Kendrew, May 23, 1967, in KP, MSS.Eng.c.2434.

23 Eigen to Kendrew, August 7, 1967, in KP, MSS.Eng.c.2434. Eigen’s interviews included a wide range of approaches: G. Braunitzer, Munich (sequence of proteins); F. Cramer, Göttingen (chemical synthesis of nucleotides); H. Friedrich-Freksa, Tübingen (molecular biology of cancer); A. Gierer, Tübingen (molecular genetics, cell differentiation); N. Hilschmann, Göttingen (sequence analysis of Bence-Jones proteins); P. H. Hofschneider, Munich (bacteriophages); W. Hoppe, Munich (X-ray diffraction, phase contrast in electron microscopy); N. Jerne, Frankfurt (immunology); F. Lynen, Munich (biochemistry); E. Ruska, Berlin (electron microscopy); W. Reichardt, Tübingen (mechanisms of sensory perception); R. Schlögl, Frankfurt (membrane transport); G. Schramm, Tübingen (nucleic acids, viruses); H. Schuster, Berlin (molecular genetics); O. Westphal, Freiburg (immunology); H. T. Witt, Berlin (mechanisms of photosynthesis); H.-G. Wittmann, Berlin (molecular genetics); H. G. Zachau, Munich (sequence of transfer RNA); W. Zillig, Munich (polymerases).

24 Eigen to Kendrew, August 7, 1967, in KP, MSS.Eng.c.2434.

25 Porter to Kendrew, May 15, 1967, in KP, MSS.Eng.c.2434.

26 Appleyard to Kendrew, June 20, 1967, in KP, MSS.Eng.c.2434.

27 Appleyard to Kendrew, June 20, 1967, in KP, MSS.Eng.c.2434.

28 Appleyard to Kendrew, April 20, 1967, in KP, MSS.Eng.c.2434.

29 Kendrew to Appleyard, July 24, 1967, in KP, MSS.Eng.c.2434.

30 Delauche to Kendrew, June 30, 1967, in KP, MSS.Eng.c.2434.

31 Kendrew to Wyman, July 12, 1967, in KP, MSS.Eng.c.2434. The meeting would also include “others especially interested in the laboratory plan.”

32 The draft was written in July 1967 and slightly revised in August. Appleyard sent to Kendrew a copy of the first draft on July 13, 1967. See Appleyard, “Proposal for a European Laboratory of Molecular Biology, July 12, 1967,” first draft, in KP, MSS.Eng.c.2434; Appleyard to Kendrew, July 13, 1967, in KP, MSS.Eng.c.2434.

33 “He [Jacob] suggested (as had Tissières) that being neither a ‘moleculist’ nor a ‘cellulist’ I should try and make a first draft programme paper. I don’t know. First papers often determine the general result: so that even the first should really (to my mind) be a Kendrew or a Kendrew–Jacob paper. But if it helps, I’ll try;” Appleyard to Kendrew, July 6, 1967, in KP, MSS.Eng.c.2434.

34 “Private comments of Dr. R. K. Appleyard (Head of the Biology Division of the Euratom, Bruxelles), on the documents prepared by Dr. J. C. Kendrew and Prof. C. H. Waddington for a European Organization of Fundamental Biology,” without date but sent in October 1963, in KP, MSS.Eng.c.2418.

35 Appleyard, “Proposal for a European Laboratory of Molecular Biology,” August 1967, second draft, p. 3, in Pasteur Institute, Monod Papers, MON.Ins.02.

36 Appleyard, “Proposal for a European Laboratory of Molecular Biology,” August 1967, second draft, p. 3.

37 Appleyard, “Proposal for a European Laboratory of Molecular Biology,” August 1967, second draft, p. 4.

38 Appleyard, “Proposal for a European Laboratory of Molecular Biology,” August 1967, second draft, p. 3.

39 Appleyard, “Proposal for a European Laboratory of Molecular Biology,” August 1967, second draft, p. 6.

40 Appleyard, “Proposal for a European Laboratory of Molecular Biology,” August 1967, second draft, p. 7. The research groups included: immunology, morphogenesis, hormones, nervous system, cell genetics and transformation, viruses, enzymes, organelles, bacterial viruses, bacterial genetics, cell design, protein structure, nucleic acid structure.

41 Appleyard, “Proposal for a European Laboratory of Molecular Biology,” August 1967, second draft, p. 8. The technological services included: computers, instrument development, animals, cell stocks and cultures, chemical isolation/purification/production, analysis, electron microscopy, synthesis, immunology, reaction chemistry, histology/cytology.

42 The first draft was dated September 7, 1967; the last version was dated November 1967. See KP, MSS.Eng.c.2434.

43 Kendrew to Chantrenne, September 7, 1967, in EMBO Archive, Correspondence, EMBO EMBOLAB-A, https://archive.embo.org/index.php/correspondence-2026. According to Kendrew, Chantrenne’s criticism towards Appleyard’s draft was “one of main stimuli” that led to the second version of the laboratory project. According to Chantrenne, Appleyard’s draft was “not strong enough to convince government officials;” see Chantrenne to Kendrew, August 15, 1967 (“copied for Lab Committee, 18 August 1967”) in KP, MSS.Eng.c.2434.

44 Kendrew to the members of the EMBO Laboratory Committee, September 7, 1967, in KP, MSS.Eng.c.2434.

45 Kendrew and Wyman, “Proposal for a European Laboratory of Molecular Biology,” Draft, September 7, 1967, p. 1, in KP, MSS.Eng.c.2434.

46 Kendrew and Wyman, “Proposal for a European Laboratory of Molecular Biology,” Draft, September 7, 1967, p. 3, in KP, MSS.Eng.c.2434.

47 Kendrew and Wyman, “Proposal for a European Laboratory of Molecular Biology,” Draft, September 7, 1967, p. 5, in KP, MSS.Eng.c.2434.

48 Kendrew and Wyman, “Proposal for a European Laboratory of Molecular Biology,” Draft, September 7, 1967, p. 5.

49 Kendrew and Wyman, “Proposal for a European Laboratory of Molecular Biology,” Draft, September 7, 1967, p. 6.

50 Kendrew and Wyman, “Proposal for a European Laboratory of Molecular Biology,” Draft, September 7, 1967, p. 8.

51 Kendrew and Wyman, “Proposal for a European Laboratory of Molecular Biology,” Draft, September 7, 1967, pp. 6–8.

52 Kendrew and Wyman, “Proposal for a European Laboratory of Molecular Biology,” Draft, September 7, 1967, p. 11. In November 1967, the Kendrew–Wyman document identified five research themes: structure of proteins in relation to function; the chemical basis of the immune response; nature and structure of the viruses; some aspects of the functioning of the nervous system; “Project K.”

53 Kendrew and Wyman, “Proposal for a European Laboratory of Molecular Biology,” Draft, September 7, 1967, pp. 13–14.

54 Kendrew to the members of the EMBO Laboratory Committee, September 7, 1967, in KP, MSS.Eng.c.2434.

55 Dieudonné to Monod, September 9, 1967; Monod to Dieudonné, September 12, 1967, in Monod Papers, Pasteur Institute. MON. Ins.02, *Réunion EMBO de Nice, 23 Septembre 1967.*

56 Delauche to Kendrew, June 30, 1967, in KP, MSS.Eng.c.2434.

57 “CSP Standing Committee on International Scientific Relations, minutes of the 4^th^ Meeting, May 19, 1967,” p. 4, in KP, MSS.Eng.c.2540. See also “CSP Standing Committee on International Scientific Relations, minutes of the 6^th^ Meeting, December 11, 1967,” p. 2, in KP, MSS.Eng.c.2540.

58 “CSP Standing Committee on International Scientific Relations, minutes of the 4^th^ Meeting, May 19, 1967,” p. 4, in KP, MSS.Eng.c.2540.

59 “CSP Standing Committee on International Scientific Relations, minutes of the 5^th^ Meeting, November 3,, 1967,” p. 2, in KP, MSS.Eng.c.2540.

60 “CSP Standing Committee on International Scientific Relations, minutes of the 6^th^ Meeting, December 11,, 1967,” p. 4, in KP, MSS.Eng.c.2540.

61 Wyman*,* “Notes on meeting of Laboratory Committee, Nice, September 23, 1967,” p. 1, in KP, MSS.Eng.c.2434.

62 Groups and services were assigned to one or more members of the extended EMBO Laboratory Committee, tasked with providing detailed information (manpower, instruments, space, costs): Delauche, “Meeting of the Laboratory Committee, held at Nice, 23^rd^ and 24^th^ September 1967,” Annex 1, in KP, MSS.Eng.c.2434.

63 The outcome of this initiative is not documented in the Kendrew Papers or in other archival collections consulted for this article.

64 Technical information on equipment, space, costs, and manpower was mainly gathered by Appleyard, who also shared statistical figures from EURATOM biological programs. See Appleyard to Kendrew, November 30, 1967, in KP, MSS.Eng.c.2434.

65 Eigen to Kendrew, October 19, 1967, with the appendix “Proposal for a Physical Chemistry Laboratory,” in KP, MSS.Eng.c.2434.

66 De Maeyer, “EMBO Project, Service-Group for Instrumentation,” in KP, MSS.Eng.c.2434.

67 Porter to Kendrew, October 24, 1967, in KP, MSS.Eng.c.2434.

68 Appleyard, “Notes of conversations at Cambridge, November 1–2, 1967,” pp. 5–6, in KP, MSS.Eng.c.2434.

69 Kendrew to Hartley, October 14, 1967, in KP, MSS.Eng.c.2434. See also Appleyard, “Notes of conversations at Cambridge, November 1–2, 1967,” pp. 8–9.

70 Appleyard, “Notes of conversations at Cambridge, November 1–2, 1967,” p. 7.

71 Appleyard, “Notes of conversations at Cambridge, November 1–2, 1967,” pp. 2–3.

72 Appleyard, “Notes of conversations at Cambridge, November 1–2, 1967,” p. 1.

73 Monod, “Outline of Justifications for the Creation of a Preparative Group in the European Molecular Biology Laboratory,” annex 2 of “EMBC. Proposal for a European Laboratory of Molecular Biology prepared by the Council of the European Molecular Biology Organisation, CEBM 68/31, November 1967,” in EMBO Archive, EMBO Laboratory Committee Materials, EMBO EMBOLAB-B-3-19, https://archive.embo.org/index.php/outline-of-justifications-and-specifications-for-the-creation-of-a-preparative-department-in-the-european-molecular-biology-laboratory.

74 Huxley, “Electron Microscopy in the EMBO Laboratory,” annex 3 of “EMBC. Proposal for a European Laboratory of Molecular Biology (CEBM 68/31), November 1967.”

75 Tissières to Kendrew, October 31, 1967, in KP, MSS.Eng.c.2434.

76 Buzzati-Traverso to Kendrew, March 5, 1969, in KP, MSS.Eng.c.2435. In his appendix, Buzzati-Traverso emphasized the potential of the laboratory as European Cold Spring Harbor, a “focal point for advanced experiments in the training of new generations of biologists.” See Buzzati-Traverso, “Teaching at the EMBO Laboratory,” annex 6 of “EMBC. Proposal for a European Laboratory of Molecular Biology (CEBM 68/31 E), November 1967.”

77 Brenner, “Differentiation, Morphogenesis and the Nervous System,” annex 5 of “EMBC. Proposal for a European Laboratory of Molecular Biology (CEBM 68/31 E), November 1967.”

78 Katchalsky, “Project for the Study of ‘Two Dimensional Patterns in Living Cells’”, annex 11 of “EMBC. Proposal for a European Laboratory of Molecular Biology (CEBM 68/31 E), November 1967.”

79 Longuet-Higgins, “Organisms as self-organizing systems,” annex 7 of “EMBC. Proposal for a European Laboratory of Molecular Biology (CEBM 68/31 E), November 1967.”

80 Jacob, “Control Systems,” annex 9 of “EMBC. Proposal for a European Laboratory of Molecular Biology (CEBM 68/31 E), November 1967.”

81 Jerne, “The ‘Complete’ Solution of the Antibody Problem,” annex 8 of “EMBC. Proposal for a European Laboratory of Molecular Biology (CEBM 68/31 E), November 1967.”

82 Delbrück, “An Ex-European looks at a proposal for a European Laboratory,” annex 4 of “EMBC. Proposal for a European Laboratory of Molecular Biology (CEBM 68/31 E), November 1967.”

83 Weisskopf, “The Experience of CERN and the Proposal for a European Biology Laboratory,” annex 10 of “EMBC. Proposal for a European Laboratory of Molecular Biology (CEBM 68/31 E), November 1967.” In September 1968, in his address to the 17^th^ Pugwash Conference on Science and World Affairs in Nice, Weisskopf launched the project for a European Institute of Science and Technology, located in Geneva and articulated into five departments (cosmic sciences, nuclear sciences, electronic sciences, life sciences, and cybernetics). The EMBO Laboratory was explicitly identified as an “excellent nucleus” for the department of life sciences. See Weisskopf, “The European Institute of Science and Technology. Submitted to the Pugwash Meeting. Nice, France, September 1968,” in KP, MSS.Eng.c.2436.

84 Kendrew to all members of EMBO Council and the Laboratory Committee, November 7, 1967.

85 Chantrenne to Kendrew, November 17, 1967, in KP, MSS.Eng.c.2434; Jacob to Kendrew, November 17, 1967, in KP, MSS.Eng.c.2434.

86 Butenandt to Kendrew, November 28, 1967, in KP, MSS.Eng.c.2434 (my translation). See also Kendrew to Butenandt, December 5, 1967, in KP, MSS.Eng.c.2434.

87 Appleyard, “Note of conversation with M. Haunschild,” December 14, 1967, in KP, MSS.Eng.c.2441. Appleyard reacted to Haunschild’s objections by supporting the EMBO Laboratory proposal and playing the different national positions one against the other: “Concerning the laboratory, pointed out to Haunschild that the argument about brain-draining national personnel was amply refuted in the proposal to be submitted, that 2 million $ a year was not a big programme for Germany, that the people around Eigen and Butenandt were the people who had actually done something big, that the project was an imaginative one which would put Europe in the lead even over the US, that it was much better value for money than the big CERN and that if it was a question of not letting the French have all the winnings, then there should be ample matter for discussion with M. Gordon Walker in January.”

88 Appleyard, “Note of conversation with M. Haunschild, December 14, 1967,” in KP, MSS.Eng.c.2441.

89 Wyman, “Notes on talks with François Jacob, December 22, 1967,” in KP, MSS.Eng.c.2441.

90 “CSP Standing Committee on International Scientific Relations, minutes of the 6th Meeting, 11 December 1967),” p. 2, in KP, MSS.Eng.c.2540,

91 Kendrew to Weisskopf, November 28, 1967, in KP, MSS.Eng.c.2434.

92 “EMBC, Second session, Definitive Summary Records of the Meetings held at CERN, Geneva, on 22, 23, 24 and 25 January 1968, CEBM 68/45, II session/PV 1,” pp. 13–15, in EMBO Archive: Second Session of the European conference on Molecular Biology, EMBO EMBC-A-3, https://archive.embo.org/index.php/second-session-of-the-european-conference-on-molecular-biology.

93 “EMBC, Draft Agreement establishing a European Conference on Molecular Biology. Note from the British Government, CEBM 68/38, December 21, 1967,” in EMBO Archive: Second Session of the European conference on Molecular Biology, EMBO EMBC-A-3, https://archive.embo.org/index.php/second-session-of-the-european-conference-on-molecular-biology.

94 The UK proposal was radically rejected by EMBO and by the delegations of France, Italy, Austria, Greece, and Spain. Switzerland declared to be “somewhat embarrassed” by the UK’s alternative project. The Netherlands, West Germany, and the Scandinavian delegations were prepared to take note of certain critical points raised by the UK but supported the EMBC Draft Agreement. The British opposition was not only overruled in the definition of the Draft Agreement but also during the discussion of the single articles of the Treaty. The requests made by the UK delegation, aimed at weakening the EMBC as much as possible (such as reducing the duration of the Agreement to three years instead of five, and requiring unanimous decisions rather than a two-thirds majority for the adhesion to Special Projects) were all rejected. For more details on this see Cassata (2026, pp. 213–214).

95 Article II.3 allowed for the possibility of Special Projects—that is, projects supported by only some members states—to be approved by a two-thirds majority. The execution of a Special Project had to be the subject of a special agreement between the States participating in it, and any Member was entitled to become associated at any later time with a Special Project which had already been approved.

96 J. W. [Wyman], “Comments on EMBO Conference, Geneva, January 1968,” January 26, 1968, in KP, MSS.Eng.c.2441.

97 Since 1965 the Austrian Academy of Science had been planning the establishment of an Institute of Molecular Biology in Vienna. Perutz and Butenandt were external advisors of the preparatory commission. See Feichtinger et al. (2022, pp. 338–341).

98 “EMBC, Second session. Definitive Summary Records of the Meetings held at CERN, Geneva, on 22, 23, 24 and 25 January 1968 (CEBM 68/45, II session/PV 2),” pp. 79–80, in EMBO Archive, EMBC Materials, Second Session of the European conference on Molecular Biology, EMBO EMBC-A-3, https://archive.embo.org/index.php/second-session-of-the-european-conference-on-molecular-biology.

99 The expression “American challenge” was popularized by *Le défi américain*, Jean-Jacques Servan-Schreiber’s best-selling book foretelling European subordination to American technological supremacy, which was published in November 1967 and translated into English in 1968 (see Servan-Schreiber 1967).

100 “Memorandum of the meeting with Volkswagen in Hanover,” December 3, 1965; “Minutes of EMBO meeting at Tübingen,” March 22, 1964, in KP, MSS.Eng.c.2422.

101 Perutz to Kendrew, Wyman, Jacob, and J. A. Cohen, “Note on a telephone conversation with Butenandt on 8th April 1968 concerning the German refusal to sign the draft Agreement (confidential),” in KP, MSS.Eng.c.2441.

102 “Extract from the Recommendations made by the ad-hoc Committee on European Collaboration in the Field of Molecular Biology to the Federal Ministry for Scientific Research, Bonn, on 26^th^ September, 1968, CEBM/Lab/71/9,” pp. 3–5, in EMBO Archive, EMBO Laboratory Committee Materials, Report Rendered to the European Molecular Biology Conference, EMBO EMBOLAB-B-3-20, https://archive.embo.org/index.php/report-rendered-to-the-european-molecular-biology-conference.

103 “CSP Standing Committee on International Scientific Relations, March 21, 1968, Minutes of the meeting,” p. 4, in KP, MSS.Eng.c.2540. In January 1969, the Committee endorsed the establishment of a European Research Council as a “consultative body,” with “absolute control over a comparatively small budget,” acting as a “forum” of consultation about European science policy and preventing the emerging of a “situation in which scientific priorities are determined by response to pressure groups.” The reference to “pressure groups” clearly hinted at EMBO and EMBC. See “CSP Standing Committee on International Scientific Relations. Contribution on International Scientific Relations to the Third Report of the Council for Scientific Policy, January 3, 1969,” p. 8, in KP, MSS.Eng.c.2541.

104 E. Vallotton, “Note de dossier. ‘Regio genevensis’,” December 1, 1967, in Swiss Federal Archives (hereafter BAR), o.144.151, Laboratoire Européen de Biologie Moléculaire, EMBO, CEBM (1967-1969).

105 The 1969 EMBO Laboratory Committee included Maaløe (Chairman), Buzzati-Traverso, Crick, Brenner, Jacob, Jerne, Kendrew, Luzzati, Tissières, Wyman, and Zachau. See “Minutes of the EMBO Council Meeting held at CERN, Geneva, on January 12, 1969,” p. 4, in EMBO Archive, EMBO Council Secretariat Material, 9^th^ EMBO Council Meeting at CERN, EMBO SEC-A-15, https://archive.embo.org/index.php/embo-council-meeting-at-cern-february-1969.

106 “Minutes of the EMBO Laboratory Committee Meeting held at Linton, March 16, 1969,” in KP., MSS.Eng.c.2435.

107 “Minutes of the EMBO Laboratory Committee Meeting held at Linton, March 16, 1969.”

108 “Minutes of the EMBO Laboratory Committee Meeting held at Linton, March 16, 1969.”

109 “Minutes of the EMBO Laboratory Committee Meeting held at Linton, March 16, 1969.”

110 “Minutes of the EMBO Laboratory Committee Meeting held at Linton, March 16, 1969.”

111 “Minutes of the EMBO Laboratory Committee Meeting held at Linton, March 16, 1969.”

112 After completing his doctorate under Butenandt in Tübingen, Zachau had spent the years 1955–58 in postdoctoral training at MIT with J. C. Sheehan and at Rockefeller University, where he worked with Fritz Lipmann on protein biosynthesis and tRNA. See Wenkel and Deichmann (2007, pp. 94–96).

113 Zachau to Maaløe, May 14, 1969, in KP, MSS.Eng.c.2435. With regard to Munich, Zachau also mentioned the Society for Radiation Research (*Gesellschaft für Strahlenforschung*, GSF), which focused on biophysics and radiation biology. In addition, the report referred to the *Sonderforschungsbereiche*, Collaborative Research Centers, established in 1968 by the *Deutsche Forschungsgemeinschaft* (DFG)—the German Research Foundation—on the recommendation of the *Wissenschaftsrat* (German Science Council), to strengthen university research.

114 On Sweden: Bertani to Maaløe, May 28, 1969, in KP, MSS.Eng.c.2435; on the Netherlands: Rörsch to Kendrew, May 13, 1969; on Switzerland: “Information received on April 10, 1969, from Dr. A. Tissières about laboratory developments in Switzerland,” May 1969; on Belgium: Chantrenne to Kendrew, May 23, 1969; on Great Britain: Kendrew and Brenner, “Molecular Biology in the UK;” on France: Luzzati to Maaløe, April 22, 1969, in KP, MSS.Eng.c.2435.

115 On Austria: H. Tuppy, “Laboratory developments in Austria,” June 1, 1969; on Spain: Subirana to Kendrew, May 10, 1969; on Greece: Evangelopoulos to Kendrew, April 30, 1969, in KP, MSS.Eng.c.2435. For the role of EMBO in the emergence of Spanish molecular biology research groups, see Santesmases (2002).

116 “Minutes of the EMBO Council meeting held at CERN, Geneva, June 1, 1969,” p. 2, in EMBO Archive, EMBO Council Secretariat Material, 13^th^ EMBO Council Meeting at CERN, EMBO SEC-A-19, https://archive.embo.org/index.php/embo-council-meeting-at-cern-june-1969.

117 “Notes on the meeting of the Laboratory Committee, June 1, 1969,” p. 1, in EMBO Archive, EMBO Laboratory Committee Materials, EMBO EMBOLAB-B-1-4, https://archive.embo.org/index.php/notes-on-the-meeting-of-the-laboratory-committee.

118 “Minutes of the EMBO Council meeting held at CERN, Geneva, June 1, 1969,” p. 2. See also: Weisskopf to Vallotton, March 3, 1969; Vallotton to Weisskopf, April 18, 1969; Jacob to Weisskopf, March 31, 1969; in KP, MSS.Eng.c.2436.

119 Kendrew to Gregory, June 20, 1969, in KP, MSS.Eng.c.2436.

120 “Notes on the meeting of the Laboratory Committee, June 1, 1969,” p. 1. On reactions in Sweden, see also Zetterberg (1976, pp. 47–55).

121 “Notes on the meeting of the Laboratory Committee, June 1, 1969,” p. 2.

122 The CSP Working Party on the Proposed European Laboratory for Molecular Biology included H. Massey (chair), J. B. Adams, J. Baddiley, J. H. Humphrey, M. G. P. Stoker, W. L. Francis (Science Research Council), J. A. B. Gray (Medical Research Council), J. C. Kendrew (chairman of the CSP Working Group on Molecular Biology). J. F. Embling (DES).

123 “Council for Scientific Policy, Working Party on the Proposed Laboratory for Molecular Biology, 2^nd^ Meeting Minutes, March 20, 1969,” p. 3, in KP, MSS.Eng.c.2542.

124 “Council for Scientific Policy, Discussions between CSP and Representatives of the Wissenschaftsrat,” Annex 2, p. 1, in KP, MSS.Eng.c.2537.

125 “Council for Scientific Policy, Working Party on the Proposed Laboratory for Molecular Biology, Draft Report, March 14, 1969, Appendix (Comparison with CERN),” p. 32, in KP, MSS.Eng.c.2542.

126 “Council for Scientific Policy, Discussions between CSP and Representatives of the Wissenschaftsrat,” Annex 2, p. 2.

127 Brenner had launched this suggestion just a few days earlier, in a letter to the members of the EMBO Laboratory Committee. See Brenner, “Note to members of EMBO Laboratory Committee, May 27, 1969,” in KP, MSS.Eng.c.2435.

128 “Notes on the meeting of the Laboratory Committee, June 1, 1969,” p. 3. Wyman was entrusted to make a first try with Villa Falconieri in Rome as a possible venue. Other options, suggested by Appleyard, were CERN in Geneva or CIBA in Basel.

129 “Interim European Molecular Biology Conference. Provisional Summary Records of the Meetings, CERN, 2–3 June 1969 (CEBM/int/11),” p. 48, in EMBO Archive, EMBC Material, EMBO EMBC-A-4, https://archive.embo.org/index.php/meeting-of-the-interim-european-conference-on-molecular-biology-at-cern. The European Unit of Account was introduced as the internal unit of account for the European Payments Union when that organization was formed in 1950. The EUA was defined as 0.888671 grams of gold, or one US dollar. The cost was covered by the Volkswagen Foundation’s grant.

130 “Interim European Molecular Biology Conference, Proposed European Laboratory for Molecular Biology, The issues involved: Note by the United Kingdom Delegation (CEBM/int/10),” in EMBO Archive, EMBC Material, EMBO EMBC-A-4, https://archive.embo.org/index.php/meeting-of-the-interim-european-conference-on-molecular-biology-at-cern.

131 Embling to P. N. Rasmussen (Danish Science Advisory Council), Pierre Aigrain (DGRST Director), F. Scheidemann (Federal Ministry for Scientific Research), Friedrich Schneider (MPG Secretary General), Vincenzo Caglioti (President of the Italian National Council for Research, CNR), Carl Johan Friedrich Bottcher (Gaubius Institute, Leiden), Odd Austveg (Deputy Director of the Norwegian Ministry of Industry), Arne Engström (Secretary General of the Swedish Science Advisory Council), Urs Hochswasser (Swiss Department of Interior, Division of Science and Research), May 20, 1969, Annex to “Council for Scientific Policy, Working Party on the Proposed European Laboratory for Molecular Biology. The Issues, CSP (EB)(69)3,” in KP, MSS.Eng.c.2542.

132 Embling to Rasmussen, Aigrain, Scheidemann, Schneider, Caglioti Bottcher, Austveg, Engström, Hochswasser, May 20, 1969, in KP, MSS.Eng.c.2542.

133 David Christie Martin (Executive Secretary of the Royal Society) to the Fellows of the Royal Society, September 24, 1969, in KP, MSS.Eng.c.2435.

134 “The Royal Society. Discussion on UK participation in the European Molecular Biology Organization. Paper by M. G. P. Stoker, The United Kingdom and the Proposed EMBO Laboratory, 21 October 1969,” p. 4, in KP, MSS.Eng.c.2435.

135 “Paper by M. G. P. Stoker, The United Kingdom and the Proposed EMBO Laboratory, 21 October 1969,” pp. 1–2.

136 “Paper by M. G. P. Stoker, The United Kingdom and the Proposed EMBO Laboratory, 21 October 1969,” p. 4.

137 “The Royal Society. J. C. Kendrew, The European Molecular Biology Organization and its proposal for an International Laboratory, 6 October 1969,” p. 2, in KP, MSS.Eng.c.2435. In his speech, Kendrew also justified the higher budget of the EMBO Laboratory in comparison with UK laboratories.

138 “Speech by Sydney Brenner. Draft,” p. 1, in KP, MSS.Eng.c.2435.

139 “Speech by Sydney Brenner. Draft,” p. 5.

140 Anon., “EMBO runs into British opposition,” *Scientific Research*, November 24, 1969, p. 17; Anon., “The EMBO Question Debated,” *Nature*, 224, November 1, 1969, pp. 406–407. See on this de Chadarevian (2002, p. 330), Krige (2013).

141 They were: R. K. Appleyard, S. Brenner, H. Chantrenne, A. Buzzati-Traverso, A. Engström, M. Eigen, F. Jacob, N. K. Jerne, A. Katchalski, J. C. Kendrew, V. Luzzati, O. Maaløe, R. Monier, M. F. Perutz, M. G. P. Stoker, A. Tissières, H. Tuppy, H. G. Zachau, H.-G. Wittmann, J. Wyman. See “List of participants, EMBO-Meeting, November 27–30,1969, ‘final version’,” hand–written by Kendrew, in KP, MSS.Eng.c.2435.

142 From UK: Arnold S.V. Burgen (Molecular Pharmacology Research Unit, Cambridge); Francis Crick (Cambridge); Aaron Klug (Cambridge); Peter Lawrence (LMB, Cambridge); David Marr (Trinity College, Cambridge); Ricardo Miledi (UCL), John Paul (director of the Beatson Institute for Cancer Research, Glasgow); David Chilton Phillips (Oxford), Martin R. Pollock (Edinburgh); from France: Jean Pierre Changeux (Pasteur); Boris Ephrussi (Gif-sur-Yvette); Michel Jouvet (Lyon); from West Germany: Thomas Jovin (MPI physical chemistry, Göttingen), Günter Maass (MPI physical chemistry, Göttingen), Werner E. Reichardt (MPI for biological cybernetics, Tubingen); from Italy: Paolo Fasella; from Sweden: George Klein (Department of tumor biology, Karolinska); from the US: David Hunter Hubel (Harvard Medical School); Cyrus Levinthal (Columbia, USA); Sir John Eccles (Biochemistry Department, NYU at Buffalo); James Watson (Harvard University); Victor Weisskopf (MIT). See “List of participants, EMBO-Meeting, November 27–30,1969, ‘final version’ (hand–written by Kendrew),” in KP, MSS.Eng.c.2435. Bernard Gregory (CERN), Renato Dulbecco, Eugene Kennedy (Harvard Medical School), Salvador Luria (MIT), Sir Peter Medawar, and Jacques Monod were also invited but did not attend the Konstanz meeting. Additional names “suggested for Konstanz” but not invited were: Hugh E. Huxley (LMB, Cambridge), Avrion Mitchison (Mill Hill), Horace B. Barlow (Berkeley), John B. Gurdon (Oxford), Lewis Wolpert (London), Robert Hinde, Max Delbruck, Peter Geiduschek, Walter Gilbert, Stephen W. Kuffler, Joshua Lederberg, Lederley [sic], Richard Darwin Keynes (ARC Institute of Animal Physiology, Babraham), Lubert Stryer (Yale), Henri Buc (Pasteur) and Harvey Eisen (Pasteur). On this see Kendrew’s notes in KP, MSS.Eng.c.2435.

143 Brenner, “Note to members of EMBO Laboratory Committee, May 27, 1969,” in KP, MSS.Eng.c.2435.

144 See, for instance, Kendrew to all members of EMBO Council, Laboratory Committee and Provisional Scientific Advisory Committee, March 9, 1972, in KP, MSS.Eng.c.2436. For this reason, the article retains the German toponym “Konstanz” rather than the English “Constance.”

145 I draw on the category of “epistemic mainstreaming” from Schwerin and Kraft (2024, p. 351).

146 “Notes on EMBO meeting to discuss future developments in molecular biology, and in particular the EMBO Laboratory project, Constance, November 27–30, 1969. Thursday, November 27, morning session,” p. 1, in KP, MSS.Eng.c.2435. For the agenda of the meeting, see: “EMBO Meeting from November 27^th^ through 30^th^, 1969 – Agenda,” in EMBO Archive, EMBO Laboratory Committee Meeting Documents, EMBO EMBOLAB-B-1, https://archive.embo.org/index.php/embo-laboratory-committee-meeting-documents.

147 “Notes on EMBO meeting to discuss future developments in molecular biology, and in particular the EMBO Laboratory project, Constance, November 27–30, 1969. Thursday, November 27, morning session,” pp. 2–3.

148 “Notes on EMBO meeting to discuss future developments in molecular biology, and in particular the EMBO Laboratory project, Constance, November 27–30, 1969. Thursday, November 27, morning session,” p. 5 (Perutz’s expression).

149 “Notes on EMBO meeting to discuss future developments in molecular biology, and in particular the EMBO Laboratory project, Constance, November 27–30, 1969. Thursday, November 27, morning session,” p. 4.

150 “Notes on EMBO meeting to discuss future developments in molecular biology, and in particular the EMBO Laboratory project, Constance, November 27–30, 1969. Thursday, November 27, morning session,” p. 6.

151 “Notes on EMBO meeting to discuss future developments in molecular biology, and in particular the EMBO Laboratory project, Constance, November 27–30, 1969. Thursday, November 27, morning session,” p. 6 (Interventions by Watson, Maaløe, Weisskopf, Jovin).

152 “Notes on EMBO meeting to discuss future developments in molecular biology, and in particular the EMBO Laboratory project, Constance, November 27–30, 1969. Friday, November 28, morning session” and “Saturday. November 29, morning session.” p. 1.

153 “Notes on EMBO meeting to discuss future developments in molecular biology, and in particular the EMBO Laboratory project, Constance, November 27–30, 1969. Friday, November 28, morning sessions,” p. 2. In 1968, Jacob had proposed the establishment of a “Mouse Institute” to the DGRST. See Morange (2000) and Méthot (2020).

154 “Notes on meeting of Laboratory Committee, November 30, 1969,” p. 1, in KP, MSS.Eng.c.2435.

155 “Notes on meeting of Laboratory Committee, November 30,1969,” p. 1.

156 “Notes on meeting of Laboratory Committee, November 30, 1969,” p. 1.

157 *Subcellular structures*: Klug, Holmes, Burgen, Luzzati (convener); *Cell genetics*: Klein, Jacob, Stoker, Brenner (convener); *Instrumentation and computation*: Hoppe, Hartley, Huxley (convener), De Maeyer, Marshall, Zachau, “X from CERN” (then M. Hine), “Y from Stoker’s Laboratory.” “Notes on meeting of Laboratory Committee, November 30, 1969,” p. 4.

158 “Notes on meeting of Laboratory Committee, November 30, 1969,” p. 1.

159 *Macromolecular assemblies*: Klug, Holmes, Phillips, Huxley, Hoppe, Rusker, Schoenborn, Caspar, Cohen, Langridge, Moody, Gibbons, Davies, Edmond, Poljak, Luzzati, Liquori, Perutz, Drenth, Brändén, Huber, Cosletti. *Membranes*: Wilkins, Slater, Racker, Delbrück, Leugen [sic], Hodgkin, Keynes, Stoeckenius, van Deynen [sic, probably van Deenen], Davis, Müller, Kepes, Changeux, Luzzati, Shulman, Katchalski, Dejenner [sic, probably Jan de Gier] , Skoe [sic, probably Skou], Weber, Morel. *Cell biology*: Attardi, Palade, Dulbecco, Gurdon, Paul, Stoker, Klein, Cairns, Jerne, Ephrussi, Beermann, von Wettstein, Girard, Lwoff, Mitchison, Gowers, Callan, Baglioni, Brachet, Barnett, Koprowsky, Monroy, Walker, Slonimski, Ingram, Pontecorvo, Luria, Szent-Györgyi, Geiduschek, Boyce, Sambrook, Jacob, Monod. *Neurobiology*: Longuet-Higgins, Hubel, Wiesel, Marr, Reichardt, Taugh, Köffler, Brenner, Benzer, Barlow, Whittaker, Nicholls, Engström, Miledi, Eccles, Hodgkin, Crick, Glowinski. *Proteins and Nucleic acids*: Edmund, Baglioni, Guidotti, Scoffone, Merrifield, Hartley, Biemann, Smithies, Staehelin, Edelman, Haber, Sanger, Wittmann, Tiselius, Porath, Gray, Weber, Khorana, Kellenberger. “Notes on meeting of Laboratory Committee, November 30, 1969,” pp. 2–3. The list reproduces the original notes.

160 “Notes on meeting of Laboratory Committee, November 30, 1969,” p. 3.

161 *Revised Proposal for a European Laboratory of Molecular Biology* (CEBM/70/12), p. 2, in EMBO Archive, EMBO Laboratory Committee Materials, EMBO EMBOLAB-B-2-6, https://archive.embo.org/index.php/revised-proposal-for-a-european-laboratory-of-molecular-biology.

162 Kendrew, “Summary of EMBO’s new proposals for an international laboratory, January 1, 1970,” in KP, MSS.Eng.c.2435.

163 “Revised Proposal for a European Laboratory of Molecular Biology (CEBM/70/12),” p. 3.

164 “Revised Proposal for a European Laboratory of Molecular Biology (CEBM/70/12),” p. 5.

165 “Revised Proposal for a European Laboratory of Molecular Biology (CEBM/70/12),” p. 1.

166 On DESY and the development of storage rings in the late 1960s and 1970s, see Cramer (2020, pp. 62–63).

167 “Revised Proposal for a European Laboratory of Molecular Biology, Annex D–Report of the Working Group on Techniques and Instrumentation in the EMBO Laboratory (CEBM/70/12),” p. 46.

168 “Revised Proposal for a European Laboratory of Molecular Biology, Annex D–Report of the Working Group on Techniques and Instrumentation in the EMBO Laboratory (CEBM/70/12),” p. 49. The development of advanced techniques in nuclear magnetic resonance and in protein chemistry (automation of sequence analysis, mass spectrometry, peptide synthesis, etc.), together with the growing use of “methods for recording and processing data” such as “very large computers,” was considered essential for the future EMBO Laboratory.

169 “Revised Proposal for a European Laboratory of Molecular Biology, Annex D–Report of the Working Group on Techniques and Instrumentation in the EMBO Laboratory (CEBM/70/12),” p. 50.

170 “Revised Proposal for a European Laboratory of Molecular Biology (CEBM/70/12),” p. 11.

171 Kendrew to Holmes, May 29,1970; Martin W. Teucher (member of the Executive Board of DESY) to Kendrew, May 6, 1970, in EMBO Archive, Correspondence, EMBO Council Secretariat Material, Correspondence, EMBO SEC-E, https://archive.embo.org/index.php/correspondence-2028.

172 “A Proposal for the EMBO Synchrotron Radiation Bunker at DESY Hamburg, August 1971 (CEBM/Lab/71/31),” in EMBO Archive, EMBO Laboratory Committee Materials, EMBO EMBOLAB-B-2-12, https://archive.embo.org/index.php/proposal-for-the-embl-synchrotron-radiation-bunker-at-desy-hamburg.

173 “Preliminary proposal for an EMBO neutron diffraction facility at Grenoble, October 1971 (CEBM/Lab/71/32),” in EMBO Archive, EMBO Laboratory Committee Materials, EMBO EMBOLAB-B-2-17, https://archive.embo.org/index.php/vorlaufige-vorschlag-fur-eine-embo-einrichtung-zur-neutronen-beugung-in-grenoble.

174 “Revised Proposal for a European Laboratory of Molecular Biology (CEBM/70/12),” p. 3.

175 Wyman to Maaløe, December 11, 1969, in KP, MSS.Eng.c.2435. In March 1970, Kendrew asked Watson to send him a copy of the 1969 Annual Report of the Cold Spring Harbor Laboratory, describing it as “excellent reading” that could influence the views of the members of the CSP Working Party on the Proposed European Laboratory for Molecular Biology: Kendrew to Watson, March 16, 1970; Kendrew to E. C. Appleyard, May 26, 1970; “CSP Working Party on the Proposed European Laboratory for Molecular Biology, Note by Secretaries: ‘Cold Spring Harbor Laboratory of Quantitative Biology – 1969 Annual Report,’ CSP(EB)(70)6,” in KP, MSS.Eng.c.2542.

176 “Revised Proposal for a European Laboratory of Molecular Biology (CEBM/70/12),” p. 13.

177 “Revised Proposal for a European Laboratory of Molecular Biology (CEBM/70/12),” p. 25.

178 “Revised Proposal for a European Laboratory of Molecular Biology (CEBM/70/12),” p. 23.

179 “Revised Proposal for a European Laboratory of Molecular Biology (CEBM/70/12),” pp. 23–25.

180 “Revised Proposal for a European Laboratory of Molecular Biology (CEBM/70/12),” p. 22. Significantly, Hine partially contested this analogy, emphasizing that CERN was a more effective model as an “international centre” dedicated to “pure research,” while other sites—Harwell, Rutherford, Culham—were “more powerful” in terms of know–how but they were “national laboratories” and would “inject a biased, not European know–how.” See Gregory to Kendrew, January 22, 1970, in CERN Archives, CERN Directors–General (First series), External Relations – International Center for Biology (EMBO), 1965–1975, B12, DG-20685.

181 “Revised Proposal for a European Laboratory of Molecular Biology (CEBM/70/12),” p. 23.

182 In January 1970, Kendrew prepared a summary of the revised proposal ahead of the meeting of the CSP Working Party on the Proposed European Laboratory for Molecular Biology: see Kendrew, “Summary of EMBO’s new proposals for an international laboratory, January 1, 1970,” in KP, MSS.Eng.c.2435.

183 “CSP Working Party on the Proposed European Laboratory of Molecular Biology, Minutes of the meeting of February 17, 1970,” pp. 3–4, in KP, MSS.Eng.c.2542. The final report of the Working Group was submitted to the CSP on March 20, 1970: “CSP Working Party on the Proposed European Laboratory of Molecular Biology. The Working Party’s Draft Report, March 26, 1970,” in KP, MSS.Eng.c.2542.

184 “EMBC first ordinary session (1970), CERN, Geneva, April 6–8, 1970 (CEBM/70/19),” p. 89, in EMBO Archive, EMBC Material, EMBO EMBC-A-5, https://archive.embo.org/index.php/meeting-of-the-european-molecular-biology-conference-in-geneva.

185 “EMBC first ordinary session (1970), CERN, Geneva, April 6–8, 1970 (CEBM/70/19),” p. 89.

186 The “spirit of The Hague” label was popularized in late 1969–early 1970 in contemporary press and political contexts. By January 1971, it was established enough for French President Georges Pompidou to publicly define it at a press conference: https://www.ina.fr/ina-eclaire-actu/video/caf94060380/conference-de-presse-de-monsieur-pompidou. Accessed 15 June 2026.

187 G. Hess to Eigen, April 28, 1970, in EMBO Archive, EMBO Laboratory Committee Material, Correspondence, EMBO EMBOLAB-A, https://archive.embo.org/index.php/correspondence-2026.

188 *Establishment of a European Laboratory for Molecular Biology – Meeting at the Federal Ministry of Education and Science on May 25, 1970*, Bonn, June 3, 1970, in EMBO Archive, EMBO Laboratory Committee Materials, EMBO EMBOLAB-B-1, https://archive.embo.org/index.php/embo-laboratory-committee-meeting-documents.

189 Edmund Marsch (MPG General Administration) to Eigen, July 31, 1970, in EMBO Archive, EMBO Laboratory Committee Materials, Correspondence, EMBO EMBOLAB-A, https://archive.embo.org/index.php/correspondence-2026.

190 See on this Renn et al. (2024, pp. 674-676)

191 Wyman to Kendrew, July 1970 (handwritten), in KP, MSS.Eng.c.2436.

192 Wyman to Kendrew, July 1970 (handwritten).

193 Kendrew to Rörsch, August 26, 1970, in KP, MSS.Eng.c.2446.

194 See on this topic Deichmann (2002, pp. 461–464).

195 Sadron to Perutz, March 13, 1964, in KP, MSS.Eng.c.2422. Sadron was referring to nationalism as a general problem, not just on West Germany’s part.

196 Perutz to Wyman, March 17, 1964, in KP, MSS.Eng.c.2422 (emphasis is mine). See also Kellenberger to Perutz, March 10, 1964, in KP, MSS. Eng.c.2422.

197 Rörsch to Eigen, August 11, 1970, in EMBO Archive, EMBO Laboratory Committee Materials, Correspondence, EMBO EMBOLAB-A, https://archive.embo.org/index.php/correspondence-2026.

198 Embling to Dainton, August 25, 1970, in KP, MSS.Eng.c.2436.

199 Embling to Dainton, August 25, 1970.

200 Embling to Dainton, August 25, 1970.

201 Embling to Dainton, August 25, 1970.

202 UK representatives were Embling and Appleyard, both from the DES, and Dainton, CSP Chairman; the German representatives were Zelle (Ministry of Education and Science) and Engelhardt (Ministry of Foreign Affairs). See “Proposal to establish a European Molecular Biology Laboratory. Note of a discussion at DES about possible sites, September 3, 1970,” in KP, MSS.Eng.c.2436.

203 “Proposal to establish a European Molecular Biology Laboratory. Note of a discussion at DES about possible sites, September 3, 1970,” p. 1.

204 “Proposal to establish a European Molecular Biology Laboratory. Note of a discussion at DES about possible sites, September 3, 1970,” p. 1.

205 “Proposal to establish a European Molecular Biology Laboratory. Note of a discussion at DES about possible sites, September 3, 1970,” p. 1.

206 “Proposal to establish a European Molecular Biology Laboratory. Note of a discussion at DES about possible sites, September 3, 1970,” p. 1.

207 R. K. Appleyard to E. C. Appleyard, September 2, 1970, in EMBO Archive, EMBC Material, Correspondence with Member States, EMBO EMBC-B-2, Box “United Kingdom.” see also Appleyard to Kendrew, August 31, 1970, in KP, MSS.Eng.2443.

208 For the list of the participants, see Appleyard’s draft “Monday, 28^th^ September 1970, Meeting of EMBO with the representatives of national scientific institutions,” in EMBO Archive, Box “Laboratory Committee.” Participants included: France: F. Jacob; UK: D. C. Phillips (Laboratory of Molecular Biophysics, University of Oxford); West Germany: H. Brieskorn (BBW, Bonn), H. G. Zachau (Institute of Physiological Chemistry, Munich); Denmark: A. Rörsch (University of Leiden), N. J. Koch-Kijlstra (Ministry of Education and Science, The Hague); The Netherlands: E. C. Slater (Laboratory of Biochemistry, Amsterdam), L.N.M. Duysens (Laboratory of Biophysics, Leiden); Switzerland: H. Voirier (Federal Political Department, International Organizations, Bern), E. Freitag (Federal Department of Home Affairs, Division of Science and Research, Bern), A. Tissières (University of Geneva); Italy: O. Ciferri (Institute of Genetics, Pavia), P. Fasella (Institute of Biochemistry, Parma); Belgium: P. Levaux (FNRS), L. Vandendriessche (FNRS); Greece: G. Akoyunoglou (Greek Atomic Energy Commission), Evangelopoulos (University Clinic Alexandra); Israel: S. Lifson (Weizmann Institute of Science); Norway: A. P. Nygaard (Department of Biochemistry, Bergen), S. G. Laland (Department of Biochemistry, Blindern University, Oslo). Delegates from Austria and Sweden are not specified in the list.

209 Maaløe to all EMBO members, August 17, 1970, in CERN Archives, CERN Directors–General (First series), External Relations–International Center for Biology (EMBO), 1965–1975, B12, DG-20685.

210 “Results of questionnaire to EMBO members,” in EMBO Archive, Folder “Laboratory Committee,” in EMBO Archive, EMBO Laboratory Committee Materials, EMBO EMBOLAB-B-1, https://archive.embo.org/index.php/embo-laboratory-committee-meeting-documents.

211 “EMBC, Summary record of the Meeting of the Study Group for the European Laboratory, The Hague, 29^th^ September 1970 (CEBM/70/28),” p. 3, in EMBO Archive, EMBC Material, 1^st^ Ordinary Meeting of the European Molecular Biology Conference, EMBO EMBC-A-6, https://archive.embo.org/index.php/1st-ordinary-meeting-of-the-european-molecular-biology-conference.

212 Voirier, “Note pour le Chef de la Division des organisation internationales,” October 7, 1970, p. 1, in BAR, E 2003 A 1984/84, BD : 278, o.144.511, Laboratoire européen de biologie moléculaire, CEBM, 1970/72.

213 Members of the EMBC Study Group were national delegates to the EMBC. Most had already participated in the meeting of September 28. See correspondence in EMBO Archive, EMBO Laboratory Committee Materials, Administrative documents, “List of the attendants of the EMBO meeting with representatives of scientific institutions,” EMBO EMBOLAB-B-1-6, https://archive.embo.org/index.php/list-of-attendants-of-the-embo-meeting-with-representatives-of-scientific-institutions.

214 “EMBC, Summary record of the Meeting of the Study Group for the European Laboratory, The Hague, 29^th^ September 1970 (CEBM/70/28),” p. 12. In a long letter sent to Kendrew in December 1970, biochemist Edward Charles (“Bill”) Slater (University of Amsterdam) provided Kendrew with historical background on the position of the Netherlands—and particularly the Commission of Biochemistry and Biophysics of the Royal Academy of Science, of which he was chairman—towards the EMBO Laboratory: “The UK proposal of three EMBO laboratories—he claimed—did not receive any support in our Commission. The passages quoted from the 1969 letter show that we have in mind one strong EMBO Laboratory, with good laboratories in each of the participating countries:” Slater to Kendrew, December 7, 1970, in KP, MSS.Eng.c.2461.

215 “EMBC, Summary record of the Meeting of the Study Group for the European Laboratory, The Hague, 29^th^ September 1970 (CEBM/70/28),” p. 11. See also “Analysis of the aspects of Greece which will be submitted by the Greek representative at The Hague Conference of the 29^th^ September on the European Laboratory of Molecular Biology,” in KP, MSS.Eng.c.2443.

216 “EMBC, Summary record of the Meeting of the Study Group for the European Laboratory, The Hague, 29^th^ September 1970 (CEBM/70/28),” p. 29.

217 Voirier, “Note pour le Chef de la Division des organisations internationales,” October 7, 1970, p. 2*.*

218 Voirier, “Note pour le Chef de la Division des organisations internationales,” October 7, 1970, p. 2.

219 Voirier, “Note pour le Chef de la Division des organisations internationales,” October 7, 1970, p. 3.

220 Friedrich-Freksa to Perutz (and cc Butenandt and Eigen), March 18, 1967 (in German), in KP, MSS.Eng.c.2441.

221 “Appointment by EMBO Council of a Committee to study the siting of the proposed European laboratory, The Hague, September 30, 1970,” in KP, MSS.Eng.c.2436. Delauche served as secretary, while Phillips, Zachau and Tissières acted as consultants

222 Hughes to Holmes, October 4, 1970, in KH, 112 P-HOL-C (1 of 2), EMBC Sites Committee – Correspondents A–M.

223 Holmes to Hughes, October 9, 1970.

224 Holmes to Hughes, October 9, 1970.

225 Holmes to Hughes, October 9, 1970.

226 Holmes to Hughes, October 9, 1970*.*

227 Holmes to Hughes, October 9, 1970*.*

228 Delauche, “Notes on the Sites Committee meeting held at Munich, Germany, Sunday–Monday, 25–26 October 1970,” in KP, MSS.Eng.c.2436.

229 Delauche, “Notes on visits to the Institut für Plasmaphysik in Garching and the Martinsried Complex,” p. 2, in KP, MSS.Eng.c.2436.

230 Kendrew to Hofschneider, December 10, 1970, in KP, MSS.Eng.c.2436.

231 Delauche, “Notes on the Sites Committee meeting held at Munich, Germany, Sunday–Monday, 25–26 October 1970,” in KP, MSS.Eng.c.2436.

232 In November 1970 the EMBC established four Working Groups. Their task was to study, respectively, the role (Working Group I, chairman: J. F. Embling), the organization, structure, and administration (Working Group II, chairman: C. Zelle), the site (Working Group III, chairman: A. Rörsch), and the financial aspects of the EMBO Laboratory (Working Group IV, chairman: A. Alline). For the purposes of this article, I will focus primarily on EMBC Working Group III.

233 “Memorandum to Working Group III of the EMBC–Criteria to be considered when selecting a site for the proposed EMBO Laboratory,” December 1970, p. 3, in KH, 112 P-HOL-C (2 of 2), *EMBC Sites Committee–Correspondents N–Z.*

234 “Memorandum to Working Group III of the EMBC–Criteria to be considered when selecting a site for the proposed EMBO Laboratory,” December 1970, p. 3.

235 “Memorandum to Working Group III of the EMBC–Criteria to be considered when selecting a site for the proposed EMBO Laboratory,” December 1970, p. 4.

236 “Memorandum to Working Group 111 of the EMBC–Criteria to be considered when selecting a site for the proposed EMBO Laboratory,” December 1970, p. 6.

237 “Memorandum to Working Group 3 of the EMBC–Criteria to be considered when selecting a site for the proposed EMBO Laboratory,” December 1970, p. 6.

238 “Memorandum to Working Group 3 of the EMBC–Criteria to be considered when selecting a site for the proposed EMBO Laboratory,” December 1970, p. 10.

239 The meeting was held in Brussel, on January 8–9, 1971. Participants: Rörsch, Akoyounoglou, Brieskorn, Favre, Maaløe, Tuppy. EMBO advisors: Holmes, Huxley, and Luzzati. See “EMBC, Interim report of Working Group III to the Steering Committee of Working Groups of the EMBC on the EMB Laboratory proposal, January 9, 1971 (CEBM/Lab/71/5),” in KH, 112 P-HOL-C (2 of 2), EMBC Sites Committee–Correspondents N–Z.

240 The EMBC Working Group III included A. Rörsch (chairman and delegate of the Netherlands), G. Akoyunoglou (Greece), H. Brieskorn (West Germany), C. Favre (Switzerland), O. Maaløe (Denmark), H. Tuppy (Austria), F. Graziosi (Italy). In June 1966, Athanasios E. Evangelopoulos (State and University Maternity Hospital “Alexandra,” Athens) offered a site for the EMBO Laboratory as part of the newly established Hellenic Foundation for Fundamental Biological Research “Alexander Fleming,” largely funded by Lady Amalia Fleming (Sir Alexander Fleming’s widow since his death in 1955). At the time the proposal was politely rejected by all EMBO Council members. A similar offer was relaunched in September 1969 by P. Demopoulos (President of the Greek Nuclear Research Center “Demokritos”) and again in September 1970 by Lady Fleming herself, in both cases without success. See documentation in KP, MSS.Eng.c.2436.

241 “EMBC, Remarks of the chairman of the Site Working Group in connection with the document submitted to the Working Group by EMBO, January 1971 (CEBM/Lab/71/2),” in KH, 112 P-HOL-C (2 of 2), *EMBC Sites Committee–Correspondents N–Z.*

242 “EMBC, Report on the criteria to be considered when selecting a site for the proposed EMBO Laboratory, January 1971 (CEBM/Lab/71/4),” in KH, 112 P-HOL-C (2 of 2), EMBC Sites Committee–Correspondents N–Z.

243 In November 1970, the EMBO Council had decided that, in view of the establishment of the EMBC Working Groups on the Laboratory, and the appointment of EMBO consultants to these, both the EMBO Laboratory Committee and the Sites Committee should be disbanded and replaced by the “ad hoc Laboratory Committee.” Members of this Committee were Maaløe, Huxley, Weissman and all the consultants appointed by EMBO to the EMBC Working Groups (Brenner, Mordhay Avron, Alfred Gierer, Reichardt, Holmes, Luzzati, Marianne Grunberg-Manago, Alexander Keynan). See “Minutes of the 15^th^ EMBO Council Meeting held at CERN, Geneva, on November 25 and November 28, 1970,” p. 4, in EMBO Archive, EMBO Council Secretariat Material, 15^th^ EMBO Council Meeting, EMBO SEC-A-21.

244 “EMBO, Report on a Selection of Sites in Germany, prepared by a working party of the EMBO Sites Committee, 22 February 1971,” p. 2, in KH, 112 P-HOL-C (2 of 2), EMBC Sites Committee–Correspondents N–Z.

245 The day before the visit, Niels Jerne, newly appointed chairman of the EMBO Council, wrote to the EMBC President and Swiss delegate, Henri Voirier, to invite Switzerland to propose a site for the EMBO Laboratory: “The scientists of the Council of EMBO—he claimed—as well as those of its Laboratory Committee, have always been unanimous in their opinion that a site in Switzerland, particularly in the proximity of CERN in the Canton of Geneva, would afford the optimal opportunities for the scientific development of a European Laboratory of Molecular Biology.” Jerne to Voirier, January 28, 1971, report in Voirier’s note to the Division for Science and Research of the Federal Department of Internal Affairs, February 5, 1971, in BAR, E 2003 A 1984/84, BD: 278, o.144.511, Laboratoire européen de biologie moléculaire, 1970/1972.

246 Born in 1934 in Heidelberg, Bujard completed his PhD in organic chemistry in Göttingen before moving to the US in 1962 for postdoctoral training with Charles Heidelberger and Gobind Khorana in Madison. Two years later, he was offered an assistant professorship at the Southwest Center for Advanced Studies in Dallas. In 1970 he was appointed professor of molecular genetics in the Faculty of Biology at Heidelberg University, where he began collaborating with Peter von Sengbusch. Born in Riga in 1939, Sengbusch had just returned to Germany after postdoc training at the Biophysics Laboratory in Madison and at the Salk Institute in San Diego, to join Holmes’s laboratory at the MPI for Medical Research. On this see Harrison (2025). Note also the draft title, “A possible site for EMBO in Heidelberg,” signed by Holmes and Sengbusch, in KH, 112 P-HOL-C (2 of 2), EMBC Sites Committee–Correspondents N–Z.

247 “EMBO, Report on a Selection of Sites in Germany, prepared by a working party of the EMBO Sites Committee, 22 February 1971,” p. 3.

248 “EMBO, Report on a Selection of Sites in Germany, prepared by a working party of the EMBO Sites Committee, 22 February 1971,” p. 8.

249 “EMBO, Report on a Selection of Sites in Germany, prepared by a working party of the EMBO Sites Committee, 22 February 1971,” p. 8.

250 “EMBO, Report on a Selection of Sites in Germany, prepared by a working party of the EMBO Sites Committee, 22 February 1971,” p. 7.

251 Rörsch vividly reported this opposition in a letter to Kendrew, by referring to the “Zachau–Holmes controversy:” “I also would be very happy if you could convince Zachau that he should come as a consultant instead of Ken. I think, however, that he is strongly avoiding this, just because he is afraid being [sic] involved in the recommendation that the Working Group is going to make. Probably he wants to hold the position that he can say to his Munich–maffia–members ‘I could not help it; It was all the work of the Cambridge–EMBO–maffia’. Well, I can understand this attitude up to a certain degree, however, in his case, I really want him at the next meeting.” Rörsch to Kendrew, May 20, 1971, in KP, MSS.Eng.c.2446.

252 C. Favre, “Rapport sur la réunion du groupe de travail III de l’EMBC des 8 et 9 Janvier à Bruxelles sur ‘L’étude du site du laboratoire de l’EMBO proposé’. Confidentiel,” January 12, 1971, p. 3, in BAR, E 2003 A 1984/84, BD: 278, o.144.511, Laboratoire européen de biologie moléculaire, 1970/1972 (my translation).

253 Voirier, “Note au Chef de la Division des organisations internationales. Laboratoire européen de biologie moléculaire,” April 7, 1971, p. 3, in BAR, E 2003 A 1984/84, BD: 278, o.144.511, Laboratoire européen de biologie moléculaire, 1970/1972 (my translation).

254 Zachau to Rörsch (and cc Maaløe, Kendrew, Holmes, and Luzzati), March 11, 1971, in KP, MSS.Eng.c.2446.

255 Holmes to Rörsch, March 15, 1971, in KH, 112 P-HOL-C (2 of 2), EMBC Sites Committee – Correspondents N–Z.

256 Holmes to Rörsch, March 15, 1971.

257 Holmes to Rörsch, March 15, 1971.

258 Holmes to Rörsch, March 15, 1971.

259 “EMBC Working Group ‘Site of the Laboratory,’ Second interim report to the Steering Group of the EMBC on the proposed laboratory, April 1971 (CEBM/Lab/71/17),” p. 4, in KH, 112 P-HOL-C (2 of 2), EMBC Sites Committee–Correspondents N–Z. Holmes and Luzzati were EMBO consultants.

260 C. Favre, “Rapport de la réunion du groupe du site de la CEBM, München, 17/18 mars 1971,” in BAR, E 2003 A 1984/84, BD: 278, o.144.511, Laboratoire européen de biologie moléculaire, 1970/1972 (my translation).

261 Kendrew to all members of EMBO Council and Laboratory Committee, March 25, 1971, in KP, MSS.Eng.c.2427.

262 Observers were Voirier (EMBC President), Rörsch (Chairman of EMBC Working Group III), and Brieskorn (EMBC delegate for West Germany).

263 Jerne to Rörsch, April 6, 1971, in KP, MSS.Eng.c.2446.

264 Jerne*,* “Interim Report. Visit of the Second Working Party to Heidelberg on 1^st^ April 1971, arranged by EMBO Council, April 5, 1971,” p. 6, in KP, MSS. Eng.c.2446.

265 Jerne*,* Interim Report. Visit of the Second Working Party to Heidelberg on 1^st^ April 1971, arranged by EMBO Council, April 5, 1971,” p. 6.

266 Jerne*,* Interim Report. Visit of the Second Working Party to Heidelberg on 1^st^ April 1971, arranged by EMBO Council, April 5, 1971,” p. 7.

267 “Minutes of the 16^th^ EMBO Council meeting held at CERN, Geneva, on 26^th^ April 1971,” p. 2, in EMBO Archive, EMBO Council Secretariat Material, EMBO SEC-A-22, https://archive.embo.org/index.php/embo-council-meeting-at-cern-september-1970.

268 Kendrew to Perutz, May 17, 1971, in KP, MSS.Eng.c.2446.

269 “Proposal by the Federal Republic of Germany for the site of a European Laboratory of Molecular Biology–Heidelberg,” in KH, 112 P-HOL-C (2 of 2), EMBC Sites Committee–Correspondents N–Z.

270 “Programme for the visit to Heidelberg by the EMBC Working Group “Site of the Laboratory”, 12 to 14 July 1971, July 8, 1971,” in KH, 112 P-HOL-C (2 of 2), EMBC Sites Committee–Correspondents N–Z. On this occasion Wyman was the EMBO observer.

271 “Comparative study of three possible sites for the proposed European Molecular Biology Laboratory: Garching, Martinsried and Heidelberg, Annex II to document CEBM Lab 71/26 (CEBM Lab 71/27),” in EMBO Archive, EMBO Laboratory Committee Materials, Report Rendered to the European Molecular Biology Conference, EMBO EMBOLAB-B-3-20, https://archive.embo.org/index.php/report-rendered-to-the-european-molecular-biology-conference.

272 Appleyard to Holmes, June 11, 1971, in KH, Box 110 P-HOL-A (1 of 2) Correspondents: A-M.

273 “Written comments submitted by Prof. Zachau to the third meeting of Working Group III,” Annex to “Summary record of the third meeting of EMBC Working Group III ‘Site of the Laboratory,’ Heidelberg, July 13–14, 1971,” in KH, 112 P-HOL-C (2 of 2), EMBC Sites Committee–Correspondents N–Z.

274 “Written comments submitted by Prof. Zachau to the third meeting of Working Group III.” The other members of the Working Group III reaffirmed that “more important than scientific environment was the immediate proximity of a strong physics institute well placed to cooperate,” as existed in Heidelberg. They also dismissed Zachau’s other concerns: “different people want different things,” they noted, and “Heidelberg had its own different kind of attractions.” See “Summary record of the third meeting of EMBC Working Group III ‘Site of the Laboratory,’ Heidelberg, July 13–14, 1971 (CEBM/Lab/71/29), pp. 1–2.

275 Huxley to Rörsch, August 9, 1971, in KP, MSS.Eng.c.2446,

276 Huxley to Kendrew, July 8, 1971, in KP, MSS.Eng.c.2446,

277 In May 1971, after consultation with the DES, Kendrew responded to B. T. M. Willis, from the Harwell Atomic Energy Research Establishment, that “for various political reasons” there was no chance for an EMBO Laboratory in Culham: see Kendrew to Willis, May 20, 1971; Kendrew to A. E. Hickinbotham (DES, Science Branch), May 17, 1971, in KP, MSS.Eng.c.2436.

278 Anthony Tucker, “Britain Loses Laboratory,” *The Guardian*, September 8, 1971, newspaper clipping without pagination. See also Anon., “EMBO not for Culham,” *Nature*, 233, September 17, 1971, pp. 154–155, newspaper clipping. All in KP, MSS.Eng.c.2438.

279 Weisskopf to Kendrew, September 1, 1970, in KP, MSS.Eng.c.2436.

280 Rörsch to Kendrew (cc Maaløe, Luzzati, Holmes and Jerne), March 25, 1971. According to Rörsch’s report, Bannier considered it unlikely that Kees Zilverschoon, head of CERN instrumentation division, would have been “very happy” about another organization requesting “the attentions of his scientists and engineers.”

281 Jerne to Jentschke, April 28, 1971, and Jerne to Kendrew, April 29, 1971, in KP, MSS.Eng.c.2446.

282 Jerne to Kendrew, May 13, 1971, in KP, MSS.Eng.c.2446.

283 Jerne to Kendrew, May 13, 1971.

284 Jacob to Kendrew, June 9, 1971, in KP, MSS.Eng.c.2446. Emphasis in the original.

285 Voirier, “Note au Chef du Département. Visite de M. Scheel. Offre de site de la RFA pour un laboratoire européen de biologie moléculaire,” May 12, 1971, p. 2, in BAR, E 2003 A 1984/84, BD: 278, o.144.511, Laboratoire européen de biologie moléculaire, 1970/1972 (my translation).

286 Voirier, “Note au Chef du Département. Visite de M. Scheel,” p. 2.

287 Voirier, “Note au Chef du Département. Visite de M. Scheel,” p. 3.

288 On March 15, 1972, at the 71^st^ MPG Senate meeting, it was emphasized that within the EMBC there was “concern that the proximity of Dachau, in the Munich area, might be unacceptable for Jewish scientists.” Records of the 71^st^ Senate meeting, March 15, 1972, Archives of the Max Planck Society (AMPG), II. Abt, Rep. 60, No. 71.SP, fol. 14, pp. 159–160. Quoted in Kraft, Renn, Sachse, and Schöttler (2024, p. 697), and Sachse (2025, p. 70).

289 “Speech by J. C. Kendrew for ceremony of signature of Agreement establishing the EMB Laboratory – Geneva, 10 May 1973,” p. 1, in KP, MSS.Eng.c.2459. In September 1971, Kendrew had privately given this same version of the story to Arnulf K. Esterer, who was working on a biography of Szilard: “The history is roughly as follows. The first informal discussions about the possibility of setting up an international laboratory and a programme of fellowships and summer courses, took place in Geneva in December 1962.Those presents were Professor V. Weisskopf, Professor Leo Szilard, Dr J. D. Watson and myself.” See Kendrew to A. K. Esterer, September 29, 1971, in KP, MSS.Eng.c.2418. In 1968, Kendrew (1968, p. 840) recalled that in “autumn 1962” Szilard and Weisskopf, together with the Italian physicist Gilberto Bernardini, proceeded “on the telephone” to contact “a number of molecular biologists interested, including Francis Crick, Sydney Brenner, Jim Watson and myself.” On the role of Gilberto Bernardini and Adriano Buzzati-Traverso, see Cassata (2015, pp. 558–560).

290 Weisskopf was invited to the inauguration of the EMBL but could not attend. In a letter to Kendrew, he restated the origin myth: “I remember with great emotion in 1963 when the whole idea began in my office at CERN. I do believe that we can ascribe the main initiative to Leo Szilard and I hope that his contribution will be appreciated during the festivities. I am very sorry that I cannot come to this great event but I want to let you know how much I am impressed by the existence of a new European collaborative effort in pure science. The idea of Europe as an intellectual and political entity must go on and must grow in spite of all the counter–tendencies that exist today. Europe is the craddle [sic] of everything that we consider great and worthwhile, of everything that makes our lives worth living, be it science, art or philosophy. Let your lab be a symbol of this.” See Weisskopf to Kendrew, March 2, 1978, in KP, MSS.Eng.c.2468.

291 Gertrude Weiss Szilard to Kendrew, October 9, 1978; Weiss Szilard to Kendrew, December 20, 1978, in KP, MSS.Eng.c.2467.

292 “The dolphin. Szilard used this as a personal symbol, and wrote a famous book of essays called ‘The Voice of the Dolphins’. Dolphins have some connection also with my own personal research:” Kendrew to William Furbisher Esq. (Design Research Unit, London), January 23, 1979, in KP, MSS.Eng.c.2467.

293 According to James Watson’s testimony, Szilard was not present at the meeting: see interview between Watson and Georgina Ferry, Merton College Oxford, May 19, 2013, in Ferry (2014, p. 30). By mid–December Szilard had returned to Washington DC: see Lanouette and Silard (1992, p. 460).

294 Weisskopf to Appleyard, July 20, 1962, in CERN Archives, CERN Directors–General (First series), External Relations–International Centre for Biology (EMBO), Correspondence–Documents, EMBO–European Molecular Biology Organization (1962–1964), B12, DG-20683.

295 Weisskopf to Buzzati-Traverso, September 14, 1962, in CERN Archives, CERN Directors–General (First series), Chronological File of Correspondence, DG–CHRONO. See Cassata (2015, pp. 558–559).

296 “Speech by J. C. Kendrew for ceremony of signature of Agreement establishing the EMB Laboratory–Geneva, 10 May 1973,” p. 4.

297 From this perspective, the boundary work between EMBO and the European Cell Biology Organization (ECBO)—a private foundation established in 1969 with funds from Dutch and Swiss firms, headed by Pieter J. Galliard—is revealing. During the negotiation of the EMBO Laboratory project, ECBO approached EMBO about the project. Appleyard’s note of a conversation with ECBO’s representatives, on September 11, 1970, opens with these eloquent comments: “It is clear that relations between ECBO and EMBO are difficult and that the fact is recognized on both sides. Notably, we are a restricted pressure group difficult to get into: they have an open membership. We are thoroughly established financially: they are not. We are rapidly invading their field which really includes ours. In the light of this, the conversation had to be restricted for the most part to polite gossip and generalities on both sides and not very much concrete emerged from it.” See Appleyard, “Note of conversation with the Chairman (Prof. Gaillard) and Vice-Chairman (Prof. Chevremont) of ECBO, September 11, 1970, in KP, MSS.Eng.c.2420.

298 Draft of Slater’s speech, April 24, 1978, in KP, MSS.Eng.c.2468.
